# Foraging for the self: Environment selection for agency inference

**DOI:** 10.3758/s13423-022-02187-w

**Published:** 2022-10-11

**Authors:** Kelsey Perrykkad, Jonathan E. Robinson, Jakob Hohwy

**Affiliations:** 1 Cognition and Philosophy Lab, Philosophy Department, School of Philosophical, Historical and International Studies, Monash University, 29 Ancora Imparo Way, Clayton, VIC 3800 Australia; 2 Monash Centre for Consciousness & Contemplative Studies, Monash University, Melbourne, Victoria Australia

**Keywords:** Environmental niche selection, Environment complexity, Agency, Autism, Prediction error

## Abstract

Sometimes agents choose to occupy environments that are neither traditionally rewarding nor worth exploring, but which rather promise to help minimise uncertainty related to what they can control. Selecting environments that afford inferences about agency seems a foundational aspect of environment selection dynamics – if an agent can’t form reliable beliefs about what they can and can’t control, then they can’t act efficiently to achieve rewards. This relatively neglected aspect of environment selection is important to study so that we can better understand why agents occupy certain environments over others – something that may also be relevant for mental and developmental conditions, such as autism. This online experiment investigates the impact of uncertainty about agency on the way participants choose to freely move between two environments, one that has greater irreducible variability and one that is more complex to model. We hypothesise that increasingly erroneous predictions about the expected outcome of agency-exploring actions can be a driver of switching environments, and we explore which type of environment agents prefer. Results show that participants actively switch between the two environments following increases in prediction error, and that the tolerance for prediction error before switching is modulated by individuals’ autism traits. Further, we find that participants more frequently occupy the variable environment, which is predicted by greater accuracy and higher confidence than the complex environment. This is the first online study to investigate relatively unconstrained ongoing foraging dynamics in support of judgements of agency, and in doing so represents a significant methodological advance.

**Supplementary Information:**

The online version contains supplementary material available at 10.3758/s13423-022-02187-w.

## Introduction

Agents take an active part in choosing their environment to maximise information gain or to fulfill their needs and desires: that is, to achieve epistemic and pragmatic goals. However, in some cases, agents pick an environment not for the reward it offers or even the information about it they can gain, but because it supports agent-focused epistemic foraging; they pick environments allowing them to manage uncertainty about action-outcome contingencies. To illustrate, consider someone turning down an exciting, well-paid promotion, not for fear of the responsibility that comes with increased decision-making powers, but because they are not confident they will be able to discern the power structures closer to the top of the management hierarchy; they know they will be in control, but the mapping of actions to outcomes threatens to be ambiguous – so they stay put. In such ambiguous contexts, when performing an action and watching what happens, individuals struggle to judge whether perceived consequences should be rightly attributed to their action. This leads to a lack of confidence in their ability to harness the control afforded by the more powerful position to realise their intentions. The decision to forgo available rewards for this reason can seem perplexing to outsiders, but there has been little research on these types of drivers for environment selection. In this study, we therefore explore how participants select environments to forage for agency information.

Primarily, we focus on how agents choose which environments to occupy, and when to switch between available environments, when they are tasked with making a judgement of agency. When exploration and foraging studies traditionally consider the effect of uncertainty on decisions about occupying environments, they are focused on uncertainty in the availability, stability, and distribution of pragmatic rewards across available environments (Constantino & Daw, 2015; Hutchinson et al., 2008; Kacelnik & Bateson, 1996; Mehlhorn et al., 2015). There is growing acknowledgement that, under a broad conception of reward, resources driving such decisions can include information gain or uncertainty reduction in addition to more traditional pragmatic rewards (Cohen et al., 2007; Friston et al., 2015; Inglis, 2000; Inglis et al., 2001; Pezzulo & Friston, 2019). This provides a richer perspective on environment selection behaviour, where an animal might forego traditional rewards in order to forage a different patch for information. Decreasing certainty in the current environment, and expected increased certainty in the next one, may therefore drive a shift between environments.

Existing research has not focused much on how beliefs about one’s own agency contribute to environment decisions. Beliefs about agency consist in judgements about the degree to which one can effectively act to bring about expected sensory consequences in the current environment, and uncertainty about agency then relates to factors that undermine the agent’s ability to infer what they can efficiently control. There will be relatively little uncertainty about agency when foraging in an environment where all areas are easily accessible, and rewards are easy to collect when encountered. Uncertainty mounts when the mapping from actions to outcomes becomes ambiguous due to variability in the control states of the agent, such as cramps in their limbs or defects of their tools, or inability to ascertain the causal impact of their actions in the environment. The upshot is that increasing uncertainty about agency impedes successful action for either epistemic or pragmatic gain. While successful agency is in itself rewarding (Inglis, 2000), this approach contrasts with research focusing on uncertainty in choosing patches for foraging due to variability or volatility in the availability and depletion of extrinsic rewards.

The relationship between judgements of agency and ongoing environment selection is significant because it may throw new light on otherwise perplexing foraging decisions, where agents forego both pragmatic and informational rewards. This may speak to maladaptive symptoms of some mental conditions (Addicott et al., 2017), which often relate to action-outcome contingencies and inferred agency. Examples include learned helplessness as a model for depression (Vollmayr & Gass, 2013), delusions of control or negative symptoms such as avolition in schizophrenia (Bentall et al., 2010; Jeannerod, 2008; Synofzik & Voss, 2010), incorrect source attribution and intense responses to arbitrary sensory cues in post-traumatic stress disorder (Linson et al., 2020), and repetitive behaviours in autism spectrum condition (autism) (Perrykkad & Hohwy, 2020a). In other words, it may be that agents occasionally choose environments that promote unambiguous agency beliefs, over more rewarding or informative environments. This can in many cases be rational since highly precise agency beliefs afford efficient pursuit of other rewards.

In the experiment presented here, participants are tasked with determining which of four squares they can control on the screen with their mouse. Notably, participants have relatively unconstrained freedom in how they move and can dynamically close the action-perception loop in real-time (Perrykkad et al., 2021). This is in contrast to most previous agency experiments that restrict participants’ action repertoire to, for example, pressing one of two buttons (or one designated button at a time of their choosing) and being presented with one piece of sensory feedback per trial (e.g., in intentional binding paradigms; see Bednark et al., 2015; Haggard et al., 2002). Giving participants this freedom is integral to realistically capture ongoing dynamics related to environmental selection in the context of agency inference, but to our knowledge no previous work has investigated this.

Here, the onscreen space in which participants move is separated into two equally-sized environments. This allows us to ask whether agents needing to judge their own agency under uncertainty will favour certain environments over others (*environment selection*), and what may motivate agents to abort foraging for agency information in one environment and shift to another (*environment switching*). To answer these questions specifically for agency beliefs, we distinguish our task from standard foraging tasks, such that there is no decreasing rate of resources in our environments. Instead, we measure the incidental accumulation of uncertainty about agency, conceived as prediction error in the control of latent causes. Differences between what is expected and what happens (prediction error) following action is central to our current understanding of how humans infer agency (David et al., 2008; Perrykkad et al., 2021). In measuring a behavioural proxy for prediction error (bPE), we hypothesise that agents will switch environments when this kind of prediction error increases (Cohen et al., 2007, p. 937; Mirza et al., 2018). We also hypothesise that agents will on average spend more time foraging in environments that afford more accurate agency beliefs.

The environments presented to participants in this experiment are balanced in terms of how much bPE will accumulate but differ in underlying statistics and causal structure. One environment (‘sand’) is characterised by a relatively high level of irreducible variability in the opportunity for control, the other environment (‘water’) has less irreducible variability but instead is more causally complex. We aim to determine which type of environment best facilitates accurate judgements of agency, and which agents prefer. Variability and complexity are two factors that impact environment decisions in general, but we did not have a hypothesis for how participants would weigh variability against complexity in environments to support judgements about their own agency. The displacement of the mouse on each presented frame was, in fact, identical between the two environments. If participants ignored the existence of the structure of the water environment (and did not attempt to cognitively model it), their experience would be identical to the sand environment. So, without knowing whether participants prefer to discern agency under conditions of irreducible uncertainty or in an environment that invites greater model complexity, neither environment could be deemed inherently more difficult.

As mentioned, environment selection dynamics may be particularly relevant for understanding behaviour in particular psychiatric conditions (Addicott et al., 2017). To begin exploring this, we consider autism traits, predicting that participants higher in autism traits will prefer to avoid the ‘water’ environment, which calls for more complex, hierarchically deep internal models (Constant et al., 2020; Perrykkad & Hohwy, 2020b). Assuming autism traits are associated with models that struggle with greater uncertainty, we predict that participants with more autism traits will tolerate less bPE before switching environments (Perrykkad et al., 2021). The contrast between causally complex and simple but variable environments might be especially stark for autistic individuals due to previously identified differences in sensory processing that are sensitive to stimulus complexity for this group (Haigh, 2018; O’Connor, 2012; Van De Cruys et al., 2019).

The factors discussed here are, of course, also required for skill acquisition. Learning to efficiently obtain pragmatic rewards, navigate an environment, or optimally seek information (as in epistemic foraging) may all be considered skills that are supported by selecting environments that reduce uncertainty related to expected action-outcome contingencies. In this task, the overriding consideration for the participants’ actions is to support the judgement of agency at the end of each trial. They did so by selecting the square they thought they controlled (or selecting that they controlled no square), followed by serially reporting their sense of agency and confidence in their judgement on Likert scales. Thus, the participants’ chief focus was on garnering information through their actions to make these responses accurately. Making these judgements accurately is a fundamental precondition to acquiring more traditional kinds of skill described above, though both rely on developing precise action-outcome expectations. Importantly, one can also finesse their skill in judging their own agency, or appropriately identifying their own actions as the cause of incoming sensory signals. The accuracy measures of this study capture this fundamental skill. Only if participants are proficient at identifying what they have agency over can they effectively exercise their agency to develop other skills. Despite being essential to all other forms of skill acquisition, this aspect is often overlooked.

Understanding how individuals forage for agency-related information can ultimately help us better understand what drives agents’ sometimes perplexing decisions about which environments to occupy and when to change environments.

## Methods

This study was approved by Monash University Human Research Ethics Committee (Project Number 26240) and was conducted in accordance with the relevant guidelines and regulations. All participants agreed to informed consent documents upon commencing the protocol.

### Participants

A total of 229 participants were paid for completion of the study, 129 were recruited from Amazon Mechanical Turk using the Cloud Research platform (formerly TurkPrime (Litman et al., 2017)), and 100 were recruited from Prolific (http://www.prolific.com (‘Prolific’, 2019)). Participants were paid $4.50 USD (Amazon Mechanical Turk) or £4.10 GBP (Prolific) for completing the task, which took a median of 52 min to complete (including consent process and self-timed breaks, range: 26–146 min total duration). Eligibility criteria included being fluent in English, aged 18–50, with no history of head injuries or neurological damage, normal or corrected-to-normal vision and no regular use of prescribed or unprescribed medication that may affect cognitive functioning.

One of the major strengths of this study is running a dynamic, interactive task in an online environment. However, doing so successfully requires special attention to be paid to issues of data quality. We opted to use PsychoJS for stimulus presentation owing to its low latency and high precision timing (Anwyl-Irvine et al., 2020; Bridges et al., 2020). Despite this, an independent pilot sample of 22 online participants demonstrated that, due to the computing/internet demands of the task, significant frame delays were present for many participants (pilot timing data freely available on Figshare: 10.26180/19252082). Based on data from the pilot, we chose stringent exclusion criteria to ensure relatively stable presentation in the final dataset. Individual trials were defined as ‘bad’ if less than 50% of the programmed frames were presented, the average frame rate during non-lagged periods was less than half the rate expected, participants never pressed a square selection button, or participants moved the squares for less than 1% of the trial. These trials were removed from analysis for all participants and having low remaining trial numbers excluded participants’ data entirely.

In total, 148 participants were excluded from the final dataset for one or more of the following reasons: incomplete dataset recorded due to technical difficulties (*n* = 14), disqualification due to report of drug abuse in demographics survey (*n* = 1), average accuracy less than 25% (chance = 20%, *n* = 53), or if more than 50% of trials were defined as bad (*n* = 127; see criteria above). While this means that 65% of our collected sample was excluded from further analysis, this is almost entirely due to the difficulties of running a dynamic task that closes the action-perception loop in an online environment. 95% of the excluded participants had technical or internet related issues which lead to a significant number of dropped or lagging frames, or otherwise missing data. Thirty-five percent of the excluded participants had close to or below chance performance, which overlapped with the participants who had technical issues, but is also more likely in an online environment when task engagement cannot be monitored in person. While we are confident that the final dataset is of reasonable quality, we also include fixed effects related to presented frame rate in our statistical analyses to ensure we statistically control for technical variability.

Of the final dataset, nine participants reported diagnosed mental conditions (Attention-deficit hyperactivity disorder (ADHD): *n* = 2, Anxiety: *n* = 3, Depression: *n* = 2, Post-traumatic stress disorder (PTSD): *n* = 1, Dyslexia: *n* = 1) but were not excluded from the study. Forty-one participants out of the final dataset of 84 (see Online Supplementary Materials (ESM) Section 1) were from Amazon Mechanical Turk, and 43 from Prolific.

### Procedure

Following the informed consent procedure, participants completed a general demographics survey, followed by the Autism-Spectrum Quotient (AQ) (Baron-Cohen et al., 2001) and Self Concept Clarity Scale (SCCS) (Campbell et al., 1996). Then they were forwarded to Pavlovia (http://pavlovia.org) to complete the agency task. Finally, they completed the Subthreshold Autism Trait Questionnaire (SATQ) (Kanne et al., 2012) and were compensated via a completion code or link. To ensure consistency in participant experience, participants were asked to complete the task using a Chrome or Firefox browser, using a laptop or desktop and with an external mouse (not laptop trackpad or touchscreen).

#### The Beach Task: Agency Task Design

**Fig. 1:**
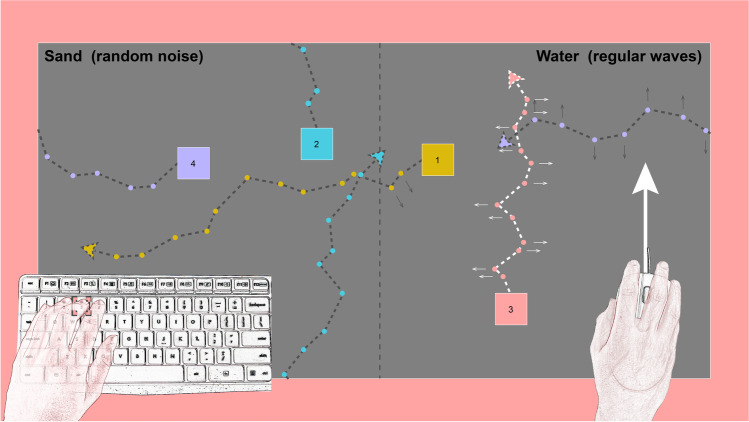
In this illustration, the participant has selected square 3 using their middle finger on the keyboard, which is indicated by the coloured border on screen. The mouse is being moved in a straight line forward, and the correct square (square 3) moves on average in this direction. Participants’ task is to identify this square within 15 s. Variability in direction is added to all squares and is sampled from the same distribution. The other squares move at an angle offset from the mouse position, for example, square 4 moves -90° from the mouse position. The non-selected squares move faster. In this diagram, each dot represents a square position over a short period of time, and regular waves are depicted on the right half of the screen with small arrows indicating the forced direction at that time. Waves apply in the same direction for all squares in that environment on that frame, relative to the heading of the square. Had they been selected, squares 1 and 4 would count as having switched environment during the depicted period, while squares 2 and 3 have not. Please note that relative distances and wave duration depicted here are illustrative. Participants could only see the moving squares, grey background and selected coloured border. The centre boundary and environment labels were not visible

This experiment was a variant of the *squares task* (Grainger et al., 2014; Perrykkad et al., 2021; Russell & Hill, 2001; Williams & Happé, 2009); the most commonly used judgement of agency task used in autistic populations (Perrykkad & Hohwy, 2020b). Stimuli were presented online using PsychoJS (v2020.2) (Peirce et al., 2019). In this version of the task, there were four numbered squares on the screen in each trial (Fig. 1). The squares were randomly coloured with perceived-luminance matched shades of blue, red, purple, and yellow on a grey background. Participants pressed and held a number key to select the relevant square. Square selection was visually represented to the participant by colouring border of the screen (an area that squares couldn’t enter) with the selected square’s colour. If a square was selected, all the squares moved when the mouse was moved and all the squares stopped with a tiny amount of jitter (to ensure participants didn’t think it had frozen) when the mouse stopped, so participants had to both select a square and move in order to accurately complete the task. The selected square moved at half the speed of the other squares. This meant that participants could only gain high precision information on the selected square’s present state but could also gather less precise information from the other stimuli. This square selection function allowed us to capture the participants’ hypothesis at any given moment without using eye-tracking (cf. Perrykkad et al., 2021), and as in foveal pursuit, the selected square would result in the most precise information, though more noisy information was available from other options. All squares moved faster than the actual mouse distance, to allow more screen wraps before the edge is hit, since repositioning the mouse using PsychoJS was not possible. To give participants more freedom of movement when this happens, they could also press ‘Q’ to reposition all squares on the left half of the screen or ‘R’ to reposition all to the right. All square positioning (initial and after these button presses) was random.

Square positions were only updated at 30 Hz, to limit computational performance variability across physical set-ups. In what follows, we refer to a *frame* as one of these 30 Hz stimulus presentation data points. Regardless, refresh rate on monitors across participants was recorded as 60 Hz. Participants were given 15 s to identify the target square which they controlled.

Within each trial, there were two hidden environments. Half the screen (left or right) was randomly assigned *water*, and the other half *sand*. In both environments on each frame, for each square, a sample was taken from a 95% confidence interval for ± 70°. To save online processing costs, four random iterations of trial order and variability sampling for each square on each frame across the whole experiment were pre-established, and randomly selected for each participant. For squares in the *sand* environment, the variability angle was added to the input mouse angle from the participant (and the offset angle if the square is a distracter, described below) to generate the new square location for that frame (the distance moved depended on whether the square was currently selected). For squares located in the *water* environment, the sign of the sampled angle was forced to alternate between positive and negative values every 15 frames, creating regular *waves*, that pushed the participant consistently to the left or right of their heading. Since distribution and sampled magnitude of the variability was the same regardless of the environment, the displacement of the mouse on each frame across the two environments was equal. However, in water, there was an underlying predictable structure to the variability that was not present in sand.

While the correct, controlled square followed the mouse movements with the addition of this variability: distracter squares moved at a random angle offset from the vector of mouse movement. This angle was also independently and randomly changed (and smoothly transitioned) five times in each trial, meaning that each distracter square appeared to turn five times when the participant did not initiate a turn, breaking any illusion of control resulting from motor adaptation. Half of the trials were *no-control* trials in which all four squares were distracter squares. After the 15 s, all squares froze and were numbered, and an unspeeded numerical response was prompted from participants indicating which square they controlled or ‘0’ if they thought they controlled none of the squares. Participants also responded on a 9-point Likert scale to two additional questions asking for ratings of confidence (‘Not at all’, ‘Very confident’) and sense of agency (‘No agency’ to ‘Complete agency’). Participants completed a total of 48 non-practice trials in three blocks of eight agentive trials and eight no-control trials.

An interactive, self-timed block of instructions before these task blocks explained the mechanics of the task and the presence of the two environments and included six timed, full practice trials (three control, three no-control). This served the purposes of both explaining the task and familiarising the participant to the movements of the correct square. Participants were explicitly informed that they would experience some variability in the movement of the squares. With respect to the environments, they were initially given screens filled with one environment at a time and differently coloured. On these screens, they were instructed that in sand, they would experience random jittering in their movement, while in the water, they would be pushed from side to side. Environments were not coloured in the main task (or the full practice trials), which had a grey background with the coloured border indicating the selected square. The colours of the squares during the practice period were different from the main task. During the full practice trials participants were given feedback about the accuracy of their agency judgements, however, the main task had no feedback. Without breaks or computational delays, the judgement of agency task was expected to take 20–25 min.

We included additional measures to mitigate potential inattention in our online sample, including large warnings when participants failed to select squares or move the mouse during a trial, forced full screen at the start of every trial and instructional manipulation check questions during surveys (Oppenheimer et al., 2009). Further, due to variability in computing and internet set ups across participants, there was some variability in the timing of the waves in the final dataset. Participants were presented with an average of 20.59 waves per trial in the water environment with a mode wave time of 478.2 ms per trial (compare to programmed 500 ms). This is considered stable enough for legitimate comparison and reassures us that data quality for the included participants was reasonable.

### Analysis

As outlined above, ‘bad’ trials were removed from analysis. Trials were defined as ‘bad’ if less than 50% of the frames were presented in the 15 s (< 225 frames), the average frame rate during non-lagged periods was less than half the rate expected, participants never pressed a square selection button, or participants moved the squares for less than 1% of the trial.

#### Statistical analysis

All statistical analyses were conducted using Jamovi version 1.6.23 and the GAMLj module (Gallucci, 2019; R Core Team, 2018; The Jamovi Project, 2019). Many of the analyses described below are in the form of mixed linear models. Compared to traditional methods, this approach affords more sophisticated handling of missing and outlying data, thus improving the accuracy, precision, and generalisability of fixed effect estimates (Singmann & Kellen, 2020). For each model, the structure will be specifically described below. Common control covariates for our analyses include number of frames and the standard deviation of wave duration in the water environment (participant averages were used where participant-wise data is used) to account for variability in stimuli presentation quality. Descriptive statistics of data quality and removal are outlined in the ESM (Section 2). In addition, the survey measures are included as continuous fixed effects along with their two-way interactions with other fixed effects in each mixed model below. Since including both SATQ and AQ measures of autism traits in the mixed models would overlap greatly in variance accounted for, we decided to choose only the autism traits measure that was most orthogonal (least correlated) with our other survey measure (SCCS), and report the outcome at the start of the results section. For ease of interpretation, post-hoc tests for interactions with survey measures were simple effects contrasting participants mean scores to those above and below one standard deviation from the mean.

Across all statistical analyses, post hocs are reported with a Bonferroni correction applied to the p-value. For mixed models, the Satterthwaite method for estimating degrees of freedom is used. Post-hoc analyses for categorical contrasts compare estimated marginal means from the base model. Since the Satterthwaite method uses pooled variances and degrees of freedom, the degrees of freedom are higher than might otherwise be expected. In order to validate the changes made due to practical considerations of putting the squares task online, we report ESM (Section 2) that validate the current methods by comparing results from Perrykkad et al. (2021) with the experiment reported here. The primary observed differences in behaviour are a more staccato movement style, driven by the button press function to change selected square. This leads to speed-related artefacts in bPE patterns, which are largely removed in the temporally sensitive analyses below by including speed in each relevant time window as a covariate fixed factor.

#### Accuracy

Since there were equal numbers of trials where the ground truth of agency was present or absent, we computed signal detection theory measures for the agency signal for each participant. These included d’ and criterion. Larger values of d’ indicate greater sensitivity to the presence of a signal and would indicate greater unbiased accuracy on the judgement of agency task. A large positive criterion value implies that the participant requires strong evidence before reporting that they had agency in a trial, while a smaller, negative criterion indicates that the participant is quite liberal with asserting agency. These values were tested using one-sample t-tests against a value of zero, indicating no sensitivity (d’) or no bias (criterion) as the null hypothesis. A Pearson’s correlation with autism traits for each d’ and criterion was also performed to see if judgements of agency varied with these traits.

#### Environmental niche selection

##### Environment bias

Trials were classified according to the *dominant environment* based on the percent of time spent in sand and water in each trial. So, in a sand-dominant trial, participants spent more than 50% of the trial in the sand half of the screen. To quantify an overall preference for one environment over the other, we computed two measures in each trial – environment dominance (binary per trial) and percent of time spent in each environment (continuous). To test for overall environment preferences, one-sample t-tests comparing these to 50% were used. To look at differences in accuracy or bias towards agency judgements depending on environmental preferences, signal detection theory measures split by environmental dominance were compared using paired sample t-tests for each d’ and criterion.

##### Environment selection

We then used two mixed models to look at factors within and following a trial that predicted the percent of time spent in each environment in each trial.

The mixed model looking at how end of trial responses and trial type predict percent of time spent in each environment included fixed effects of sense of agency ratings, confidence, accuracy, judged agency, and trial type. The standard fixed effects of SCCS and autism traits; standard control fixed effects of number of frames and wave time variability; and standard random effect of participant were also included. Only two-way interactions between autism traits, SCCS, and other non-control fixed effects were included in this model.

As one of the within-trial measures, we were interested in participants’ experience of, and responsiveness to, prediction error. Following Perrykkad et al. (2021), bPE was calculated by taking the Euclidean distance between where the selected square would have ended up if it had followed the trajectory of the mouse input (with a constant speed multiplier as described above) and where the square actually went. This means that bPE is largely under the control of the participants, and is influenced by three factors: (1) the speed of movement (determining the distance travelled, longer distances mean more bPE), and the angular changes to the input determined by (2) the uncertainty in the environment and (3) the angular offset if the selected square is not the correct one. In being under the participants’ control, average bPE captures important features of within-trial behaviour, and is included as a possible factor related to environment selection.

The mixed model considering the association between in-trial behaviours and percent of time spent in each environment included fixed effects of percent of frames spent moving, number of hypothesis switches, average bPE, average speed, acceleration, and jerk. The standard fixed effects of SCCS and autism traits, standard control fixed effects of number of frames and wave time variability, and standard random effect of participant were also included. Only two-way interactions between autism traits, SCCS, and other non-control fixed effects were included in this model.

##### Environment switching

We were also interested in the way participants used switching between the available environments as a strategy to minimise prediction error and infer agency.

Our bPE measure allows us to investigate event related behavioural prediction error (by constructing an ERbPE, see Perrykkad et al. (2021)), around the time points when participants switched environments. Since environment switches that occurred concurrently with hypothesis switches could not be conceptually distinguished as a pure ‘environment switch’ strategy rather than primarily a hypothesis switch strategy, these switches were removed from these analyses to isolate the possible use of environment switching as an independent policy. As such, environment switch events here were due either to traversing the centre or outer edges of the screen or pressing ‘Q’ or ‘R’ to switch to the other side of the screen. This was a two-step analysis that (Step 1) specified the relevant time window irrespective of the factors driving these differences and (Step 2) model each factor to measure its unique contribution to the pattern of prediction error observed.

##### Step 1: Time-window specification – real versus faux crossings

In order to first select relevant datapoints for use in further analysis of bPE dataseries across relevant dimensions, we compare bPE around actual environment switches with a control dataseries. To test whether environment switching shows a distinct dataseries of bPE from traversing other regions of the screen, we generate a control time series locked to crossings of random positions on either side of the display. See ESM for further details (Section 4). Average bPE for each frame in an epoch of ± 15 frames (approximating 500 ms at 30 Hz presentation) were taken to generate a true (around true boundary crossing time points) and control (around eligible faux boundary crossings) ERbPE for each participant. Due to the sporadic movement observed in the experiment, the ERbPEs were split into *lead-in* and *lead-out* epochs that were defined by moving the ERbPE epoch trigger, if the event is immediately preceded or followed by periods where nothing is selected, to the first (lead-out) or last (lead-in) time a hypothesis was validly selected. Difference wave sign-flip permutation testing (10,000 permutations) was used to determine frames in which the environment switch ERbPE differed from the control dataseries, and rankings of the true t-values against the maximum t-values from each permuted sample to provide estimated p-values for this analysis (non-parametric maximum pseudo t-test) that implicitly corrects for multiple comparisons. Crucially this analysis did not include additional covariates so that it did not unintentionally bias any of the factors in the subsequent analysis.

##### Step 2: Linear mixed model accounting for speed

To investigate the factors that contribute to differences in the ERbPE for true environment switches, a mixed model was used to compare bPE in the time window selected as significant by the permutation analysis. This model included the fixed factors of time bin and switch direction (to water or to sand). Given that movements were much more disjointed and speed was more variable (in ways we are not interested in) in the online version of the task, average speed in each frame for each participant was included as a covariate control fixed factor. By accounting for speed, this analysis focuses on displacement of the stimuli that is based in only the quality of hypothesis and environmental variability (interested readers are invited to see ESM for a version of the model that does not include speed (Section 6), and one that replaces the dependent variable with speed (Section 7)). The standard fixed effects of SCCS and autism traits, standard control fixed effects of number of frames and wave time variability, and standard random effect of participant were also included. Only two-way interactions between autism traits, SCCS and other non-control fixed effects were included in this model.

## Results

### Surveys

Descriptive statistics for the survey measures are available in Table 1. Based on Pearson’s correlations between the survey measures, AQ was chosen for inclusion in further models, as it was less correlated with SCCS (r = - 0.12, p = 0.27) than SATQ was (r = - 0.25, p = 0.02).

**Table 1:** Survey descriptive statistics

	Mean	Standard deviation	SEM	Range
AQ	20.55	6.84	0.75	8–37
SATQ	26.80	10.88	1.19	5–51
SCC	40.31	10.82	1.18	20–60

### Accuracy

The average accuracy on the task was 54.1% (SEM: 1.72, range: 26.32–87.18), with chance at 20% (four squares or no-control). The signal detection theory analysis showed an average d’ of 0.55 (SEM: 0.097) and an average criterion of - 0.46 (SEM: 0.045). One sample t-tests showed that both d’ (t(83) = 5.68, p = 2.0 × 10^-7^, d = 0.62) and criterion (t(83) = - 10.29, p = 1.8 × 10^-16^, d = - 1.12) were significantly different from zero. Neither d’ nor criterion showed significant correlations with AQ (d’: r = - 0.057, p = 0.61; criterion: r = - 0.018, p = 0.87). This shows that participants were sensitive to agency and had a liberal bias for attributing agency, and these parameters did not vary with autism traits.

### Environmental niche selection

#### Environment bias

Participants spent an average of 49.0% (SEM: 0.039) of each trial in the water environment, and a one-sample t-test showed a small but significant bias towards the sand environment (t(83) = - 2.62, p = 0.010, d = - 0.29). Additionally, 47.5% of trials were classified as water dominant, and a one-sample t-test showed that this too was significantly different from 50% (t(83) = - 2.65, p = 0.010, d = - 0.29). Paired sample t-tests comparing d’ and criterion for water dominant and sand dominant trials showed no effect of environmental dominance (d’: t(83) = - 1.55, p = 0.13, d = - 0.17; criterion: t(83) = 0.74, p = 0.46, d = 0.08).

#### Environment selection

The mixed model used to investigate how time spent in each environment was predicted by responses at the end of the trial and trial type revealed main effects of confidence (F(1, 2955) = 7.58, p = 0.0059) and accuracy (F(1, 2955) = 4.44, p = 0.035). In correct trials, participants spent 1.79% longer in the sand environment. Further, as confidence increases, time spent in sand increases (mean difference of 2.70% between highest and lowest confidence trials).

The mixed model used to investigate how time spent in each environment was predicted by other behaviours during the trial had no significant main effects or interactions.

#### Environment switching

On average, participants switched environments 7.38 times per trial (SEM: 0.11, range: 0–67). Of these, an average of 87.9% (SEM: 0.34) were traverses of the centre line or outer edges, 11.86% coincided with a hypothesis switch (SEM: 17.94, removed from subsequent analyses) and 0.26% were performed using the button press mechanism that moved all squares to a chosen side of the screen (SEM: 0.056).

##### Step 1: Time-window specification – real versus faux crossings

**Fig. 2:**
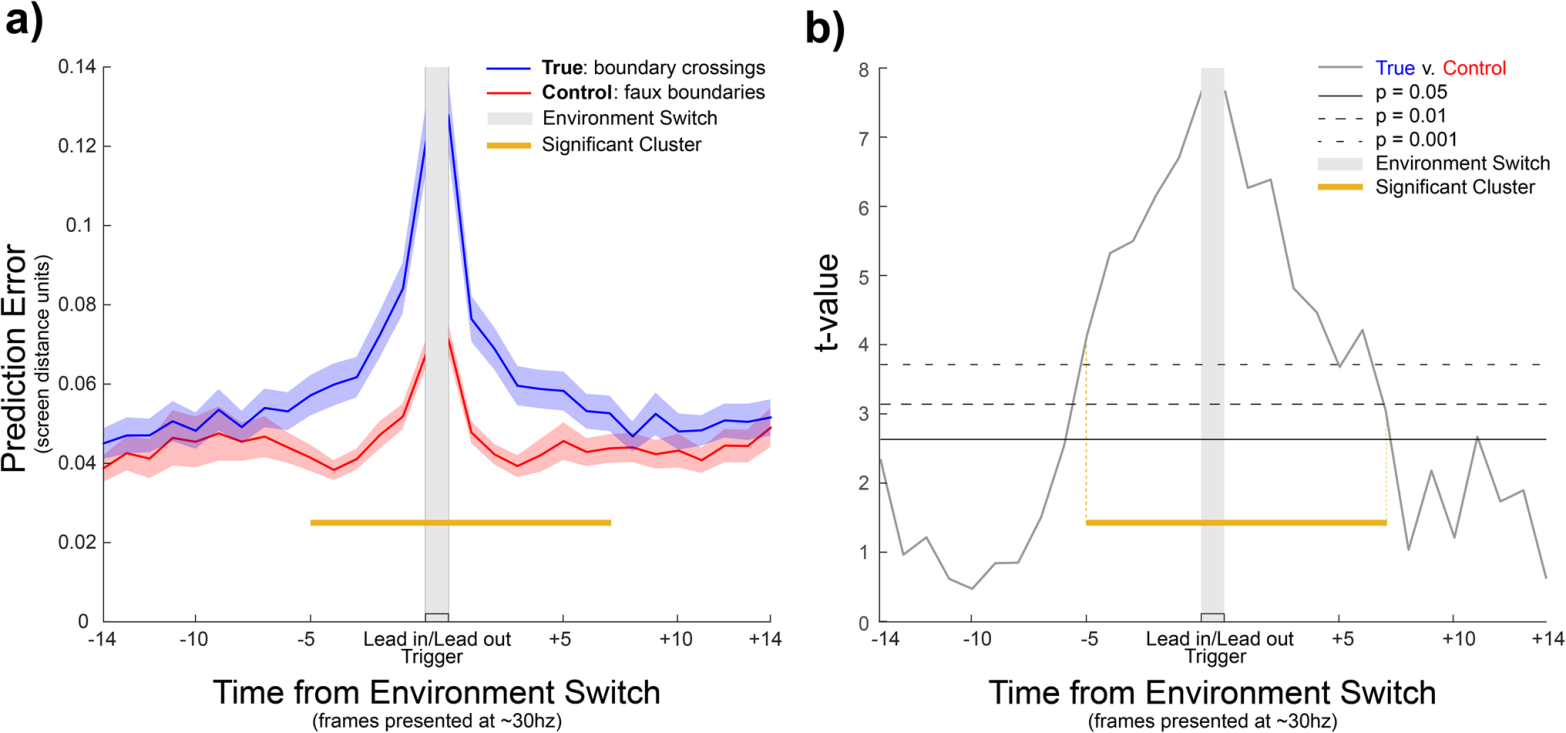
*Environment switch ERbPE and permutation differences from control data*. Panel (**a**) Shaded regions represent standard error of the mean. The blue line (top) depicts behavioural prediction error (bPE) surrounding crossings of true environment boundaries time locked to the grey central period. The yellow bar indicates which of these timepoints is significantly different from a control dataset of bPE surrounding faux boundaries placed randomly between the true environmental boundaries (represented by the red line). Panel (**b**) depicts the t-values at each time point resulting from permutation testing ranking the difference between the blue and red lines in panel a). Significance thresholds are represented by horizontal lines, and the cluster of time points which cross p = 0.05 are chosen for the yellow epoch represented in both panels, which is further analysed

The permutation analysis compared bPE around true environment switches to a control dataset created from faux boundaries to identify any time points that show a distinct pattern of bPE (Fig. 2). In this analysis, the time of the event (both lead-in and lead-out times), five datapoints preceding the lead-in time, and seven datapoints following the lead-out time were identified as significant, which demonstrated these time points surpassed the α = 0.05 permuted t threshold (t_thresh_ = 2.63; see Fig. 2b). Specifically, this analysis indicated that there were larger bPEs in this window around the environment switch than in other locations that participant traversed (mean difference = 0.025, SEM = 0.0010; see Fig. 2a). Note that ancillary testing showed that the identified window is nearly identical if speed is added as a covariate (see mixed model analysis in ESM Section 5).

##### Step 2: Linear mixed model accounting for speed

**Fig. 3:**
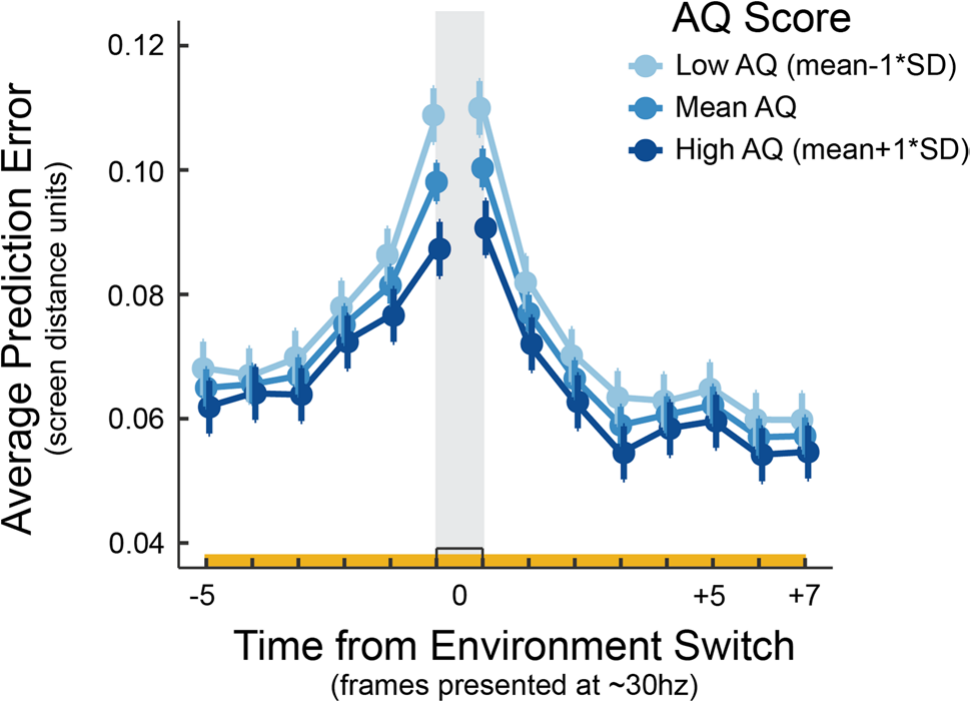
*Environment switch ERbPE split by AQ score. *Error bars represent standard error of the mean. Analysed time period matches the yellow bar in Fig. 2. Behavioural prediction error here is adjusted for mean speed at each time. The low AQ group (scores of 13 or lower; *n* = 13) are represented by the lightest blue, the high autism traits group (scored 28 or higher; *n* = 9) are represented by the darkest blue, and the mean autism group scored between 14 and 27 (*n* = 62)

A mixed model was used to determine which factors influence the pattern of bPE in this window while controlling for speed of movement (Fig. 3). Results showed a significant main effect of time bin (F(13, 2175) = 51.16, p = 6.052 × 10^-116^) and an interaction between AQ score and time bin (F(13, 2168) = 2.32, p = 0.0046). Post hoc analysis revealed that while the time of the event was not different between lead in or lead out value (t(2173) = - 0.83, p = 1.0), both of these values were significantly greater than all other time bins in this window (t-values range: t(2189) = - 6.30 (lead in vs. - 1): t(2211) = 16.04 (lead out vs. + 6)). Significant increases in bPE become apparent two frames preceding this peak, which, while not significantly greater than three frames preceding the switch (t(2175) = - 3.27, p = 0.098), is significantly greater than four frames preceding (t(2174) = - 3.80, p = 0.013). See ESM for all comparisons (Section 8). Behavioural proxy for prediction error drops back to a stable value around two frames following the switch, which is statistically no different from three frames following (t(2174) = 2.98, p = 0.26), but is still decreasing from the frame before (t(2175) = 4.20, p = 0.0026). For the interaction, looking at simple effects of AQ by time bin showed that at both the lead in (F(1, 169) = 12.52, p = 5.21 × 10^-4^) and lead out (F(1, 169) = 10.04, p = 0.0018) times there was a significant effect of AQ, such that AQ score was inversely associated with bPE (Fig. 3). In controlling for speed, this analysis shows that the drivers of the particular pattern of bPE in the time window around environment switches (identified by the previous analysis that was agnostic to sources of prediction error) is driven by other sources of prediction error, namely the variability in the environment and quality of the hypothesis.

## Discussion

Unless agents have security in their ability to manage uncertainty around action-outcome mappings, they cannot be confident they can act successfully. So, before they can act for epistemic or pragmatic gain, it is reasonable to favour an environment that affords agency-focused information foraging. In the experiment reported here, participants were able to freely move between two environments, a variable ‘sand’ environment and a complex ‘water’ environment as they tried to determine which of four squares on the screen they controlled, if any. The results show that participants have a slight preference for the sand environment, which was predicted by greater accuracy and confidence in their judgements of agency. Further, the accumulation of prediction error in the control of movements foreshadows environment switching, and participants with fewer autism traits tolerated more prediction error before they opted to abandon their current foraging context. This study is the first to investigate how prediction error dynamics influence environment selection to support agency inferences, and makes significant methodological advances in moving the task online.

Of the two environments offered to participants in this experiment, there are a priori benefits of each. They were matched for prediction error at the most basic level – that is, the Euclidean distance between where the objects would have ended up had they followed the mouse and where they did end up in each environment was the same. This yields the basic measure of bPE in our results. However, in the sand environment, there was no further structure underlying the variability in the environment. Successfully modelling the variability at this most basic level is the most one can reduce their uncertainty in this environment. In the water environment, given a good model, one could predict which direction the variability would be confined to, essentially halving the range of expected locations, and reducing the surprise associated with large displacements provided they fall within the expected direction. In this sense, the two environments pitted model complexity (modelling both the waves and the variability in the water environment) against irreducible uncertainty (the full range of variability experienced in the sand environment).

Our results suggest that participants did have a significant albeit slight preference for the sand environment, preferring reduced model complexity to the reducible uncertainty afforded by the water environment, for the purposes of the agency-focused foraging task. This was a modest preference, with a mean difference of only one percent across trials, but it was reliable across different ways of measuring preference, with an average of 2.5% more trials being classified on the whole as sand dominant. This also did seem to garner a quantifiable advantage for judging agency at the end of each trial, with participants spending longer in the sand environment when they were correct in this judgement, and participants reporting greater confidence in their judgement with more time spent in the sand environment. This supports the idea that participants can learn to use environments to their advantage in facilitating unequivocal judgements of agency. Future work could titrate the variability to push participants towards greater model complexity or greater irreducible uncertainty over multiple trials to establish individual participant thresholds that may be associated with participant differences such as autism traits or self-concept clarity. While we expected that higher autism traits would predict stronger preference for environments that require lower model complexity, the preference for sand was not dependent on AQ score. Both environments were very simple, and future experiments should consider naturalistic environments with more hidden causes to uncover possible effects of AQ on environment preference. The validation of this task in the online setting makes these kinds of manipulations particularly accessible.

The pattern of prediction error (bPE) around environment switches suggests that participants were using a switch in environment (in either direction) as an intentional response to bPE, as demonstrated by the permutation testing results. The ERbPE mixed model showed that even when controlling for speed, the other factors contributing to bPE magnitude also increased before participants switched environments, and decreased afterward – namely, environmental variability and square offset (when the selected square was not the correct one). This is compelling evidence that participants are using the act of switching environments as a response to increasing bPE.

In the Introduction, we noted that one could reimagine these questions of environment selection in the context of skill acquisition. That is, prediction error dynamics resulting from variable action-outcome contingencies in different environments may inform environment selection to support more effectively garnering pragmatic or epistemic rewards. It may be too that the specific pattern of prediction error observed around environment switches also holds when environment switching is used to support these kinds of skill building activities. This is because developing the skill of judging agency is a precondition of developing other skills. In real life, such judgements usually occur without conscious deliberation. However, in cases of high environmental uncertainty leading to ambiguous action-outcome mappings, making these judgements accurately is vital to achieve other ends. Further, in ecologically valid settings, agency over parts of the environment comes in degrees (as in the case of partial or joint control), and be assigned different levels of confidence, further complicating binary judgements. Judging agency accurately is necessary but not sufficient for other kinds of skilled performance in uncertain environments. If someone cannot accurately assign agency, then they would not be able to effectively develop skills in ambiguous contexts. In such a case, the participant would experience mounting prediction error related to both their inability to precisify action-outcome mappings and establish a sense of control, but also related to their inability to use the established sense of control to reliably achieve other goals. The environment selection dynamics explored here in support of agency inferences are also intimately related to environment selection to support skill acquisition, and further research should explore this connection further.

The way environment switching is used as a strategy in response to bPE was modulated by participants’ autism traits. Participants with high AQ appear to tolerate less prediction error before acting in response to it, a finding that is consistent with AQ modulation of the hypothesis switch ERbPE reported in Perrykkad et al. (2021). Since bPE does not account for improved prior expectations based on successful modelling of the environment, these results might also indicate that lower autistic traits scores are associated with better modelling of the environment, thus while having higher bPE at the time of switching environments, they cognitively experience similar quantities of prediction error to participants with higher autism traits when choosing to act. Future work using neural measures of prediction error may be able to tease this apart.

We acknowledge that the use of autism traits rather than a clinically diagnosed population places limits to the generalisability of this result. While overall the sample size in post-hoc analyses used to compare interactions with autism traits is low, the omnibus interactions were based on modelled trends in the full dataset of continuous AQ scores. Nevertheless, environmental uncertainty might be particularly relevant to agency-focused foraging for different levels of autistic traits and we do show interactions between environment selection and AQ. These are worth following up in future studies in diagnosed populations.

Methodologically, the results of this online study demonstrate the robustness of the squares task in answering fine-grained questions despite increased noise from button press mechanics, internet/computing instability, and even without the use of eye-tracking for hypothesis tracking (cf. Perrykkad et al., 2021; see ESM Sections 2 and 3).

In the broader perspective, revealing how active foraging for agency-related information contributes to environment preferences can help us better understand agents’ sometimes perplexing choices to forego environments where they could forage for traditional rewards or information about the world. This may also help understand the environments preferred in some mental and developmental conditions. In a more generic learning context, perhaps foraging for agency-related information occurs because it helps agents solve the credit assignment problem, familiar from reinforcement learning (Rothkopf & Ballard, 2010). By pre-emptively ensuring they have a model of what they can and cannot precisely control in a given environment, agents will have a better fix on which parts of their actions should be credited with obtaining them their rewards. Overall, such findings complement the existing picture of factors at play (Mehlhorn et al., 2015) in foraging decisions including the rate and variability of resource depletion, and other factors, such as individual and social factors.

## Supplementary Information

ESM 1 (DOCX 898 kb)

## Data Availability

The dataset used for statistical analysis is freely available on Figshare (10.26180/14826579). Timing records for the pilot data that helped determine exclusion criteria are available on Figshare also (10.26180/19252082).

## Code Availability

Analysis scripts developed for this project are freely available on Github (https://github.com/kperrykkad/beach-task/). The task presentation script is also freely available on Github (https://github.com/robin0025/beach_squares_Perrykkad).

## Declarations

The following declarations are available elsewhere in the text, but are repeated here as a summary.
