# Supplementary material for: Foraging for the self: Environment selection for agency inference
Source: Psychon Bull Rev. 2022 Oct 11;30(2):608–20. doi: 10.3758/s13423-022-02187-w (PMC10104918; doi:10.3758/s13423-022-02187-w)

# Foraging for the Self: Environment Selection for Agency Inference

Perrykkad, Robinson and Hohwy

# Supplementary Materials:

## General Demographic Information

| Demographic | Category | N | %  (n=84) |
| --- | --- | --- | --- |
| Gender | Male | 59 | 70.2% |
|  | Female | 24 | 28.6% |
|  | Other | 1 | 1.2% |
| Age | 18-24 | 34 | 40.5% |
|  | 25-31 | 20 | 23.8% |
|  | 32-38 | 22 | 26.2% |
|  | 39-45 | 6 | 7.1% |
|  | 46-50 | 2 | 2.4% |
| Country of Residence | USA | 41 | 48.8% |
|  | Poland | 16 | 19.0% |
|  | United Kingdom | 6 | 7.1% |
|  | Portugal | 6 | 7.1% |
|  | Spain | 3 | 3.6% |
|  | Canada | 2 | 2.4% |
|  | France | 2 | 2.4% |
|  | Israel | 2 | 2.4% |
|  | Greece | 1 | 1.2% |
|  | Hungary | 1 | 1.2% |
|  | Czech Republic | 1 | 1.2% |
|  | Austria | 1 | 1.2% |
|  | Estonia | 1 | 1.2% |
|  | Netherlands | 1 | 1.2% |
| First Language | English | 53 | 63.1% |
|  | Other – Fluent in English | 31 | 36.9% |
| Highest Completed Education | Less than Highschool | 2 | 2.4% |
|  | Highschool or equivalent including Vocational Training | 29 | 34.5% |
|  | Bachelors, Honours, Associate, or Professional Degree | 32 | 38.1% |
|  | Masters or Doctorate | 21 | 25.0% |
| Employment Status | Unemployed or Not Working | 9 | 10.7% |
|  | Student or Intern | 23 | 27.4% |
|  | Employed | 52 | 61.9% |

## Data Quality

*Methods.* We begin by quantifying the average quality of stimulus presentation across participants devices and internet connections. We report the mean number of total frames in which stimuli was updated across each trial (for an intended 450 in 15s). We quantify the number of haemorrhaged lags which are periods of time between stimuli presentation frames longer than the expected duration of five frames (167ms). We also report the number of waves in an average trial in the water environment, their mode duration and their temporal variability given by the standard deviation of wave duration.

*Results.* On average, each participant had 12.58 trials of the total 48 removed for poor quality (std: 6.99, range: 0-24). In the remaining trials, an average of 317.20 frames (std: 64.97, range: 225-449) out of the programmed 450 were successfully presented per trial, putting actual stimulus presentation at an average of 21 hz. These trials had an average of 4.39 haemorrhaged lags (std:4.48, range:0-35) for an average lag duration of 2.98s (std: 2.93, range: 0-18.36). Since trials with less than half of frames presented were designated bad, when lags happen towards the end of the trial, after the majority of frames have been successfully presented, they may increase the overall time of the trial before the program recognises the trial time has elapsed on the frame following the lag. These variations to stimulus presentation due to variable computing and internet set ups mean that participants were presented with an average of 20.59 waves per trial (std: 4.28, range: 14-29) in the water environment with a mode wave time of 478.2ms per trial (std:112, range: 193-1432; compare to programmed 500ms) and a standard deviation of wave time per trial of 329.22 (std: 234.19, range: 7.16-2272).

## Comparison with Perrykkad et. al (2021): Validation of the Transition to Online

*Methods.* In order to validate the changes made due to practical considerations of putting the squares task online, we began with analyses which compare results from Perrykkad et al. (2021) with the experiment reported here. These comparisons primarily revolve around the use of button presses and reduced speed of selected object for moment-to-moment hypothesis selection rather than eye-position as in the previous version. To compare the studies directly, we compared how the percent of time participants spent with the correct square selected and the chosen square selected, split by correct and incorrect trials. The values for correct and chosen were equivalent for correct trials. In the current study, this percentage was of the total time spent with any square selected (using the button mechanic). In the previous study, these values are a percent of the total time in the trial, since a ‘hypothesis’ was always selected due to the eye-tracking methodology. To quantify any differences observed, a mixed model was used with the fixed factors of Study, Selected Square (chosen/correct) and Accuracy. We were primarily looking for interactions between study and the other factors as an indication of the magnitude of the impact of the change in mechanic to participant behaviour in completing the task. This mixed model did not control for stimulus presentation variability or include AQ as the other models did.

As in Perrykkad et al. (2021), a behavioural proxy for *prediction error* was calculated by taking the Euclidean distance between where the selected square would have ended up if it had followed the trajectory of the mouse input (with a constant speed multiplier as described above) and where the square actually went. This means that prediction error is largely under the control of the participants, and is influenced by three factors: 1) the speed of movement (determining the distance travelled, longer distances mean more prediction error) and the angular changes to the input determined by 2) the uncertainty in the environment and 3) the angular offset if the selected square is not the correct one.

Based on this prediction error measure, as part of the validation analyses, we computed a hypothesis switch centered ERPE for each participant using the same method as Perrykkad et al. (2021). To get roughly the same temporal epoch, we took 15 frames either side of the event (30hz presentation, ±500ms). This was uncorrected for temporal variability in stimulus presentation, so are more appropriately conceptualised as presented data points than time before and after the event of interest. In this version of the experiment, hypothesis switches require changing button presses, they are often surrounded by periods of inactivity. To account for this, in this experiment we separate all ERPEs into *lead-in* and *lead-out* epochs. In these epochs, if event is immediately preceded or followed by periods where nothing is selected, the ERPE epoch trigger is moved to the first (lead-out) or last (lead-in) time a hypothesis was validly selected, effectively removing periods of inactivity around the event of interest. Upon visual inspection of the hypothesis switch ERPE with the intended epoch, the lead-in was not at stable baseline levels at the start of a 15 frame epoch, so the lead-in epoch for this analysis was doubled (-30:15 frames around the event, centered at 0).

For statistical analysis, averages were taken in time bins of 5 frames during the epoch. For this analysis, there were nine time bins – six lead-in and three lead-out, with the time of the event occurring between or in time bins six and seven. The hypothesis switch ERPE was analysed using a mixed model with the fixed factor of time bin. The standard fixed effects of SCCS and autism traits; standard control fixed effects of number of frames and wave time variability; and standard random effect of participant were also included. All interactions between non-control fixed effects were included in this model. Main effects and interactions with time bin would indicate a pattern of prediction error around hypothesis switches, which is expected to peak at the time of the event, as in Perrykkad et al. (2021).

Prediction error slopes across various conditions could not be estimated as planned for most participants in this dataset due to the inconsistent nature of movement. Planned contrasts included slope by environment, and accuracy by agency as in Perrykkad et al. (2021). Slopes were successfully fitted to prediction error in both environments for only 21% of participants and across all four accuracy by agency conditions for only 5% of participants. Where they could be estimated at all, data on which the slopes were estimated was often very noisy. As such, no estimates of slope are reported.

*Results.* The mixed model used to validate the hypothesis switch mechanic by relating accurate responses and dwell time spent on relevant squares showed significant main effects of Accuracy and Selected Square but no significant main effect of Study. Further, interactions between Accuracy and Selected Square, Accuracy and Study, Selected Square and Study and the three-way interaction were significant. See Figure 2. As the primary question of interest here concerned differences between studies, only the interactions with study were followed up with post-hoc analyses. Post-hoc analyses showed that despite the significant interaction, when Selected Square was held constant, there was no difference between the studies (Correct: t(481.2) = 2.447, p = 0.089; Chosen: t(481.2) = -0.70, p = 1.00). For the interaction between Accuracy and Study, for incorrect trials there is no difference between studies (t(481.6) = -1.66, p = 0.58) but for correct trials, participants in Perrykkad et al. (2021) looked at the square they chose on average 5.73% of the trial longer than in the current sample (t(481.2) = 3.41, p = 0.004). For the three way interaction, the only time the two studies were significantly different across the four combinations of accuracy and selected square was the percentage of time spent with the chosen square selected in incorrect trials, in which the current study showed an increase of 8.09% over the dataset from Perrykkad et al. (2021) (t(467.5) = -8.09, p = 0.006). Overall, even when there was a significant difference between the dwell times on relevant trials across these two studies it was quite small (<10%). We take this to indicate that the manual button press hypothesis selection method was comparable to the previous eye-tracking hypothesis selection method used in Perrykkad et al. (2021).


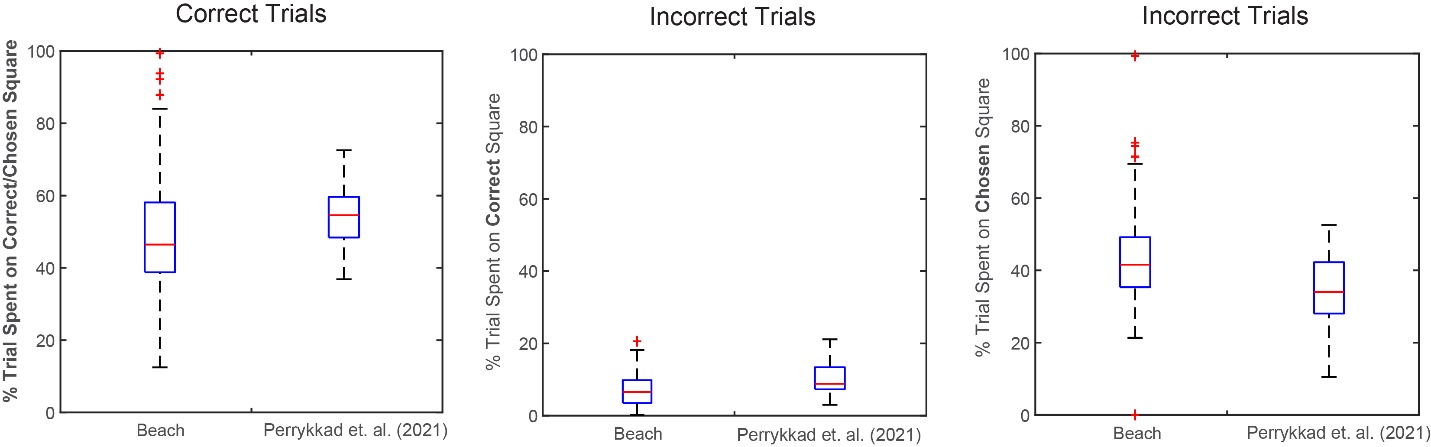


Figure 2 – Boxplot Comparison of Hypothesis Selection Mechanisms Current Study (Button Press) and Perrykkad et. al. (2021) (Eye-tracking). Y-axis indicates the percent of time spent on the relevant square. The left panel shows dwell time for correct trials, in which correct=chosen square, the right two panels are restricted to incorrect trials. The.middle panel represents correct square dwell time and right represents chosen square dwell time. Original finding from Perrykkad et al. (2021) is depicted in the right boxplot of each figure, and current study on the left for comparison.

The next validation step was to look at the hypothesis switch ERPE, in which Perrykkad et al. (2021) showed a clear peak at the time of the event. In this analysis, time bins 1-6 relate to five frame windows preceding hypothesis switches, and time bins 7-9 represent the lead-out of the hypothesis switch. Time bins six and seven either include the hypothesis switch, or the hypothesis switch happens between the two (when movement is ceased while switching hypothesis). See Figure 3. Results of the mixed model analysis show only a main effect of Time Bin (F(8,620.10) = 4.60, p = 0.000018). Post-hoc comparisons showed the average prediction error in time bins three, four and five are significantly greater than time bin seven (three: t(620.1) = 3.24, p = 0.045; four: t(620.0) = 4.41, p = 0.00044; five: t(620.0) = 4.88, p = 0.000049) and that time bin five is also greater than time bin nine (t(620.0) = 3.48, p = 0.019). This suggests that while there is some increase before the time of a hypothesis switch (time bins 3-5), and potentially a decrease after (time bin seven), there is not such a clear cut peak around the hypothesis switch as in Perrykkad et al. (2021). This can likely be attributed to slowing down in anticipation of manually changing a button press (eg. time bin six), and a generally noisier signal due to many fewer hypothesis switches per trial (mean = 3.0, SD = 2.31; in contrast to mean = 42.2, SD = 13.48).


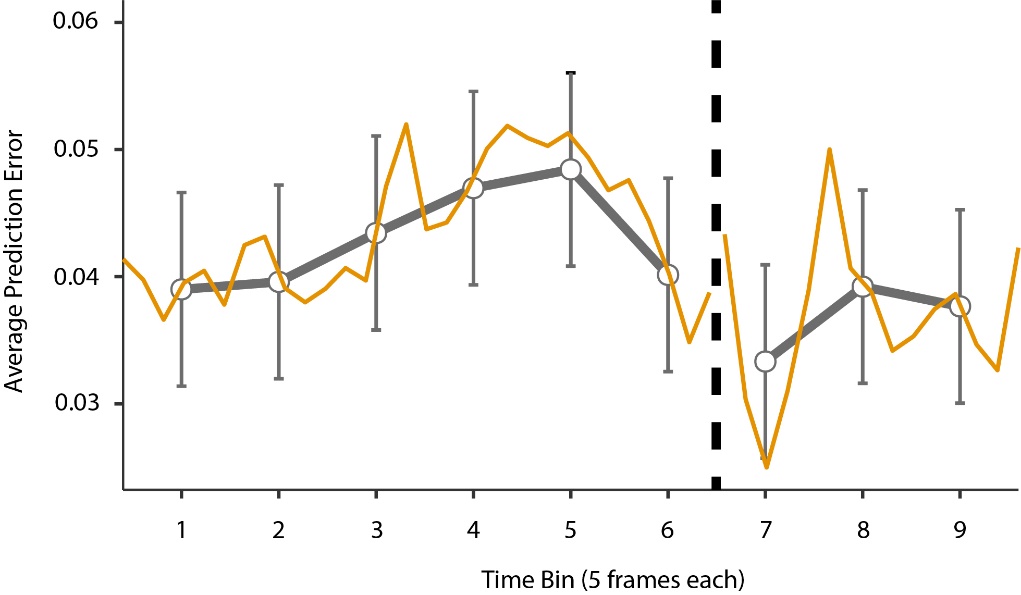


Figure 3 - Hypothesis Switch ERPE. Error bars are 95% CI. Y-axis shows average prediction error. The dotted line represents the time of a hypothesis switch from one square to another. The orange line depicts the grand average prediction error during the epoch, grey bars represent the average in each time bin which are used for statistical analysis.

## Control ERbPE Dataset Details

The control pattern was calculated by generating faux boundaries in the region between the edge of the screen and the center of the screen (true boundaries) with a spatial buffer on either side of these boundaries (equivalent to the average distance travelled in 5 frames). Faux boundaries also had to be unique (the same frame could not be selected in the same trial twice), could not coincide with hypothesis switches and could not temporally occur within 5 frames of a true environment switch. In each trial, one random faux boundary was generated for each true switch, except where there was no eligible boundary remaining, leading to a total of 18,090 faux events, compared to 20,877 true events across all participants and all trials (a difference of 13%).

Histogram of faux event locations – x-axis represents x locations on the screen (split into 100 bins), empty spaces are due to spatial buffer on eligibility from real boundaries (0,±0.88). y-axis represents count of included faux boundaries at each location across all trials and all participants. Inequalities are likely due to inconsistencies in the relationship between eligibility criteria and selected locations in time. Repeating faux event selection resulted in very similar looking histograms.

## Environment Switch ERPE Time Window Definition Analysis – Corrected for Speed

Note: dMouse is speed variable, realvfaux value of 1 = real, 2 = faux

Mixed Model

| Model Info | | | |
| --- | --- | --- | --- |
|  |  |  |  |
| **Info** | |  | |
| Estimate |  | Linear mixed model fit by REML |  |
| Call |  | avPE ~ 1 + timebin + realvfaux + dMouse + timebin:realvfaux+( 1 \| anonymousId ) |  |
| AIC |  | -24385.44100 |  |
| BIC |  | -23363.19430 |  |
| LogLikel. |  | 11951.95513 |  |
| R-squared Marginal |  | 0.46124 |  |
| R-squared Conditional |  | 0.67381 |  |
| Converged |  | yes |  |
| Optimizer |  | bobyqa |  |

Model Results

| Fixed Effect Omnibus tests | | | | | | | | | | | | | | | | | | | | | |  |  |  |  |  |  |
| --- | --- | --- | --- | --- | --- | --- | --- | --- | --- | --- | --- | --- | --- | --- | --- | --- | --- | --- | --- | --- | --- | --- | --- | --- | --- | --- | --- |
|  | | |  | |  | | |  | |  |  | |  | |  | |  | | |  | |  |  |  |  |  |  |
|  | | | | | **F** | | | | | **Num df** | | | **Den df** | | | | **p** | | | | |  |  |  |  |  |  |
| timebin | | |  | | 35.2339 | | |  | | 29 |  | | 5189.2 | |  | | 4.3552e-178 | | |  | |  |  |  |  |  |  |
| realvfaux | | |  | | 144.1676 | | |  | | 1 |  | | 5247.7 | |  | | 8.7523e0-33 | | |  | |  |  |  |  |  |  |
| dMouse | | |  | | 1108.3933 | | |  | | 1 |  | | 3909.9 | |  | | 3.4301e-214 | | |  | |  |  |  |  |  |  |
| timebin ✻ realvfaux | | |  | | 8.2053 | | |  | | 29 |  | | 5188.3 | |  | | 8.6956e0-34 | | |  | |  |  |  |  |  |  |
| Note. Satterthwaite method for degrees of freedom | | | | | | | | | | | | | | | | | | | | | |  |  |  |  |  |  |
|  | | | | | | | | | | | | | | | | | | | | | |  |  |  |  |  |  |
|  | | | | | | | | | | | | | | **95% Confidence Interval** | | | | | | |  | | | | | | |
| **Names** | | **Effect** | | | | **Estimate** | | | **SE** | | | | | **Lower** | | | | **Upper** | | | **df** | | | **t** | | **p** | |
| (Intercept) |  | (Intercept) | |  | | 0.0528435 |  | | 0.0020596 | | |  | | 0.0488068 | |  | | 0.0568802 |  | | 85.422 | |  | 25.657564 |  | 6.5890e0-42 |  |
| timebin1 |  | 2 - 1 | |  | | 0.0030915 |  | | 0.0025193 | | |  | | -0.0018462 | |  | | 0.0080292 |  | | 5187.546 | |  | 1.227122 |  | 0.2198324 |  |
| timebin2 |  | 3 - 1 | |  | | 0.0031040 |  | | 0.0025194 | | |  | | -0.0018339 | |  | | 0.0080419 |  | | 5187.631 | |  | 1.232039 |  | 0.2179902 |  |
| timebin3 |  | 4 - 1 | |  | | 0.0070143 |  | | 0.0025193 | | |  | | 0.0020765 | |  | | 0.0119521 |  | | 5187.589 | |  | 2.784178 |  | 0.0053858 |  |
| timebin4 |  | 5 - 1 | |  | | 0.0057751 |  | | 0.0025194 | | |  | | 8.3711e-4 | |  | | 0.0107131 |  | | 5187.665 | |  | 2.292225 |  | 0.0219325 |  |
| timebin5 |  | 6 - 1 | |  | | 0.0094188 |  | | 0.0025195 | | |  | | 0.0044806 | |  | | 0.0143570 |  | | 5187.744 | |  | 3.738295 |  | 1.8727e00-4 |  |
| timebin6 |  | 7 - 1 | |  | | 0.0067117 |  | | 0.0025197 | | |  | | 0.0017732 | |  | | 0.0116502 |  | | 5187.845 | |  | 2.663717 |  | 0.0077522 |  |
| timebin7 |  | 8 - 1 | |  | | 0.0090299 |  | | 0.0025194 | | |  | | 0.0040920 | |  | | 0.0139679 |  | | 5187.640 | |  | 3.584155 |  | 3.4128e00-4 |  |
| timebin8 |  | 9 - 1 | |  | | 0.0072383 |  | | 0.0025194 | | |  | | 0.0023004 | |  | | 0.0121762 |  | | 5187.625 | |  | 2.873031 |  | 0.0040820 |  |
| timebin9 |  | 10 - 1 | |  | | 0.0080723 |  | | 0.0025194 | | |  | | 0.0031344 | |  | | 0.0130103 |  | | 5187.631 | |  | 3.204072 |  | 0.0013632 |  |
| timebin10 |  | 11 - 1 | |  | | 0.0081369 |  | | 0.0025195 | | |  | | 0.0031987 | |  | | 0.0130752 |  | | 5187.744 | |  | 3.229532 |  | 0.0012477 |  |
| timebin11 |  | 12 - 1 | |  | | 0.0105252 |  | | 0.0025195 | | |  | | 0.0055870 | |  | | 0.0154633 |  | | 5187.729 | |  | 4.177439 |  | 2.9965e00-5 |  |
| timebin12 |  | 13 - 1 | |  | | 0.0170780 |  | | 0.0025193 | | |  | | 0.0121402 | |  | | 0.0220157 |  | | 5187.553 | |  | 6.778867 |  | 1.3460e0-11 |  |
| timebin13 |  | 14 - 1 | |  | | 0.0232865 |  | | 0.0025201 | | |  | | 0.0183472 | |  | | 0.0282259 |  | | 5188.204 | |  | 9.240177 |  | 3.5117e0-20 |  |
| timebin14 |  | 15 - 1 | |  | | 0.0436156 |  | | 0.0025329 | | |  | | 0.0386512 | |  | | 0.0485799 |  | | 5197.697 | |  | 17.219677 |  | 1.1434e0-64 |  |
| timebin15 |  | 16 - 1 | |  | | 0.0473578 |  | | 0.0025375 | | |  | | 0.0423845 | |  | | 0.0523312 |  | | 5200.943 | |  | 18.663478 |  | 2.6980e0-75 |  |
| timebin16 |  | 17 - 1 | |  | | 0.0193114 |  | | 0.0025194 | | |  | | 0.0143735 | |  | | 0.0242493 |  | | 5187.614 | |  | 7.665165 |  | 2.1185e0-14 |  |
| timebin17 |  | 18 - 1 | |  | | 0.0085352 |  | | 0.0025236 | | |  | | 0.0035890 | |  | | 0.0134813 |  | | 5190.829 | |  | 3.382165 |  | 7.2449e00-4 |  |
| timebin18 |  | 19 - 1 | |  | | 0.0046055 |  | | 0.0025207 | | |  | | -3.3506e−4 | |  | | 0.0095460 |  | | 5188.660 | |  | 1.827041 |  | 0.0677510 |  |
| timebin19 |  | 20 - 1 | |  | | 0.0064447 |  | | 0.0025198 | | |  | | 0.0015061 | |  | | 0.0113834 |  | | 5187.911 | |  | 2.557682 |  | 0.0105654 |  |
| timebin20 |  | 21 - 1 | |  | | 0.0087513 |  | | 0.0025194 | | |  | | 0.0038133 | |  | | 0.0136893 |  | | 5187.664 | |  | 3.473505 |  | 5.1791e00-4 |  |
| timebin21 |  | 22 - 1 | |  | | 0.0050050 |  | | 0.0025194 | | |  | | 6.7011e-5 | |  | | 0.0099430 |  | | 5187.660 | |  | 1.986562 |  | 0.0470235 |  |
| timebin22 |  | 23 - 1 | |  | | 0.0047089 |  | | 0.0025196 | | |  | | -2.2931e−4 | |  | | 0.0096472 |  | | 5187.760 | |  | 1.868954 |  | 0.0616855 |  |
| timebin23 |  | 24 - 1 | |  | | 0.0035683 |  | | 0.0025193 | | |  | | -0.0013693 | |  | | 0.0085060 |  | | 5187.534 | |  | 1.416421 |  | 0.1567123 |  |
| timebin24 |  | 25 - 1 | |  | | 0.0059077 |  | | 0.0025193 | | |  | | 9.6988e-4 | |  | | 0.0108455 |  | | 5187.576 | |  | 2.344938 |  | 0.0190677 |  |
| timebin25 |  | 26 - 1 | |  | | 0.0036745 |  | | 0.0025193 | | |  | | -0.0012631 | |  | | 0.0086122 |  | | 5187.534 | |  | 1.458570 |  | 0.1447440 |  |
| timebin26 |  | 27 - 1 | |  | | 0.0032659 |  | | 0.0025193 | | |  | | -0.0016719 | |  | | 0.0082037 |  | | 5187.595 | |  | 1.296322 |  | 0.1949224 |  |
| timebin27 |  | 28 - 1 | |  | | 0.0056398 |  | | 0.0025193 | | |  | | 7.0208e-4 | |  | | 0.0105774 |  | | 5187.534 | |  | 2.238648 |  | 0.0252210 |  |
| timebin28 |  | 29 - 1 | |  | | 0.0055015 |  | | 0.0025229 | | |  | | 5.5671e-4 | |  | | 0.0104464 |  | | 5187.560 | |  | 2.180623 |  | 0.0292561 |  |
| timebin29 |  | 30 - 1 | |  | | 0.0080068 |  | | 0.0025193 | | |  | | 0.0030691 | |  | | 0.0129445 |  | | 5187.548 | |  | 3.178190 |  | 0.0014907 |  |
| realvfaux1 |  | 2 - 1 | |  | | -0.0081326 |  | | 6.7733e-4 | | |  | | -0.0094602 | |  | | -0.0068051 |  | | 5247.747 | |  | -12.006980 |  | 8.7523e0-33 |  |
| dMouse |  | dMouse | |  | | 2.8182575 |  | | 0.0846513 | | |  | | 2.6523439 | |  | | 2.9841710 |  | | 3909.943 | |  | 33.292542 |  | 3.4301e-214 |  |
| timebin1 ✻ realvfaux1 |  | 2 - 1 ✻ 2 - 1 | |  | | 0.0016391 |  | | 0.0050385 | | |  | | -0.0082362 | |  | | 0.0115145 |  | | 5187.536 | |  | 0.325318 |  | 0.7449538 |  |
| timebin2 ✻ realvfaux1 |  | 3 - 1 ✻ 2 - 1 | |  | | 0.0014876 |  | | 0.0050386 | | |  | | -0.0083879 | |  | | 0.0113631 |  | | 5187.555 | |  | 0.295244 |  | 0.7678197 |  |
| timebin3 ✻ realvfaux1 |  | 4 - 1 ✻ 2 - 1 | |  | | 0.0021454 |  | | 0.0050385 | | |  | | -0.0077300 | |  | | 0.0120207 |  | | 5187.535 | |  | 0.425791 |  | 0.6702779 |  |
| timebin4 ✻ realvfaux1 |  | 5 - 1 ✻ 2 - 1 | |  | | 0.0030406 |  | | 0.0050385 | | |  | | -0.0068348 | |  | | 0.0129160 |  | | 5187.534 | |  | 0.603470 |  | 0.5462222 |  |
| timebin5 ✻ realvfaux1 |  | 6 - 1 ✻ 2 - 1 | |  | | 5.8362e-4 |  | | 0.0050385 | | |  | | -0.0092917 | |  | | 0.0104590 |  | | 5187.535 | |  | 0.115831 |  | 0.9077907 |  |
| timebin6 ✻ realvfaux1 |  | 7 - 1 ✻ 2 - 1 | |  | | 0.0015861 |  | | 0.0050386 | | |  | | -0.0082893 | |  | | 0.0114615 |  | | 5187.548 | |  | 0.314789 |  | 0.7529349 |  |
| timebin7 ✻ realvfaux1 |  | 8 - 1 ✻ 2 - 1 | |  | | -0.0014790 |  | | 0.0050385 | | |  | | -0.0113543 | |  | | 0.0083964 |  | | 5187.536 | |  | -0.293528 |  | 0.7691306 |  |
| timebin8 ✻ realvfaux1 |  | 9 - 1 ✻ 2 - 1 | |  | | -0.0038078 |  | | 0.0050386 | | |  | | -0.0136833 | |  | | 0.0060676 |  | | 5187.556 | |  | -0.755732 |  | 0.4498439 |  |
| timebin9 ✻ realvfaux1 |  | 10 - 1 ✻ 2 - 1 | |  | | -0.0108193 |  | | 0.0050386 | | |  | | -0.0206948 | |  | | -9.4371e−4 |  | | 5187.575 | |  | -2.147258 |  | 0.0318190 |  |
| timebin10 ✻ realvfaux1 |  | 11 - 1 ✻ 2 - 1 | |  | | -0.0132377 |  | | 0.0050389 | | |  | | -0.0231137 | |  | | -0.0033616 |  | | 5187.674 | |  | -2.627093 |  | 0.0086371 |  |
| timebin11 ✻ realvfaux1 |  | 12 - 1 ✻ 2 - 1 | |  | | -0.0121962 |  | | 0.0050390 | | |  | | -0.0220725 | |  | | -0.0023199 |  | | 5187.718 | |  | -2.420356 |  | 0.0155395 |  |
| timebin12 ✻ realvfaux1 |  | 13 - 1 ✻ 2 - 1 | |  | | -0.0150211 |  | | 0.0050399 | | |  | | -0.0248991 | |  | | -0.0051430 |  | | 5188.055 | |  | -2.980432 |  | 0.0028918 |  |
| timebin13 ✻ realvfaux1 |  | 14 - 1 ✻ 2 - 1 | |  | | -0.0191689 |  | | 0.0050419 | | |  | | -0.0290508 | |  | | -0.0092869 |  | | 5188.825 | |  | -3.801919 |  | 1.4523e00-4 |  |
| timebin14 ✻ realvfaux1 |  | 15 - 1 ✻ 2 - 1 | |  | | -0.0360094 |  | | 0.0050489 | | |  | | -0.0459051 | |  | | -0.0261138 |  | | 5191.484 | |  | -7.132141 |  | 1.1238e0-12 |  |
| timebin15 ✻ realvfaux1 |  | 16 - 1 ✻ 2 - 1 | |  | | -0.0384924 |  | | 0.0050491 | | |  | | -0.0483885 | |  | | -0.0285964 |  | | 5191.566 | |  | -7.623603 |  | 2.9152e0-14 |  |
| timebin16 ✻ realvfaux1 |  | 17 - 1 ✻ 2 - 1 | |  | | -0.0164671 |  | | 0.0050415 | | |  | | -0.0263483 | |  | | -0.0065859 |  | | 5188.683 | |  | -3.266299 |  | 0.0010967 |  |
| timebin17 ✻ realvfaux1 |  | 18 - 1 ✻ 2 - 1 | |  | | -0.0159360 |  | | 0.0050401 | | |  | | -0.0258144 | |  | | -0.0060576 |  | | 5188.133 | |  | -3.161847 |  | 0.0015768 |  |
| timebin18 ✻ realvfaux1 |  | 19 - 1 ✻ 2 - 1 | |  | | -0.0108984 |  | | 0.0050395 | | |  | | -0.0207756 | |  | | -0.0010212 |  | | 5187.890 | |  | -2.162613 |  | 0.0306165 |  |
| timebin19 ✻ realvfaux1 |  | 20 - 1 ✻ 2 - 1 | |  | | -0.0093730 |  | | 0.0050386 | | |  | | -0.0192485 | |  | | 5.0258e-4 |  | | 5187.574 | |  | -1.860219 |  | 0.0629111 |  |
| timebin20 ✻ realvfaux1 |  | 21 - 1 ✻ 2 - 1 | |  | | -0.0073380 |  | | 0.0050386 | | |  | | -0.0172134 | |  | | 0.0025375 |  | | 5187.546 | |  | -1.456358 |  | 0.1453542 |  |
| timebin21 ✻ realvfaux1 |  | 22 - 1 ✻ 2 - 1 | |  | | -0.0049368 |  | | 0.0050386 | | |  | | -0.0148123 | |  | | 0.0049386 |  | | 5187.553 | |  | -0.979807 |  | 0.3272272 |  |
| timebin22 ✻ realvfaux1 |  | 23 - 1 ✻ 2 - 1 | |  | | -0.0041165 |  | | 0.0050388 | | |  | | -0.0139924 | |  | | 0.0057593 |  | | 5187.623 | |  | -0.816972 |  | 0.4139821 |  |
| timebin23 ✻ realvfaux1 |  | 24 - 1 ✻ 2 - 1 | |  | | 1.2102e-4 |  | | 0.0050393 | | |  | | -0.0097559 | |  | | 0.0099979 |  | | 5187.837 | |  | 0.024016 |  | 0.9808408 |  |
| timebin24 ✻ realvfaux1 |  | 25 - 1 ✻ 2 - 1 | |  | | -0.0051504 |  | | 0.0050386 | | |  | | -0.0150260 | |  | | 0.0047252 |  | | 5187.574 | |  | -1.022179 |  | 0.3067437 |  |
| timebin25 ✻ realvfaux1 |  | 26 - 1 ✻ 2 - 1 | |  | | 5.4888e-4 |  | | 0.0050386 | | |  | | -0.0093265 | |  | | 0.0104243 |  | | 5187.541 | |  | 0.108936 |  | 0.9132576 |  |
| timebin26 ✻ realvfaux1 |  | 27 - 1 ✻ 2 - 1 | |  | | -0.0022981 |  | | 0.0050386 | | |  | | -0.0121735 | |  | | 0.0075774 |  | | 5187.553 | |  | -0.456096 |  | 0.6483399 |  |
| timebin27 ✻ realvfaux1 |  | 28 - 1 ✻ 2 - 1 | |  | | -0.0033944 |  | | 0.0050394 | | |  | | -0.0132713 | |  | | 0.0064826 |  | | 5187.848 | |  | -0.673570 |  | 0.5006145 |  |
| timebin28 ✻ realvfaux1 |  | 29 - 1 ✻ 2 - 1 | |  | | -0.0036062 |  | | 0.0050465 | | |  | | -0.0134973 | |  | | 0.0062848 |  | | 5187.816 | |  | -0.714599 |  | 0.4748892 |  |
| timebin29 ✻ realvfaux1 |  | 30 - 1 ✻ 2 - 1 | |  | | 9.0452e-5 |  | | 0.0050396 | | |  | | -0.0097869 | |  | | 0.0099678 |  | | 5187.929 | |  | 0.017948 |  | 0.9856807 |  |
|  | | | | | | | | | | | | | | | | | | | | | | | | | | | |

| Random Components | | | | | | | | | |
| --- | --- | --- | --- | --- | --- | --- | --- | --- | --- |
|  |  |  |  |  |  |  |  |  |  |
| **Groups** | | **Name** | | **SD** | | **Variance** | | **ICC** | |
| anonymousId |  | (Intercept) |  | 0.019186 |  | 3.6811e-4 |  | 0.39456 |  |
| Residual |  |  |  | 0.023767 |  | 5.6486e-4 |  |  |  |
| Note. Number of Obs: 5339 , groups: anonymousId 89 | | | | | | | | | |

Simple Effects

| Simple effects of realvfaux : Omnibus Tests | | | | | | | | | |
| --- | --- | --- | --- | --- | --- | --- | --- | --- | --- |
| **Moderator levels** | |  | | | | | | | |
| **timebin** | | **F** | | **Num df** | | **Den df** | | **p** | |
| 1 |  | 0.0400000 |  | 1.0000 |  | 5189.4 |  | 0.8409207 |  |
| 2 |  | 0.0670000 |  | 1.0000 |  | 5189.3 |  | 0.7957122 |  |
| 3 |  | 0.0470000 |  | 1.0000 |  | 5190.0 |  | 0.8287322 |  |
| 4 |  | 0.1610000 |  | 1.0000 |  | 5189.6 |  | 0.6885729 |  |
| 5 |  | 0.4250000 |  | 1.0000 |  | 5189.3 |  | 0.5144895 |  |
| 6 |  | 0.0010000 |  | 1.0000 |  | 5189.6 |  | 0.9704277 |  |
| 7 |  | 0.0600000 |  | 1.0000 |  | 5189.0 |  | 0.8071830 |  |
| 8 |  | 0.3790000 |  | 1.0000 |  | 5189.3 |  | 0.5382616 |  |
| 9 |  | 1.6100000 |  | 1.0000 |  | 5188.9 |  | 0.2045671 |  |
| 10 |  | 10.4700000 |  | 1.0000 |  | 5188.7 |  | 0.0012212 |  |
| 11 |  | 15.2810000 |  | 1.0000 |  | 5191.1 |  | 9.3806e0-5 |  |
| 12 |  | 13.0810000 |  | 1.0000 |  | 5191.4 |  | 3.0109e0-4 |  |
| 13 |  | 19.3950000 |  | 1.0000 |  | 5193.2 |  | 1.0842e0-5 |  |
| 14 |  | 30.8660000 |  | 1.0000 |  | 5196.2 |  | 2.9020e0-8 |  |
| 15 |  | 104.3540000 |  | 1.0000 |  | 5204.2 |  | 2.8642e-24 |  |
| 16 |  | 118.9110000 |  | 1.0000 |  | 5204.4 |  | 2.1624e-27 |  |
| 17 |  | 23.0610000 |  | 1.0000 |  | 5195.7 |  | 1.6134e0-6 |  |
| 18 |  | 21.7080000 |  | 1.0000 |  | 5193.5 |  | 3.2542e0-6 |  |
| 19 |  | 10.5730000 |  | 1.0000 |  | 5192.4 |  | 0.0011548 |  |
| 20 |  | 7.9960000 |  | 1.0000 |  | 5190.3 |  | 0.0047062 |  |
| 21 |  | 5.1020000 |  | 1.0000 |  | 5189.0 |  | 0.0239373 |  |
| 22 |  | 2.5140000 |  | 1.0000 |  | 5188.9 |  | 0.1129253 |  |
| 23 |  | 1.8380000 |  | 1.0000 |  | 5188.4 |  | 0.1752509 |  |
| 24 |  | 0.0280000 |  | 1.0000 |  | 5187.9 |  | 0.8674375 |  |
| 25 |  | 2.7080000 |  | 1.0000 |  | 5188.7 |  | 0.0999250 |  |
| 26 |  | 0.0020000 |  | 1.0000 |  | 5189.1 |  | 0.9626542 |  |
| 27 |  | 0.7150000 |  | 1.0000 |  | 5188.9 |  | 0.3979637 |  |
| 28 |  | 1.3300000 |  | 1.0000 |  | 5187.9 |  | 0.2487817 |  |
| 29 |  | 1.4630000 |  | 1.0000 |  | 5188.0 |  | 0.2265717 |  |
| 30 |  | 0.0310000 |  | 1.0000 |  | 5187.8 |  | 0.8606835 |  |
|  | | | | | | | | | |

| Simple effects of realvfaux : Parameter estimates | | | | | | | | | | | | | | | | | |
| --- | --- | --- | --- | --- | --- | --- | --- | --- | --- | --- | --- | --- | --- | --- | --- | --- | --- |
| **Moderator levels** | |  | | | | | | **95% Confidence Interval** | | | |  | | | | | |
| **timebin** | | **contrast** | | **Estimate** | | **SE** | | **Lower** | | **Upper** | | **df** | | **t** | | **P**  **(*=fdr corrected significant)** | |
| 1 |  | 2 - 1 |  | -7.1585e−4 |  | 0.0035663 |  | -0.0077073 |  | 0.0062756 |  | 5189.4 |  | -0.200726 |  | 0.8409207 |  |
| 2 |  | 2 - 1 |  | 9.2328e-4 |  | 0.0035660 |  | -0.0060676 |  | 0.0079141 |  | 5189.3 |  | 0.258914 |  | 0.7957122 |  |
| 3 |  | 2 - 1 |  | 7.7177e-4 |  | 0.0035674 |  | -0.0062219 |  | 0.0077654 |  | 5190.0 |  | 0.216339 |  | 0.8287322 |  |
| 4 |  | 2 - 1 |  | 0.0014295 |  | 0.0035665 |  | -0.0055624 |  | 0.0084214 |  | 5189.6 |  | 0.400815 |  | 0.6885729 |  |
| 5 |  | 2 - 1 |  | 0.0023248 |  | 0.0035661 |  | -0.0046663 |  | 0.0093158 |  | 5189.3 |  | 0.651908 |  | 0.5144895 |  |
| 6 |  | 2 - 1 |  | -1.3222e−4 |  | 0.0035665 |  | -0.0071241 |  | 0.0068597 |  | 5189.6 |  | -0.037074 |  | 0.9704277 |  |
| 7 |  | 2 - 1 |  | 8.7024e-4 |  | 0.0035655 |  | -0.0061196 |  | 0.0078601 |  | 5189.0 |  | 0.244074 |  | 0.8071830 |  |
| 8 |  | 2 - 1 |  | -0.0021948 |  | 0.0035660 |  | -0.0091856 |  | 0.0047960 |  | 5189.3 |  | -0.615485 |  | 0.5382616 |  |
| 9 |  | 2 - 1 |  | -0.0045237 |  | 0.0035653 |  | -0.0115132 |  | 0.0024658 |  | 5188.9 |  | -1.268806 |  | 0.2045671 |  |
| 10 |  | 2 - 1 |  | -0.0115351 |  | 0.0035650 |  | -0.0185240 |  | -0.0045462 |  | 5188.7 |  | -3.235672 |  | 0.0012212* |  |
| 11 |  | 2 - 1 |  | -0.0139535 |  | 0.0035695 |  | -0.0209512 |  | -0.0069559 |  | 5191.1 |  | -3.909139 |  | 9.3806e0-5* |  |
| 12 |  | 2 - 1 |  | -0.0129121 |  | 0.0035700 |  | -0.0199108 |  | -0.0059133 |  | 5191.4 |  | -3.616813 |  | 3.0109e0-4* |  |
| 13 |  | 2 - 1 |  | -0.0157369 |  | 0.0035733 |  | -0.0227421 |  | -0.0087317 |  | 5193.2 |  | -4.403978 |  | 1.0842e0-5* |  |
| 14 |  | 2 - 1 |  | -0.0198847 |  | 0.0035791 |  | -0.0269013 |  | -0.0128681 |  | 5196.2 |  | -5.555742 |  | 2.9020e0-8* |  |
| 15 |  | 2 - 1 |  | -0.0367253 |  | 0.0035951 |  | -0.0437732 |  | -0.0296774 |  | 5204.2 |  | -10.215364 |  | 2.8642e-24* |  |
| 16 |  | 2 - 1 |  | -0.0392083 |  | 0.0035956 |  | -0.0462571 |  | -0.0321595 |  | 5204.4 |  | -10.904646 |  | 2.1624e-27* |  |
| 17 |  | 2 - 1 |  | -0.0171830 |  | 0.0035781 |  | -0.0241977 |  | -0.0101683 |  | 5195.7 |  | -4.802198 |  | 1.6134e0-6* |  |
| 18 |  | 2 - 1 |  | -0.0166519 |  | 0.0035740 |  | -0.0236584 |  | -0.0096453 |  | 5193.5 |  | -4.659169 |  | 3.2542e0-6* |  |
| 19 |  | 2 - 1 |  | -0.0116143 |  | 0.0035718 |  | -0.0186166 |  | -0.0046119 |  | 5192.4 |  | -3.251614 |  | 0.00115489* |  |
| 20 |  | 2 - 1 |  | -0.0100888 |  | 0.0035678 |  | -0.0170833 |  | -0.0030943 |  | 5190.3 |  | -2.827707 |  | 0.0047062* |  |
| 21 |  | 2 - 1 |  | -0.0080538 |  | 0.0035655 |  | -0.0150438 |  | -0.0010639 |  | 5189.0 |  | -2.258799 |  | 0.0239373 |  |
| 22 |  | 2 - 1 |  | -0.0056527 |  | 0.0035654 |  | -0.0126423 |  | 0.0013369 |  | 5188.9 |  | -1.585449 |  | 0.1129253 |  |
| 23 |  | 2 - 1 |  | -0.0048324 |  | 0.0035645 |  | -0.0118202 |  | 0.0021555 |  | 5188.5 |  | -1.355709 |  | 0.1752509 |  |
| 24 |  | 2 - 1 |  | -5.9482e−4 |  | 0.0035635 |  | -0.0075807 |  | 0.0063910 |  | 5187.9 |  | -0.166923 |  | 0.8674375 |  |
| 25 |  | 2 - 1 |  | -0.0058662 |  | 0.0035650 |  | -0.0128551 |  | 0.0011227 |  | 5188.7 |  | -1.645511 |  | 0.0999250 |  |
| 26 |  | 2 - 1 |  | -1.6697e−4 |  | 0.0035657 |  | -0.0071572 |  | 0.0068233 |  | 5189.1 |  | -0.046825 |  | 0.9626542 |  |
| 27 |  | 2 - 1 |  | -0.0030139 |  | 0.0035654 |  | -0.0100036 |  | 0.0039757 |  | 5188.9 |  | -0.845333 |  | 0.3979637 |  |
| 28 |  | 2 - 1 |  | -0.0041102 |  | 0.0035634 |  | -0.0110960 |  | 0.0028756 |  | 5187.9 |  | -1.153443 |  | 0.2487817 |  |
| 29 |  | 2 - 1 |  | -0.0043221 |  | 0.0035738 |  | -0.0113282 |  | 0.0026841 |  | 5188.0 |  | -1.209381 |  | 0.2265717 |  |
| 30 |  | 2 - 1 |  | -6.2539e−4 |  | 0.0035632 |  | -0.0076108 |  | 0.0063600 |  | 5187.8 |  | -0.175513 |  | 0.8606835 |  |
| Note. Simple effects are estimated keeping constant other independent variable(s) in the model | | | | | | | | | | | | | | | | | |

Effects Plots


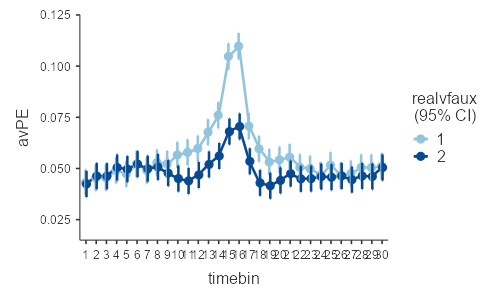


NB: 1 = Real 2 = Faux

## Environment Switch ERPE Uncorrected for Speed

Mixed Model

| Model Info | | | |
| --- | --- | --- | --- |
|  |  |  |  |
| **Info** | |  | |
| Estimate |  | Linear mixed model fit by REML |  |
| Call |  | avPE ~ 1 + AQ + SCC + directionToS1W2 + timebin + nFrames + sdWaveTime + directionToS1W2:timebin + directionToS1W2:AQ + timebin:AQ + directionToS1W2:SCC + timebin:SCC + timebin:directionToS1W2:AQ + timebin:directionToS1W2:SCC+( 1 \| anonymousId.qual ) |  |
| AIC |  | -9617.40570 |  |
| BIC |  | -8044.90110 |  |
| LogLikel. |  | 4364.02347 |  |
| R-squared Marginal |  | 0.19619 |  |
| R-squared Conditional |  | 0.79010 |  |
| Converged |  | yes |  |
| Optimizer |  | bobyqa |  |
|  | | | |

Model Results

| Fixed Effect Omnibus tests | | | | | | | | | |
| --- | --- | --- | --- | --- | --- | --- | --- | --- | --- |
|  |  |  |  |  |  |  |  |  |  |
|  | | **F** | | **Num df** | | **Den df** | | **p** | |
| AQ |  | 0.0022627 |  | 1 |  | 79.000 |  | 0.9621810 |  |
| SCC |  | 0.3981709 |  | 1 |  | 79.000 |  | 0.5298568 |  |
| directionToS1W2 |  | 0.1426797 |  | 1 |  | 2187.000 |  | 0.7056681 |  |
| timebin |  | 118.4361218 |  | 13 |  | 2187.000 |  | 1.0922e-241 |  |
| nFrames |  | 0.1053326 |  | 1 |  | 79.000 |  | 0.7463796 |  |
| sdWaveTime |  | 6.5359134 |  | 1 |  | 79.000 |  | 0.0124880 |  |
| directionToS1W2 ✻ timebin |  | 0.5419968 |  | 13 |  | 2187.000 |  | 0.8994358 |  |
| AQ ✻ directionToS1W2 |  | 0.7336715 |  | 1 |  | 2187.000 |  | 0.3917895 |  |
| AQ ✻ timebin |  | 2.4912772 |  | 13 |  | 2187.000 |  | 0.0022208 |  |
| SCC ✻ directionToS1W2 |  | 2.5059831 |  | 1 |  | 2187.000 |  | 0.1135593 |  |
| SCC ✻ timebin |  | 0.9115314 |  | 13 |  | 2187.000 |  | 0.5401777 |  |
| AQ ✻ directionToS1W2 ✻ timebin |  | 1.0082809 |  | 13 |  | 2187.000 |  | 0.4400134 |  |
| SCC ✻ directionToS1W2 ✻ timebin |  | 0.5629603 |  | 13 |  | 2187.000 |  | 0.8846564 |  |
| Note. Satterthwaite method for degrees of freedom | | | | | | | | | |
|  | | | | | | | | | |

| Fixed Effects Parameter Estimates | | | | | | | | | | | | | | | | | | | | | | | | | | | |
| --- | --- | --- | --- | --- | --- | --- | --- | --- | --- | --- | --- | --- | --- | --- | --- | --- | --- | --- | --- | --- | --- | --- | --- | --- | --- | --- | --- |
|  | | | | | | | | | | | | | | **95% Confidence Interval** | | | | | | | |  | | | | | |
| **Names** | | **Effect** | | | | **Estimate** | | | | **SE** | | | | **Lower** | | | | **Upper** | | | | **df** | | **t** | | **p** | |
| (Intercept) |  | (Intercept) | | |  | 0.0723833 | | |  | 0.0052495 | | |  | 0.0620945 | |  | | 0.0826722 | |  | | 79.000 |  | 13.788546 |  | 9.9577e0-23 |  |
| AQ |  | AQ | | |  | 3.8001e-5 | | |  | 7.9888e-4 | | |  | -0.0015278 | |  | | 0.0016038 | |  | | 79.000 |  | 0.047568 |  | 0.9621810 |  |
| SCC |  | SCC | | |  | -3.1522e−4 | | |  | 4.9956e-4 | | |  | -0.0012943 | |  | | 6.6389e-4 | |  | | 79.000 |  | -0.631008 |  | 0.5298568 |  |
| directionToS1W21 |  | 2 - 1 | | |  | -4.4276e−4 | | |  | 0.0011722 | | |  | -0.0027402 | |  | | 0.0018546 | |  | | 2187.000 |  | -0.377730 |  | 0.7056681 |  |
| timebin1 |  | 2 - 1 | | |  | 0.0027023 | | |  | 0.0031013 | | |  | -0.0033760 | |  | | 0.0087807 | |  | | 2187.000 |  | 0.871365 |  | 0.3836507 |  |
| timebin2 |  | 3 - 1 | | |  | 0.0045213 | | |  | 0.0031013 | | |  | -0.0015570 | |  | | 0.0105996 | |  | | 2187.000 |  | 1.457894 |  | 0.1450133 |  |
| timebin3 |  | 4 - 1 | | |  | 0.0152235 | | |  | 0.0031013 | | |  | 0.0091452 | |  | | 0.0213018 | |  | | 2187.000 |  | 4.908824 |  | 9.8394e00-7 |  |
| timebin4 |  | 5 - 1 | | |  | 0.0270108 | | |  | 0.0031013 | | |  | 0.0209325 | |  | | 0.0330891 | |  | | 2187.000 |  | 8.709642 |  | 5.8975e0-18 |  |
| timebin5 |  | 6 - 1 | | |  | 0.0641133 | | |  | 0.0031013 | | |  | 0.0580350 | |  | | 0.0701916 | |  | | 2187.000 |  | 20.673361 |  | 7.1575e0-87 |  |
| timebin6 |  | 7 - 1 | | |  | 0.0705855 | | |  | 0.0031013 | | |  | 0.0645071 | |  | | 0.0766638 | |  | | 2187.000 |  | 22.760317 |  | 4.3068e-103 |  |
| timebin7 |  | 8 - 1 | | |  | 0.0191775 | | |  | 0.0031013 | | |  | 0.0130992 | |  | | 0.0252559 | |  | | 2187.000 |  | 6.183806 |  | 7.4452e0-10 |  |
| timebin8 |  | 9 - 1 | | |  | 0.0116846 | | |  | 0.0031013 | | |  | 0.0056062 | |  | | 0.0177629 | |  | | 2187.000 |  | 3.767701 |  | 1.6911e00-4 |  |
| timebin9 |  | 10 - 1 | | |  | 0.0023059 | | |  | 0.0031013 | | |  | -0.0037724 | |  | | 0.0083843 | |  | | 2187.000 |  | 0.743546 |  | 0.4572313 |  |
| timebin10 |  | 11 - 1 | | |  | 0.0016591 | | |  | 0.0031013 | | |  | -0.0044193 | |  | | 0.0077374 | |  | | 2187.000 |  | 0.534963 |  | 0.5927301 |  |
| timebin11 |  | 12 - 1 | | |  | 8.9884e-4 | | |  | 0.0031013 | | |  | -0.0051795 | |  | | 0.0069772 | |  | | 2187.000 |  | 0.289833 |  | 0.7719716 |  |
| timebin12 |  | 13 - 1 | | |  | -0.0037744 | | |  | 0.0031013 | | |  | -0.0098527 | |  | | 0.0023040 | |  | | 2187.000 |  | -1.217051 |  | 0.2237162 |  |
| timebin13 |  | 14 - 1 | | |  | -0.0037984 | | |  | 0.0031013 | | |  | -0.0098767 | |  | | 0.0022800 | |  | | 2187.000 |  | -1.224790 |  | 0.2207864 |  |
| nFrames |  | nFrames | | |  | 4.1195e-5 | | |  | 1.2693e-4 | | |  | -2.0758e−4 | |  | | 2.8997e-4 | |  | | 79.000 |  | 0.324550 |  | 0.7463796 |  |
| sdWaveTime |  | sdWaveTime | | |  | 0.0924542 | | |  | 0.0361637 | | |  | 0.0215745 | |  | | 0.1633338 | |  | | 79.000 |  | 2.556543 |  | 0.0124880 |  |
| directionToS1W21 ✻ timebin1 |  | 2 - 1 ✻ 2 - 1 | | |  | -0.0041888 | | |  | 0.0062025 | | |  | -0.0163455 | |  | | 0.0079679 | |  | | 2187.000 |  | -0.675339 |  | 0.4995319 |  |
| directionToS1W21 ✻ timebin2 |  | 2 - 1 ✻ 3 - 1 | | |  | 0.0042900 | | |  | 0.0062025 | | |  | -0.0078666 | |  | | 0.0164467 | |  | | 2187.000 |  | 0.691663 |  | 0.4892223 |  |
| directionToS1W21 ✻ timebin3 |  | 2 - 1 ✻ 4 - 1 | | |  | 0.0016448 | | |  | 0.0062025 | | |  | -0.0105119 | |  | | 0.0138015 | |  | | 2187.000 |  | 0.265183 |  | 0.7908938 |  |
| directionToS1W21 ✻ timebin4 |  | 2 - 1 ✻ 5 - 1 | | |  | 0.0012632 | | |  | 0.0062025 | | |  | -0.0108935 | |  | | 0.0134198 | |  | | 2187.000 |  | 0.203653 |  | 0.8386438 |  |
| directionToS1W21 ✻ timebin5 |  | 2 - 1 ✻ 6 - 1 | | |  | -0.0052424 | | |  | 0.0062025 | | |  | -0.0173991 | |  | | 0.0069143 | |  | | 2187.000 |  | -0.845206 |  | 0.3980885 |  |
| directionToS1W21 ✻ timebin6 |  | 2 - 1 ✻ 7 - 1 | | |  | -0.0019920 | | |  | 0.0062025 | | |  | -0.0141486 | |  | | 0.0101647 | |  | | 2187.000 |  | -0.321153 |  | 0.7481251 |  |
| directionToS1W21 ✻ timebin7 |  | 2 - 1 ✻ 8 - 1 | | |  | 0.0021845 | | |  | 0.0062025 | | |  | -0.0099722 | |  | | 0.0143411 | |  | | 2187.000 |  | 0.352189 |  | 0.7247301 |  |
| directionToS1W21 ✻ timebin8 |  | 2 - 1 ✻ 9 - 1 | | |  | 0.0019763 | | |  | 0.0062025 | | |  | -0.0101804 | |  | | 0.0141330 | |  | | 2187.000 |  | 0.318628 |  | 0.7500389 |  |
| directionToS1W21 ✻ timebin9 |  | 2 - 1 ✻ 10 - 1 | | |  | -9.6412e−4 | | |  | 0.0062025 | | |  | -0.0131208 | |  | | 0.0111926 | |  | | 2187.000 |  | -0.155440 |  | 0.8764890 |  |
| directionToS1W21 ✻ timebin10 |  | 2 - 1 ✻ 11 - 1 | | |  | 0.0016010 | | |  | 0.0062025 | | |  | -0.0105557 | |  | | 0.0137577 | |  | | 2187.000 |  | 0.258123 |  | 0.7963362 |  |
| directionToS1W21 ✻ timebin11 |  | 2 - 1 ✻ 12 - 1 | | |  | -0.0060136 | | |  | 0.0062025 | | |  | -0.0181702 | |  | | 0.0061431 | |  | | 2187.000 |  | -0.969537 |  | 0.3323846 |  |
| directionToS1W21 ✻ timebin12 |  | 2 - 1 ✻ 13 - 1 | | |  | 6.6932e-4 | | |  | 0.0062025 | | |  | -0.0114874 | |  | | 0.0128260 | |  | | 2187.000 |  | 0.107911 |  | 0.9140764 |  |
| directionToS1W21 ✻ timebin13 |  | 2 - 1 ✻ 14 - 1 | | |  | -0.0046207 | | |  | 0.0062025 | | |  | -0.0167774 | |  | | 0.0075360 | |  | | 2187.000 |  | -0.744974 |  | 0.4563674 |  |
| AQ ✻ directionToS1W21 |  | AQ ✻ 2 - 1 | | |  | 1.4869e-4 | | |  | 1.7359e-4 | | |  | -1.9154e−4 | |  | | 4.8891e-4 | |  | | 2187.000 |  | 0.856546 |  | 0.3917895 |  |
| AQ ✻ timebin1 |  | AQ ✻ 2 - 1 | | |  | 2.3861e-4 | | |  | 4.5927e-4 | | |  | -6.6155e−4 | |  | | 0.0011388 | |  | | 2187.000 |  | 0.519541 |  | 0.6034364 |  |
| AQ ✻ timebin2 |  | AQ ✻ 3 - 1 | | |  | -1.3957e−4 | | |  | 4.5927e-4 | | |  | -0.0010397 | |  | | 7.6059e-4 | |  | | 2187.000 |  | -0.303883 |  | 0.7612462 |  |
| AQ ✻ timebin3 |  | AQ ✻ 4 - 1 | | |  | -1.3509e−4 | | |  | 4.5927e-4 | | |  | -0.0010352 | |  | | 7.6507e-4 | |  | | 2187.000 |  | -0.294135 |  | 0.7686824 |  |
| AQ ✻ timebin4 |  | AQ ✻ 5 - 1 | | |  | -6.8697e−4 | | |  | 4.5927e-4 | | |  | -0.0015871 | |  | | 2.1319e-4 | |  | | 2187.000 |  | -1.495769 |  | 0.1348582 |  |
| AQ ✻ timebin5 |  | AQ ✻ 6 - 1 | | |  | -0.0014502 | | |  | 4.5927e-4 | | |  | -0.0023504 | |  | | -5.5004e−4 | |  | | 2187.000 |  | -3.157598 |  | 0.0016124 |  |
| AQ ✻ timebin6 |  | AQ ✻ 7 - 1 | | |  | -0.0011274 | | |  | 4.5927e-4 | | |  | -0.0020275 | |  | | -2.2722e−4 | |  | | 2187.000 |  | -2.454692 |  | 0.0141779 |  |
| AQ ✻ timebin7 |  | AQ ✻ 8 - 1 | | |  | -7.5114e−4 | | |  | 4.5927e-4 | | |  | -0.0016513 | |  | | 1.4902e-4 | |  | | 2187.000 |  | -1.635498 |  | 0.1020885 |  |
| AQ ✻ timebin8 |  | AQ ✻ 9 - 1 | | |  | -4.0868e−4 | | |  | 4.5927e-4 | | |  | -0.0013088 | |  | | 4.9148e-4 | |  | | 2187.000 |  | -0.889846 |  | 0.3736462 |  |
| AQ ✻ timebin9 |  | AQ ✻ 10 - 1 | | |  | -2.5280e−4 | | |  | 4.5927e-4 | | |  | -0.0011530 | |  | | 6.4736e-4 | |  | | 2187.000 |  | -0.550431 |  | 0.5820798 |  |
| AQ ✻ timebin10 |  | AQ ✻ 11 - 1 | | |  | 3.1773e-5 | | |  | 4.5927e-4 | | |  | -8.6839e−4 | |  | | 9.3193e-4 | |  | | 2187.000 |  | 0.069180 |  | 0.9448525 |  |
| AQ ✻ timebin11 |  | AQ ✻ 12 - 1 | | |  | 1.7640e-4 | | |  | 4.5927e-4 | | |  | -7.2375e−4 | |  | | 0.0010766 | |  | | 2187.000 |  | 0.384094 |  | 0.7009461 |  |
| AQ ✻ timebin12 |  | AQ ✻ 13 - 1 | | |  | 4.8511e-5 | | |  | 4.5927e-4 | | |  | -8.5165e−4 | |  | | 9.4867e-4 | |  | | 2187.000 |  | 0.105625 |  | 0.9158893 |  |
| AQ ✻ timebin13 |  | AQ ✻ 14 - 1 | | |  | 8.9021e-5 | | |  | 4.5927e-4 | | |  | -8.1114e−4 | |  | | 9.8918e-4 | |  | | 2187.000 |  | 0.193831 |  | 0.8463261 |  |
| SCC ✻ directionToS1W21 |  | SCC ✻ 2 - 1 | | |  | 1.7379e-4 | | |  | 1.0978e-4 | | |  | -4.1381e−5 | |  | | 3.8896e-4 | |  | | 2187.000 |  | 1.583030 |  | 0.1135593 |  |
| SCC ✻ timebin1 |  | SCC ✻ 2 - 1 | | |  | -5.3864e−6 | | |  | 2.9046e-4 | | |  | -5.7467e−4 | |  | | 5.6390e-4 | |  | | 2187.000 |  | -0.018545 |  | 0.9852061 |  |
| SCC ✻ timebin2 |  | SCC ✻ 3 - 1 | | |  | -4.3369e−5 | | |  | 2.9046e-4 | | |  | -6.1265e−4 | |  | | 5.2591e-4 | |  | | 2187.000 |  | -0.149315 |  | 0.8813188 |  |
| SCC ✻ timebin3 |  | SCC ✻ 4 - 1 | | |  | -1.2930e−4 | | |  | 2.9046e-4 | | |  | -6.9858e−4 | |  | | 4.3998e-4 | |  | | 2187.000 |  | -0.445171 |  | 0.6562406 |  |
| SCC ✻ timebin4 |  | SCC ✻ 5 - 1 | | |  | -1.7132e−4 | | |  | 2.9046e-4 | | |  | -7.4060e−4 | |  | | 3.9796e-4 | |  | | 2187.000 |  | -0.589836 |  | 0.5553618 |  |
| SCC ✻ timebin5 |  | SCC ✻ 6 - 1 | | |  | -4.4966e−4 | | |  | 2.9046e-4 | | |  | -0.0010189 | |  | | 1.1962e-4 | |  | | 2187.000 |  | -1.548126 |  | 0.1217367 |  |
| SCC ✻ timebin6 |  | SCC ✻ 7 - 1 | | |  | -5.2456e−4 | | |  | 2.9046e-4 | | |  | -0.0010938 | |  | | 4.4720e-5 | |  | | 2187.000 |  | -1.805999 |  | 0.0710559 |  |
| SCC ✻ timebin7 |  | SCC ✻ 8 - 1 | | |  | -6.8205e−5 | | |  | 2.9046e-4 | | |  | -6.3749e−4 | |  | | 5.0108e-4 | |  | | 2187.000 |  | -0.234821 |  | 0.8143693 |  |
| SCC ✻ timebin8 |  | SCC ✻ 9 - 1 | | |  | -1.7473e−4 | | |  | 2.9046e-4 | | |  | -7.4401e−4 | |  | | 3.9456e-4 | |  | | 2187.000 |  | -0.601558 |  | 0.5475310 |  |
| SCC ✻ timebin9 |  | SCC ✻ 10 - 1 | | |  | -1.7026e−4 | | |  | 2.9046e-4 | | |  | -7.3954e−4 | |  | | 3.9902e-4 | |  | | 2187.000 |  | -0.586184 |  | 0.5578123 |  |
| SCC ✻ timebin10 |  | SCC ✻ 11 - 1 | | |  | -4.6390e−6 | | |  | 2.9046e-4 | | |  | -5.7392e−4 | |  | | 5.6464e-4 | |  | | 2187.000 |  | -0.015971 |  | 0.9872586 |  |
| SCC ✻ timebin11 |  | SCC ✻ 12 - 1 | | |  | 2.0094e-4 | | |  | 2.9046e-4 | | |  | -3.6834e−4 | |  | | 7.7022e-4 | |  | | 2187.000 |  | 0.691817 |  | 0.4891256 |  |
| SCC ✻ timebin12 |  | SCC ✻ 13 - 1 | | |  | 7.0986e-5 | | |  | 2.9046e-4 | | |  | -4.9830e−4 | |  | | 6.4027e-4 | |  | | 2187.000 |  | 0.244396 |  | 0.8069470 |  |
| SCC ✻ timebin13 |  | SCC ✻ 14 - 1 | | |  | 6.9727e-5 | | |  | 2.9046e-4 | | |  | -4.9956e−4 | |  | | 6.3901e-4 | |  | | 2187.000 |  | 0.240060 |  | 0.8103064 |  |
| AQ ✻ directionToS1W21 ✻ timebin1 |  | AQ ✻ 2 - 1 ✻ 2 - 1 | | |  | 4.2432e-4 | | |  | 9.1855e-4 | | |  | -0.0013760 | |  | | 0.0022246 | |  | | 2187.000 |  | 0.461950 |  | 0.6441634 |  |
| AQ ✻ directionToS1W21 ✻ timebin2 |  | AQ ✻ 2 - 1 ✻ 3 - 1 | | |  | 3.2589e-4 | | |  | 9.1855e-4 | | |  | -0.0014744 | |  | | 0.0021262 | |  | | 2187.000 |  | 0.354785 |  | 0.7227847 |  |
| AQ ✻ directionToS1W21 ✻ timebin3 |  | AQ ✻ 2 - 1 ✻ 4 - 1 | | |  | 6.2735e-5 | | |  | 9.1855e-4 | | |  | -0.0017376 | |  | | 0.0018631 | |  | | 2187.000 |  | 0.068299 |  | 0.9455542 |  |
| AQ ✻ directionToS1W21 ✻ timebin4 |  | AQ ✻ 2 - 1 ✻ 5 - 1 | | |  | -1.8784e−4 | | |  | 9.1855e-4 | | |  | -0.0019882 | |  | | 0.0016125 | |  | | 2187.000 |  | -0.204498 |  | 0.8379832 |  |
| AQ ✻ directionToS1W21 ✻ timebin5 |  | AQ ✻ 2 - 1 ✻ 6 - 1 | | |  | 0.0017614 | | |  | 9.1855e-4 | | |  | -3.8958e−5 | |  | | 0.0035617 | |  | | 2187.000 |  | 1.917551 |  | 0.0552981 |  |
| AQ ✻ directionToS1W21 ✻ timebin6 |  | AQ ✻ 2 - 1 ✻ 7 - 1 | | |  | 0.0017366 | | |  | 9.1855e-4 | | |  | -6.3734e−5 | |  | | 0.0035369 | |  | | 2187.000 |  | 1.890579 |  | 0.0588127 |  |
| AQ ✻ directionToS1W21 ✻ timebin7 |  | AQ ✻ 2 - 1 ✻ 8 - 1 | | |  | -5.7107e−4 | | |  | 9.1855e-4 | | |  | -0.0023714 | |  | | 0.0012292 | |  | | 2187.000 |  | -0.621709 |  | 0.5341983 |  |
| AQ ✻ directionToS1W21 ✻ timebin8 |  | AQ ✻ 2 - 1 ✻ 9 - 1 | | |  | 2.1634e-4 | | |  | 9.1855e-4 | | |  | -0.0015840 | |  | | 0.0020167 | |  | | 2187.000 |  | 0.235529 |  | 0.8138199 |  |
| AQ ✻ directionToS1W21 ✻ timebin9 |  | AQ ✻ 2 - 1 ✻ 10 - 1 | | |  | 1.2957e-4 | | |  | 9.1855e-4 | | |  | -0.0016707 | |  | | 0.0019299 | |  | | 2187.000 |  | 0.141063 |  | 0.8878334 |  |
| AQ ✻ directionToS1W21 ✻ timebin10 |  | AQ ✻ 2 - 1 ✻ 11 - 1 | | |  | 1.7069e-4 | | |  | 9.1855e-4 | | |  | -0.0016296 | |  | | 0.0019710 | |  | | 2187.000 |  | 0.185831 |  | 0.8525945 |  |
| AQ ✻ directionToS1W21 ✻ timebin11 |  | AQ ✻ 2 - 1 ✻ 12 - 1 | | |  | 3.1434e-4 | | |  | 9.1855e-4 | | |  | -0.0014860 | |  | | 0.0021147 | |  | | 2187.000 |  | 0.342219 |  | 0.7322187 |  |
| AQ ✻ directionToS1W21 ✻ timebin12 |  | AQ ✻ 2 - 1 ✻ 13 - 1 | | |  | 5.0071e-4 | | |  | 9.1855e-4 | | |  | -0.0012996 | |  | | 0.0023010 | |  | | 2187.000 |  | 0.545107 |  | 0.5857355 |  |
| AQ ✻ directionToS1W21 ✻ timebin13 |  | AQ ✻ 2 - 1 ✻ 14 - 1 | | |  | -2.9326e−5 | | |  | 9.1855e-4 | | |  | -0.0018296 | |  | | 0.0017710 | |  | | 2187.000 |  | -0.031927 |  | 0.9745334 |  |
| SCC ✻ directionToS1W21 ✻ timebin1 |  | SCC ✻ 2 - 1 ✻ 2 - 1 | | |  | -3.9439e−4 | | |  | 5.8091e-4 | | |  | -0.0015330 | |  | | 7.4417e-4 | |  | | 2187.000 |  | -0.678919 |  | 0.4972612 |  |
| SCC ✻ directionToS1W21 ✻ timebin2 |  | SCC ✻ 2 - 1 ✻ 3 - 1 | | |  | -7.0108e−4 | | |  | 5.8091e-4 | | |  | -0.0018396 | |  | | 4.3748e-4 | |  | | 2187.000 |  | -1.206869 |  | 0.2276130 |  |
| SCC ✻ directionToS1W21 ✻ timebin3 |  | SCC ✻ 2 - 1 ✻ 4 - 1 | | |  | -6.1879e−4 | | |  | 5.8091e-4 | | |  | -0.0017574 | |  | | 5.1977e-4 | |  | | 2187.000 |  | -1.065214 |  | 0.2868966 |  |
| SCC ✻ directionToS1W21 ✻ timebin4 |  | SCC ✻ 2 - 1 ✻ 5 - 1 | | |  | -1.7232e−4 | | |  | 5.8091e-4 | | |  | -0.0013109 | |  | | 9.6625e-4 | |  | | 2187.000 |  | -0.296630 |  | 0.7667770 |  |
| SCC ✻ directionToS1W21 ✻ timebin5 |  | SCC ✻ 2 - 1 ✻ 6 - 1 | | |  | 4.0361e-4 | | |  | 5.8091e-4 | | |  | -7.3495e−4 | |  | | 0.0015422 | |  | | 2187.000 |  | 0.694789 |  | 0.4872616 |  |
| SCC ✻ directionToS1W21 ✻ timebin6 |  | SCC ✻ 2 - 1 ✻ 7 - 1 | | |  | 2.0535e-4 | | |  | 5.8091e-4 | | |  | -9.3322e−4 | |  | | 0.0013439 | |  | | 2187.000 |  | 0.353488 |  | 0.7237569 |  |
| SCC ✻ directionToS1W21 ✻ timebin7 |  | SCC ✻ 2 - 1 ✻ 8 - 1 | | |  | -2.5055e−4 | | |  | 5.8091e-4 | | |  | -0.0013891 | |  | | 8.8801e-4 | |  | | 2187.000 |  | -0.431308 |  | 0.6662870 |  |
| SCC ✻ directionToS1W21 ✻ timebin8 |  | SCC ✻ 2 - 1 ✻ 9 - 1 | | |  | -3.9558e−4 | | |  | 5.8091e-4 | | |  | -0.0015341 | |  | | 7.4298e-4 | |  | | 2187.000 |  | -0.680973 |  | 0.4959608 |  |
| SCC ✻ directionToS1W21 ✻ timebin9 |  | SCC ✻ 2 - 1 ✻ 10 - 1 | | |  | -4.1989e−4 | | |  | 5.8091e-4 | | |  | -0.0015585 | |  | | 7.1867e-4 | |  | | 2187.000 |  | -0.722814 |  | 0.4698712 |  |
| SCC ✻ directionToS1W21 ✻ timebin10 |  | SCC ✻ 2 - 1 ✻ 11 - 1 | | |  | -1.6754e−4 | | |  | 5.8091e-4 | | |  | -0.0013061 | |  | | 9.7103e-4 | |  | | 2187.000 |  | -0.288402 |  | 0.7730660 |  |
| SCC ✻ directionToS1W21 ✻ timebin11 |  | SCC ✻ 2 - 1 ✻ 12 - 1 | | |  | -2.1803e−4 | | |  | 5.8091e-4 | | |  | -0.0013566 | |  | | 9.2053e-4 | |  | | 2187.000 |  | -0.375326 |  | 0.7074543 |  |
| SCC ✻ directionToS1W21 ✻ timebin12 |  | SCC ✻ 2 - 1 ✻ 13 - 1 | | |  | -1.2521e−4 | | |  | 5.8091e-4 | | |  | -0.0012638 | |  | | 0.0010134 | |  | | 2187.000 |  | -0.215539 |  | 0.8293671 |  |
| SCC ✻ directionToS1W21 ✻ timebin13 |  | SCC ✻ 2 - 1 ✻ 14 - 1 | | |  | -5.6377e−4 | | |  | 5.8091e-4 | | |  | -0.0017023 | |  | | 5.7480e-4 | |  | | 2187.000 |  | -0.970485 |  | 0.3319122 |  |
|  | | | | | | | | | | | | | | | | | | | | | | | | | | | |
| Random Components | | | | | | | | | | | | | | | | | | | | |  |  |  |  |  |  |  |
|  | | |  |  | | |  |  | | |  |  | | |  | |  | |  | |  |  |  |  |  |  |  |
| **Groups** | | | | **Name** | | | | **SD** | | | | **Variance** | | | | | **ICC** | | | |  |  |  |  |  |  |  |
| anonymousId.qual | | |  | (Intercept) | | |  | 0.047812 | | |  | 0.0022860 | | |  | | 0.73887 | |  | |  |  |  |  |  |  |  |
| Residual | | |  |  | | |  | 0.028423 | | |  | 8.0789e-4 | | |  | |  | |  | |  |  |  |  |  |  |  |
| Note. Number of Obs: 2352 , groups: anonymousId.qual 84 | | | | | | | | | | | | | | | | | | | | |  |  |  |  |  |  |  |
|  | | | | | | | | | | | | | | | | | | | | |  |  |  |  |  |  |  |

Post Hoc Tests

| Post Hoc Comparisons - timebin | | | | | | | | | | | | | | | |
| --- | --- | --- | --- | --- | --- | --- | --- | --- | --- | --- | --- | --- | --- | --- | --- |
| **Comparison** | | | | | |  | | | | | | | | | |
| **timebin** | |  | | **timebin** | | **Difference** | | **SE** | | **t** | | **df** | | **p_bonferroni_** | |
| 2 |  | - |  | 10 |  | 3.9640e-4 |  | 0.0031013 |  | 0.1278192 |  | 2187.0 |  | 1.0000000 |  |
| 2 |  | - |  | 11 |  | 0.0010433 |  | 0.0031013 |  | 0.3364021 |  | 2187.0 |  | 1.0000000 |  |
| 2 |  | - |  | 12 |  | 0.0018035 |  | 0.0031013 |  | 0.5815319 |  | 2187.0 |  | 1.0000000 |  |
| 2 |  | - |  | 13 |  | 0.0064767 |  | 0.0031013 |  | 2.0884155 |  | 2187.0 |  | 1.0000000 |  |
| 2 |  | - |  | 14 |  | 0.0065007 |  | 0.0031013 |  | 2.0961545 |  | 2187.0 |  | 1.0000000 |  |
| 2 |  | - |  | 3 |  | -0.0018190 |  | 0.0031013 |  | -0.5865292 |  | 2187.0 |  | 1.0000000 |  |
| 2 |  | - |  | 4 |  | -0.0125212 |  | 0.0031013 |  | -4.0374591 |  | 2187.0 |  | 0.0050864 |  |
| 2 |  | - |  | 6 |  | -0.0614110 |  | 0.0031013 |  | -19.8019962 |  | 2187.0 |  | 1.9038e0-78 |  |
| 2 |  | - |  | 5 |  | -0.0243085 |  | 0.0031013 |  | -7.8382768 |  | 2187.0 |  | 6.4368e0-13 |  |
| 2 |  | - |  | 7 |  | -0.0678832 |  | 0.0031013 |  | -21.8889522 |  | 2187.0 |  | 3.0684e0-94 |  |
| 2 |  | - |  | 8 |  | -0.0164752 |  | 0.0031013 |  | -5.3124415 |  | 2187.0 |  | 1.0841e00-5 |  |
| 2 |  | - |  | 9 |  | -0.0089823 |  | 0.0031013 |  | -2.8963358 |  | 2187.0 |  | 0.3469809 |  |
| 1 |  | - |  | 2 |  | -0.0027023 |  | 0.0031013 |  | -0.8713648 |  | 2187.0 |  | 1.0000000 |  |
| 1 |  | - |  | 10 |  | -0.0023059 |  | 0.0031013 |  | -0.7435456 |  | 2187.0 |  | 1.0000000 |  |
| 1 |  | - |  | 11 |  | -0.0016591 |  | 0.0031013 |  | -0.5349627 |  | 2187.0 |  | 1.0000000 |  |
| 1 |  | - |  | 12 |  | -8.9884e−4 |  | 0.0031013 |  | -0.2898328 |  | 2187.0 |  | 1.0000000 |  |
| 1 |  | - |  | 13 |  | 0.0037744 |  | 0.0031013 |  | 1.2170507 |  | 2187.0 |  | 1.0000000 |  |
| 1 |  | - |  | 14 |  | 0.0037984 |  | 0.0031013 |  | 1.2247897 |  | 2187.0 |  | 1.0000000 |  |
| 1 |  | - |  | 3 |  | -0.0045213 |  | 0.0031013 |  | -1.4578940 |  | 2187.0 |  | 1.0000000 |  |
| 1 |  | - |  | 4 |  | -0.0152235 |  | 0.0031013 |  | -4.9088238 |  | 2187.0 |  | 8.9539e00-5 |  |
| 1 |  | - |  | 6 |  | -0.0641133 |  | 0.0031013 |  | -20.6733609 |  | 2187.0 |  | 6.5133e0-85 |  |
| 1 |  | - |  | 5 |  | -0.0270108 |  | 0.0031013 |  | -8.7096415 |  | 2187.0 |  | 5.3667e0-16 |  |
| 1 |  | - |  | 7 |  | -0.0705855 |  | 0.0031013 |  | -22.7603169 |  | 2187.0 |  | 3.9192e-101 |  |
| 1 |  | - |  | 8 |  | -0.0191775 |  | 0.0031013 |  | -6.1838063 |  | 2187.0 |  | 6.7751e00-8 |  |
| 1 |  | - |  | 9 |  | -0.0116846 |  | 0.0031013 |  | -3.7677005 |  | 2187.0 |  | 0.0153895 |  |
| 10 |  | - |  | 11 |  | 6.4687e-4 |  | 0.0031013 |  | 0.2085829 |  | 2187.0 |  | 1.0000000 |  |
| 10 |  | - |  | 12 |  | 0.0014071 |  | 0.0031013 |  | 0.4537127 |  | 2187.0 |  | 1.0000000 |  |
| 10 |  | - |  | 13 |  | 0.0060803 |  | 0.0031013 |  | 1.9605963 |  | 2187.0 |  | 1.0000000 |  |
| 10 |  | - |  | 14 |  | 0.0061043 |  | 0.0031013 |  | 1.9683353 |  | 2187.0 |  | 1.0000000 |  |
| 11 |  | - |  | 12 |  | 7.6021e-4 |  | 0.0031013 |  | 0.2451298 |  | 2187.0 |  | 1.0000000 |  |
| 11 |  | - |  | 13 |  | 0.0054334 |  | 0.0031013 |  | 1.7520134 |  | 2187.0 |  | 1.0000000 |  |
| 11 |  | - |  | 14 |  | 0.0054574 |  | 0.0031013 |  | 1.7597524 |  | 2187.0 |  | 1.0000000 |  |
| 12 |  | - |  | 13 |  | 0.0046732 |  | 0.0031013 |  | 1.5068835 |  | 2187.0 |  | 1.0000000 |  |
| 12 |  | - |  | 14 |  | 0.0046972 |  | 0.0031013 |  | 1.5146225 |  | 2187.0 |  | 1.0000000 |  |
| 13 |  | - |  | 14 |  | 2.4001e-5 |  | 0.0031013 |  | 0.0077390 |  | 2187.0 |  | 1.0000000 |  |
| 3 |  | - |  | 10 |  | 0.0022154 |  | 0.0031013 |  | 0.7143484 |  | 2187.0 |  | 1.0000000 |  |
| 3 |  | - |  | 11 |  | 0.0028622 |  | 0.0031013 |  | 0.9229313 |  | 2187.0 |  | 1.0000000 |  |
| 3 |  | - |  | 12 |  | 0.0036225 |  | 0.0031013 |  | 1.1680612 |  | 2187.0 |  | 1.0000000 |  |
| 3 |  | - |  | 13 |  | 0.0082957 |  | 0.0031013 |  | 2.6749447 |  | 2187.0 |  | 0.6852157 |  |
| 3 |  | - |  | 14 |  | 0.0083197 |  | 0.0031013 |  | 2.6826837 |  | 2187.0 |  | 0.6696129 |  |
| 3 |  | - |  | 4 |  | -0.0107022 |  | 0.0031013 |  | -3.4509299 |  | 2187.0 |  | 0.0518028 |  |
| 3 |  | - |  | 6 |  | -0.0595920 |  | 0.0031013 |  | -19.2154670 |  | 2187.0 |  | 3.3369e0-74 |  |
| 3 |  | - |  | 5 |  | -0.0224895 |  | 0.0031013 |  | -7.2517475 |  | 2187.0 |  | 5.1711e0-11 |  |
| 3 |  | - |  | 7 |  | -0.0660642 |  | 0.0031013 |  | -21.3024230 |  | 2187.0 |  | 1.0729e0-89 |  |
| 3 |  | - |  | 8 |  | -0.0146562 |  | 0.0031013 |  | -4.7259123 |  | 2187.0 |  | 2.2172e00-4 |  |
| 3 |  | - |  | 9 |  | -0.0071633 |  | 0.0031013 |  | -2.3098066 |  | 2187.0 |  | 1.0000000 |  |
| 4 |  | - |  | 10 |  | 0.0129176 |  | 0.0031013 |  | 4.1652783 |  | 2187.0 |  | 0.0029398 |  |
| 4 |  | - |  | 11 |  | 0.0135644 |  | 0.0031013 |  | 4.3738612 |  | 2187.0 |  | 0.0011630 |  |
| 4 |  | - |  | 12 |  | 0.0143247 |  | 0.0031013 |  | 4.6189910 |  | 2187.0 |  | 3.7129e00-4 |  |
| 4 |  | - |  | 13 |  | 0.0189979 |  | 0.0031013 |  | 6.1258745 |  | 2187.0 |  | 9.7042e00-8 |  |
| 4 |  | - |  | 14 |  | 0.0190219 |  | 0.0031013 |  | 6.1336135 |  | 2187.0 |  | 9.2511e00-8 |  |
| 4 |  | - |  | 6 |  | -0.0488898 |  | 0.0031013 |  | -15.7645371 |  | 2187.0 |  | 3.7273e0-51 |  |
| 4 |  | - |  | 5 |  | -0.0117873 |  | 0.0031013 |  | -3.8008177 |  | 2187.0 |  | 0.0134827 |  |
| 4 |  | - |  | 7 |  | -0.0553620 |  | 0.0031013 |  | -17.8514931 |  | 2187.0 |  | 1.0908e0-64 |  |
| 4 |  | - |  | 8 |  | -0.0039540 |  | 0.0031013 |  | -1.2749824 |  | 2187.0 |  | 1.0000000 |  |
| 4 |  | - |  | 9 |  | 0.0035389 |  | 0.0031013 |  | 1.1411233 |  | 2187.0 |  | 1.0000000 |  |
| 6 |  | - |  | 10 |  | 0.0618074 |  | 0.0031013 |  | 19.9298154 |  | 2187.0 |  | 2.2032e0-79 |  |
| 6 |  | - |  | 11 |  | 0.0624542 |  | 0.0031013 |  | 20.1383983 |  | 2187.0 |  | 6.3954e0-81 |  |
| 6 |  | - |  | 12 |  | 0.0632145 |  | 0.0031013 |  | 20.3835281 |  | 2187.0 |  | 9.6695e0-83 |  |
| 6 |  | - |  | 13 |  | 0.0678877 |  | 0.0031013 |  | 21.8904116 |  | 2187.0 |  | 2.9889e0-94 |  |
| 6 |  | - |  | 14 |  | 0.0679117 |  | 0.0031013 |  | 21.8981507 |  | 2187.0 |  | 2.6003e0-94 |  |
| 6 |  | - |  | 7 |  | -0.0064722 |  | 0.0031013 |  | -2.0869560 |  | 2187.0 |  | 1.0000000 |  |
| 6 |  | - |  | 8 |  | 0.0449358 |  | 0.0031013 |  | 14.4895547 |  | 2187.0 |  | 1.5341e0-43 |  |
| 6 |  | - |  | 9 |  | 0.0524287 |  | 0.0031013 |  | 16.9056604 |  | 2187.0 |  | 2.1394e0-58 |  |
| 5 |  | - |  | 10 |  | 0.0247049 |  | 0.0031013 |  | 7.9660960 |  | 2187.0 |  | 2.3745e0-13 |  |
| 5 |  | - |  | 11 |  | 0.0253517 |  | 0.0031013 |  | 8.1746789 |  | 2187.0 |  | 4.5192e0-14 |  |
| 5 |  | - |  | 12 |  | 0.0261119 |  | 0.0031013 |  | 8.4198087 |  | 2187.0 |  | 6.1182e0-15 |  |
| 5 |  | - |  | 13 |  | 0.0307852 |  | 0.0031013 |  | 9.9266922 |  | 2187.0 |  | 8.7081e0-21 |  |
| 5 |  | - |  | 14 |  | 0.0308092 |  | 0.0031013 |  | 9.9344312 |  | 2187.0 |  | 8.0848e0-21 |  |
| 5 |  | - |  | 6 |  | -0.0371025 |  | 0.0031013 |  | -11.9637194 |  | 2187.0 |  | 4.8773e0-30 |  |
| 5 |  | - |  | 7 |  | -0.0435747 |  | 0.0031013 |  | -14.0506754 |  | 2187.0 |  | 4.8544e0-41 |  |
| 5 |  | - |  | 8 |  | 0.0078333 |  | 0.0031013 |  | 2.5258353 |  | 2187.0 |  | 1.0000000 |  |
| 5 |  | - |  | 9 |  | 0.0153262 |  | 0.0031013 |  | 4.9419410 |  | 2187.0 |  | 7.5729e00-5 |  |
| 7 |  | - |  | 10 |  | 0.0682796 |  | 0.0031013 |  | 22.0167714 |  | 2187.0 |  | 3.0639e0-95 |  |
| 7 |  | - |  | 11 |  | 0.0689264 |  | 0.0031013 |  | 22.2253543 |  | 2187.0 |  | 7.0059e0-97 |  |
| 7 |  | - |  | 12 |  | 0.0696866 |  | 0.0031013 |  | 22.4704841 |  | 2187.0 |  | 8.0313e0-99 |  |
| 7 |  | - |  | 13 |  | 0.0743599 |  | 0.0031013 |  | 23.9773676 |  | 2187.0 |  | 4.9247e-111 |  |
| 7 |  | - |  | 14 |  | 0.0743839 |  | 0.0031013 |  | 23.9851066 |  | 2187.0 |  | 4.2506e-111 |  |
| 7 |  | - |  | 8 |  | 0.0514079 |  | 0.0031013 |  | 16.5765107 |  | 2187.0 |  | 2.8777e0-56 |  |
| 7 |  | - |  | 9 |  | 0.0589009 |  | 0.0031013 |  | 18.9926164 |  | 2187.0 |  | 1.2947e0-72 |  |
| 8 |  | - |  | 10 |  | 0.0168716 |  | 0.0031013 |  | 5.4402607 |  | 2187.0 |  | 5.3821e00-6 |  |
| 8 |  | - |  | 11 |  | 0.0175185 |  | 0.0031013 |  | 5.6488436 |  | 2187.0 |  | 1.6614e00-6 |  |
| 8 |  | - |  | 12 |  | 0.0182787 |  | 0.0031013 |  | 5.8939734 |  | 2187.0 |  | 3.9634e00-7 |  |
| 8 |  | - |  | 13 |  | 0.0229519 |  | 0.0031013 |  | 7.4008570 |  | 2187.0 |  | 1.7466e0-11 |  |
| 8 |  | - |  | 14 |  | 0.0229759 |  | 0.0031013 |  | 7.4085960 |  | 2187.0 |  | 1.6500e0-11 |  |
| 8 |  | - |  | 9 |  | 0.0074930 |  | 0.0031013 |  | 2.4161057 |  | 2187.0 |  | 1.0000000 |  |
| 9 |  | - |  | 10 |  | 0.0093787 |  | 0.0031013 |  | 3.0241550 |  | 2187.0 |  | 0.2295277 |  |
| 9 |  | - |  | 11 |  | 0.0100255 |  | 0.0031013 |  | 3.2327379 |  | 2187.0 |  | 0.1132348 |  |
| 9 |  | - |  | 12 |  | 0.0107857 |  | 0.0031013 |  | 3.4778677 |  | 2187.0 |  | 0.0468919 |  |
| 9 |  | - |  | 13 |  | 0.0154590 |  | 0.0031013 |  | 4.9847512 |  | 2187.0 |  | 6.0892e00-5 |  |
| 9 |  | - |  | 14 |  | 0.0154830 |  | 0.0031013 |  | 4.9924902 |  | 2187.0 |  | 5.8528e00-5 |  |
|  | | | | | | | | | | | | | | | |

Simple Effects

| Simple effects of AQ : Omnibus Tests | | | | | | | | | | | | | | | | |  |  |  |  |  |  |  |  |
| --- | --- | --- | --- | --- | --- | --- | --- | --- | --- | --- | --- | --- | --- | --- | --- | --- | --- | --- | --- | --- | --- | --- | --- | --- |
| **Moderator levels** | | | |  | | | | | | | | | | | | |  |  |  |  |  |  |  |  |
| **timebin** | | | | **F** | | | **Num df** | | | | **Den df** | | **p** | | | |  |  |  |  |  |  |  |  |
| 1 | |  | | 0.1660000 | |  | 1.0000 | |  | | 105.02 |  | 0.68419 | | |  |  |  |  |  |  |  |  |  |
| 2 | |  | | 0.4710000 | |  | 1.0000 | |  | | 105.02 |  | 0.49423 | | |  |  |  |  |  |  |  |  |  |
| 3 | |  | | 0.0600000 | |  | 1.0000 | |  | | 105.02 |  | 0.80676 | | |  |  |  |  |  |  |  |  |  |
| 4 | |  | | 0.0630000 | |  | 1.0000 | |  | | 105.02 |  | 0.80274 | | |  |  |  |  |  |  |  |  |  |
| 5 | |  | | 0.1540000 | |  | 1.0000 | |  | | 105.02 |  | 0.69528 | | |  |  |  |  |  |  |  |  |  |
| 6 | |  | | 1.6440000 | |  | 1.0000 | |  | | 105.02 |  | 0.20255 | | |  |  |  |  |  |  |  |  |  |
| 7 | |  | | 0.8210000 | |  | 1.0000 | |  | | 105.02 |  | 0.36697 | | |  |  |  |  |  |  |  |  |  |
| 8 | |  | | 0.2190000 | |  | 1.0000 | |  | | 105.02 |  | 0.64106 | | |  |  |  |  |  |  |  |  |  |
| 9 | |  | | 0.0050000 | |  | 1.0000 | |  | | 105.02 |  | 0.94557 | | |  |  |  |  |  |  |  |  |  |
| 10 | |  | | 0.0130000 | |  | 1.0000 | |  | | 105.02 |  | 0.91005 | | |  |  |  |  |  |  |  |  |  |
| 11 | |  | | 0.1980000 | |  | 1.0000 | |  | | 105.02 |  | 0.65729 | | |  |  |  |  |  |  |  |  |  |
| 12 | |  | | 0.3760000 | |  | 1.0000 | |  | | 105.02 |  | 0.54088 | | |  |  |  |  |  |  |  |  |  |
| 13 | |  | | 0.2160000 | |  | 1.0000 | |  | | 105.02 |  | 0.64330 | | |  |  |  |  |  |  |  |  |  |
| 14 | |  | | 0.2620000 | |  | 1.0000 | |  | | 105.02 |  | 0.60997 | | |  |  |  |  |  |  |  |  |  |
|  | | | | | | | | | | | | | | | | |  |  |  |  |  |  |  |  |
| Simple effects of AQ : Parameter estimates | | | | | | | | | | | | | | | | | | | | | | | | |
| **Moderator levels** | | |  | | | | | | | **95% Confidence Interval** | | | | | | | | |  | | | | | |
| **timebin** | | | **Estimate** | | | **SE** | | | | **Lower** | | | | | **Upper** | | | | **df** | | **t** | | **p** | |
| 1 |  | | 3.4996e-4 | |  | 8.5799e-4 | |  | | -0.0013513 | | | |  | 0.0020512 | | |  | 105.02 |  | 0.407888 |  | 0.68419 |  |
| 2 |  | | 5.8858e-4 | |  | 8.5799e-4 | |  | | -0.0011127 | | | |  | 0.0022898 | | |  | 105.02 |  | 0.685992 |  | 0.49423 |  |
| 3 |  | | 2.1040e-4 | |  | 8.5799e-4 | |  | | -0.0014908 | | | |  | 0.0019116 | | |  | 105.02 |  | 0.245223 |  | 0.80676 |  |
| 4 |  | | 2.1488e-4 | |  | 8.5799e-4 | |  | | -0.0014864 | | | |  | 0.0019161 | | |  | 105.02 |  | 0.250441 |  | 0.80274 |  |
| 5 |  | | -3.3700e−4 | |  | 8.5799e-4 | |  | | -0.0020382 | | | |  | 0.0013642 | | |  | 105.02 |  | -0.392779 |  | 0.69528 |  |
| 6 |  | | -0.0011002 | |  | 8.5799e-4 | |  | | -0.0028015 | | | |  | 6.0100e-4 | | |  | 105.02 |  | -1.282336 |  | 0.20255 |  |
| 7 |  | | -7.7741e−4 | |  | 8.5799e-4 | |  | | -0.0024786 | | | |  | 9.2383e-4 | | |  | 105.02 |  | -0.906079 |  | 0.36697 |  |
| 8 |  | | -4.0118e−4 | |  | 8.5799e-4 | |  | | -0.0021024 | | | |  | 0.0013001 | | |  | 105.02 |  | -0.467575 |  | 0.64106 |  |
| 9 |  | | -5.8718e−5 | |  | 8.5799e-4 | |  | | -0.0017600 | | | |  | 0.0016425 | | |  | 105.02 |  | -0.068436 |  | 0.94557 |  |
| 10 |  | | 9.7167e-5 | |  | 8.5799e-4 | |  | | -0.0016041 | | | |  | 0.0017984 | | |  | 105.02 |  | 0.113249 |  | 0.91005 |  |
| 11 |  | | 3.8174e-4 | |  | 8.5799e-4 | |  | | -0.0013195 | | | |  | 0.0020830 | | |  | 105.02 |  | 0.444919 |  | 0.65729 |  |
| 12 |  | | 5.2637e-4 | |  | 8.5799e-4 | |  | | -0.0011749 | | | |  | 0.0022276 | | |  | 105.02 |  | 0.613489 |  | 0.54088 |  |
| 13 |  | | 3.9848e-4 | |  | 8.5799e-4 | |  | | -0.0013028 | | | |  | 0.0020997 | | |  | 105.02 |  | 0.464428 |  | 0.64330 |  |
| 14 |  | | 4.3899e-4 | |  | 8.5799e-4 | |  | | -0.0012623 | | | |  | 0.0021402 | | |  | 105.02 |  | 0.511644 |  | 0.60997 |  |
| Note. Simple effects are estimated keeping constant other independent variable(s) in the model | | | | | | | | | | | | | | | | | | | | | | | | |
|  | | | | | | | | | | | | | | | | | | | | | | | | |

Effects Plots


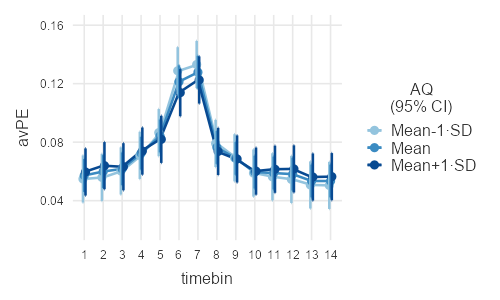


## Speed around Environment Switches

dMouse = distance travelled by mouse on each frame (ie. unadjusted speed)

Mixed Model

| Model Info | | | |
| --- | --- | --- | --- |
|  |  |  |  |
| **Info** | |  | |
| Estimate |  | Linear mixed model fit by REML |  |
| Call |  | dMouse ~ 1 + AQ + SCC + directionToS1W2 + timebin + nFrames + sdWaveTime + directionToS1W2:timebin + directionToS1W2:AQ + timebin:AQ + directionToS1W2:SCC + timebin:SCC + timebin:directionToS1W2:AQ + timebin:directionToS1W2:SCC+( 1 \| anonymousId.qual ) |  |
| AIC |  | -18940.389800 |  |
| BIC |  | -17027.990800 |  |
| LogLikel. |  | 8855.568334 |  |
| R-squared Marginal |  | 0.086383 |  |
| R-squared Conditional |  | 0.841641 |  |
| Converged |  | yes |  |
| Optimizer |  | bobyqa |  |
|  | | | |

Model Results

| Fixed Effect Omnibus tests | | | | | | | | | |
| --- | --- | --- | --- | --- | --- | --- | --- | --- | --- |
|  |  |  |  |  |  |  |  |  |  |
|  | | **F** | | **Num df** | | **Den df** | | **p** | |
| AQ |  | 1.670843 |  | 1 |  | 79.000 |  | 0.199914 |  |
| SCC |  | 0.010337 |  | 1 |  | 79.000 |  | 0.919274 |  |
| directionToS1W2 |  | 0.075823 |  | 1 |  | 2187.000 |  | 0.783066 |  |
| timebin |  | 37.915840 |  | 13 |  | 2187.000 |  | 5.0418e-87 |  |
| nFrames |  | 0.562093 |  | 1 |  | 79.000 |  | 0.455645 |  |
| sdWaveTime |  | 3.077427 |  | 1 |  | 79.000 |  | 0.083264 |  |
| directionToS1W2 ✻ timebin |  | 0.735715 |  | 13 |  | 2187.000 |  | 0.728867 |  |
| AQ ✻ directionToS1W2 |  | 0.561232 |  | 1 |  | 2187.000 |  | 0.453845 |  |
| AQ ✻ timebin |  | 2.916186 |  | 13 |  | 2187.000 |  | 3.2389e0-4 |  |
| SCC ✻ directionToS1W2 |  | 1.629102 |  | 1 |  | 2187.000 |  | 0.201963 |  |
| SCC ✻ timebin |  | 0.981092 |  | 13 |  | 2187.000 |  | 0.467374 |  |
| AQ ✻ directionToS1W2 ✻ timebin |  | 0.352053 |  | 13 |  | 2187.000 |  | 0.983257 |  |
| SCC ✻ directionToS1W2 ✻ timebin |  | 0.560577 |  | 13 |  | 2187.000 |  | 0.886390 |  |
| Note. Satterthwaite method for degrees of freedom | | | | | | | | | |
|  | | | | | | | | | |

| Fixed Effects Parameter Estimates | | | | | | | | | | | | | | | | | |
| --- | --- | --- | --- | --- | --- | --- | --- | --- | --- | --- | --- | --- | --- | --- | --- | --- | --- |
|  | | | | | | | | **95% Confidence Interval** | | | |  | | | | | |
| **Names** | | **Effect** | | **Estimate** | | **SE** | | **Lower** | | **Upper** | | **df** | | **t** | | **p** | |
| (Intercept) |  | (Intercept) |  | 0.0088008 |  | 9.2818e-4 |  | 0.0069816 |  | 0.0106200 |  | 79.000 |  | 9.4817286 |  | 1.1186e-14 |  |
| AQ |  | AQ |  | 1.8259e-4 |  | 1.4125e-4 |  | -9.4266e−5 |  | 4.5944e-4 |  | 79.000 |  | 1.2926108 |  | 0.1999136 |  |
| SCC |  | SCC |  | 8.9806e-6 |  | 8.8328e-5 |  | -1.6414e−4 |  | 1.8210e-4 |  | 79.000 |  | 0.1016734 |  | 0.9192735 |  |
| directionToS1W21 |  | 2 - 1 |  | 4.4070e-5 |  | 1.6004e-4 |  | -2.6961e−4 |  | 3.5775e-4 |  | 2187.000 |  | 0.2753591 |  | 0.7830664 |  |
| timebin1 |  | 2 - 1 |  | 4.3541e-4 |  | 4.2344e-4 |  | -3.9451e−4 |  | 0.0012653 |  | 2187.000 |  | 1.0282880 |  | 0.3039281 |  |
| timebin2 |  | 3 - 1 |  | 4.9431e-4 |  | 4.2344e-4 |  | -3.3561e−4 |  | 0.0013242 |  | 2187.000 |  | 1.1673799 |  | 0.2431842 |  |
| timebin3 |  | 4 - 1 |  | 0.0013414 |  | 4.2344e-4 |  | 5.1148e-4 |  | 0.0021713 |  | 2187.000 |  | 3.1678865 |  | 0.0015567 |  |
| timebin4 |  | 5 - 1 |  | 0.0024201 |  | 4.2344e-4 |  | 0.0015902 |  | 0.0032500 |  | 2187.000 |  | 5.7153912 |  | 1.2441e0-8 |  |
| timebin5 |  | 6 - 1 |  | 0.0055162 |  | 4.2344e-4 |  | 0.0046863 |  | 0.0063461 |  | 2187.000 |  | 13.0272725 |  | 2.0354e-37 |  |
| timebin6 |  | 7 - 1 |  | 0.0060148 |  | 4.2344e-4 |  | 0.0051849 |  | 0.0068447 |  | 2187.000 |  | 14.2047545 |  | 7.1862e-44 |  |
| timebin7 |  | 8 - 1 |  | 0.0017882 |  | 4.2344e-4 |  | 9.5826e-4 |  | 0.0026181 |  | 2187.000 |  | 4.2230105 |  | 2.5093e0-5 |  |
| timebin8 |  | 9 - 1 |  | 0.0030519 |  | 4.2344e-4 |  | 0.0022220 |  | 0.0038818 |  | 2187.000 |  | 7.2074377 |  | 7.8149e-13 |  |
| timebin9 |  | 10 - 1 |  | 0.0021425 |  | 4.2344e-4 |  | 0.0013126 |  | 0.0029724 |  | 2187.000 |  | 5.0598048 |  | 4.5468e0-7 |  |
| timebin10 |  | 11 - 1 |  | 0.0013260 |  | 4.2344e-4 |  | 4.9612e-4 |  | 0.0021560 |  | 2187.000 |  | 3.1316159 |  | 0.0017615 |  |
| timebin11 |  | 12 - 1 |  | 7.0421e-4 |  | 4.2344e-4 |  | -1.2571e−4 |  | 0.0015341 |  | 2187.000 |  | 1.6630913 |  | 0.0964374 |  |
| timebin12 |  | 13 - 1 |  | 7.3623e-4 |  | 4.2344e-4 |  | -9.3693e−5 |  | 0.0015661 |  | 2187.000 |  | 1.7386947 |  | 0.0822292 |  |
| timebin13 |  | 14 - 1 |  | 7.3581e-4 |  | 4.2344e-4 |  | -9.4107e−5 |  | 0.0015657 |  | 2187.000 |  | 1.7377183 |  | 0.0824012 |  |
| nFrames |  | nFrames |  | 1.6826e-5 |  | 2.2443e-5 |  | -2.7161e−5 |  | 6.0813e-5 |  | 79.000 |  | 0.7497289 |  | 0.4556449 |  |
| sdWaveTime |  | sdWaveTime |  | 0.0112171 |  | 0.0063942 |  | -0.0013153 |  | 0.0237495 |  | 79.000 |  | 1.7542598 |  | 0.0832638 |  |
| directionToS1W21 ✻ timebin1 |  | 2 - 1 ✻ 2 - 1 |  | -2.4042e−4 |  | 8.4687e-4 |  | -0.0019003 |  | 0.0014194 |  | 2187.000 |  | -0.2838901 |  | 0.7765215 |  |
| directionToS1W21 ✻ timebin2 |  | 2 - 1 ✻ 3 - 1 |  | 3.7284e-4 |  | 8.4687e-4 |  | -0.0012870 |  | 0.0020327 |  | 2187.000 |  | 0.4402612 |  | 0.6597915 |  |
| directionToS1W21 ✻ timebin3 |  | 2 - 1 ✻ 4 - 1 |  | -7.5328e−6 |  | 8.4687e-4 |  | -0.0016674 |  | 0.0016523 |  | 2187.000 |  | -0.0088948 |  | 0.9929039 |  |
| directionToS1W21 ✻ timebin4 |  | 2 - 1 ✻ 5 - 1 |  | 3.0699e-4 |  | 8.4687e-4 |  | -0.0013528 |  | 0.0019668 |  | 2187.000 |  | 0.3624972 |  | 0.7170156 |  |
| directionToS1W21 ✻ timebin5 |  | 2 - 1 ✻ 6 - 1 |  | 3.1676e-4 |  | 8.4687e-4 |  | -0.0013431 |  | 0.0019766 |  | 2187.000 |  | 0.3740387 |  | 0.7084118 |  |
| directionToS1W21 ✻ timebin6 |  | 2 - 1 ✻ 7 - 1 |  | 6.3031e-4 |  | 8.4687e-4 |  | -0.0010295 |  | 0.0022902 |  | 2187.000 |  | 0.7442852 |  | 0.4567839 |  |
| directionToS1W21 ✻ timebin7 |  | 2 - 1 ✻ 8 - 1 |  | 4.6820e-4 |  | 8.4687e-4 |  | -0.0011916 |  | 0.0021280 |  | 2187.000 |  | 0.5528602 |  | 0.5804156 |  |
| directionToS1W21 ✻ timebin8 |  | 2 - 1 ✻ 9 - 1 |  | -3.1470e−4 |  | 8.4687e-4 |  | -0.0019745 |  | 0.0013451 |  | 2187.000 |  | -0.3716083 |  | 0.7102205 |  |
| directionToS1W21 ✻ timebin9 |  | 2 - 1 ✻ 10 - 1 |  | 0.0015986 |  | 8.4687e-4 |  | -6.1191e−5 |  | 0.0032585 |  | 2187.000 |  | 1.8877082 |  | 0.0591974 |  |
| directionToS1W21 ✻ timebin10 |  | 2 - 1 ✻ 11 - 1 |  | 0.0010222 |  | 8.4687e-4 |  | -6.3769e−4 |  | 0.0026820 |  | 2187.000 |  | 1.2069723 |  | 0.2275733 |  |
| directionToS1W21 ✻ timebin11 |  | 2 - 1 ✻ 12 - 1 |  | -1.3616e−4 |  | 8.4687e-4 |  | -0.0017960 |  | 0.0015237 |  | 2187.000 |  | -0.1607762 |  | 0.8722845 |  |
| directionToS1W21 ✻ timebin12 |  | 2 - 1 ✻ 13 - 1 |  | 2.9512e-4 |  | 8.4687e-4 |  | -0.0013647 |  | 0.0019550 |  | 2187.000 |  | 0.3484810 |  | 0.7275125 |  |
| directionToS1W21 ✻ timebin13 |  | 2 - 1 ✻ 14 - 1 |  | 1.0668e-4 |  | 8.4687e-4 |  | -0.0015532 |  | 0.0017665 |  | 2187.000 |  | 0.1259743 |  | 0.8997639 |  |
| AQ ✻ directionToS1W21 |  | AQ ✻ 2 - 1 |  | 1.7756e-5 |  | 2.3701e-5 |  | -2.8698e−5 |  | 6.4210e-5 |  | 2187.000 |  | 0.7491539 |  | 0.4538450 |  |
| AQ ✻ timebin1 |  | AQ ✻ 2 - 1 |  | 1.0634e-5 |  | 6.2708e-5 |  | -1.1227e−4 |  | 1.3354e-4 |  | 2187.000 |  | 0.1695748 |  | 0.8653603 |  |
| AQ ✻ timebin2 |  | AQ ✻ 3 - 1 |  | -2.2010e−5 |  | 6.2708e-5 |  | -1.4492e−4 |  | 1.0089e-4 |  | 2187.000 |  | -0.3509965 |  | 0.7256248 |  |
| AQ ✻ timebin3 |  | AQ ✻ 4 - 1 |  | -6.3264e−5 |  | 6.2708e-5 |  | -1.8617e−4 |  | 5.9641e-5 |  | 2187.000 |  | -1.0088674 |  | 0.3131498 |  |
| AQ ✻ timebin4 |  | AQ ✻ 5 - 1 |  | -1.2293e−4 |  | 6.2708e-5 |  | -2.4584e−4 |  | -2.5550e−8 |  | 2187.000 |  | -1.9603714 |  | 0.0500792 |  |
| AQ ✻ timebin5 |  | AQ ✻ 6 - 1 |  | -1.1529e−4 |  | 6.2708e-5 |  | -2.3819e−4 |  | 7.6175e-6 |  | 2187.000 |  | -1.8384885 |  | 0.0661260 |  |
| AQ ✻ timebin6 |  | AQ ✻ 7 - 1 |  | -9.3800e−5 |  | 6.2708e-5 |  | -2.1671e−4 |  | 2.9105e-5 |  | 2187.000 |  | -1.4958312 |  | 0.1348419 |  |
| AQ ✻ timebin7 |  | AQ ✻ 8 - 1 |  | -1.2736e−4 |  | 6.2708e-5 |  | -2.5026e−4 |  | -4.4519e−6 |  | 2187.000 |  | -2.0309576 |  | 0.0423800 |  |
| AQ ✻ timebin8 |  | AQ ✻ 9 - 1 |  | 7.1692e-6 |  | 6.2708e-5 |  | -1.1574e−4 |  | 1.3007e-4 |  | 2187.000 |  | 0.1143269 |  | 0.9089892 |  |
| AQ ✻ timebin9 |  | AQ ✻ 10 - 1 |  | 9.5501e-5 |  | 6.2708e-5 |  | -2.7404e−5 |  | 2.1841e-4 |  | 2187.000 |  | 1.5229507 |  | 0.1279156 |  |
| AQ ✻ timebin10 |  | AQ ✻ 11 - 1 |  | 2.8854e-5 |  | 6.2708e-5 |  | -9.4051e−5 |  | 1.5176e-4 |  | 2187.000 |  | 0.4601355 |  | 0.6454647 |  |
| AQ ✻ timebin11 |  | AQ ✻ 12 - 1 |  | 6.5585e-5 |  | 6.2708e-5 |  | -5.7320e−5 |  | 1.8849e-4 |  | 2187.000 |  | 1.0458838 |  | 0.2957302 |  |
| AQ ✻ timebin12 |  | AQ ✻ 13 - 1 |  | 6.0210e-5 |  | 6.2708e-5 |  | -6.2695e−5 |  | 1.8312e-4 |  | 2187.000 |  | 0.9601711 |  | 0.3370752 |  |
| AQ ✻ timebin13 |  | AQ ✻ 14 - 1 |  | 4.8128e-5 |  | 6.2708e-5 |  | -7.4777e−5 |  | 1.7103e-4 |  | 2187.000 |  | 0.7674989 |  | 0.4428678 |  |
| SCC ✻ directionToS1W21 |  | SCC ✻ 2 - 1 |  | 1.9132e-5 |  | 1.4989e-5 |  | -1.0247e−5 |  | 4.8510e-5 |  | 2187.000 |  | 1.2763629 |  | 0.2019628 |  |
| SCC ✻ timebin1 |  | SCC ✻ 2 - 1 |  | -1.2346e−5 |  | 3.9658e-5 |  | -9.0074e−5 |  | 6.5383e-5 |  | 2187.000 |  | -0.3113012 |  | 0.7556013 |  |
| SCC ✻ timebin2 |  | SCC ✻ 3 - 1 |  | -4.1983e−5 |  | 3.9658e-5 |  | -1.1971e−4 |  | 3.5746e-5 |  | 2187.000 |  | -1.0586151 |  | 0.2898920 |  |
| SCC ✻ timebin3 |  | SCC ✻ 4 - 1 |  | -5.3889e−5 |  | 3.9658e-5 |  | -1.3162e−4 |  | 2.3840e-5 |  | 2187.000 |  | -1.3588358 |  | 0.1743387 |  |
| SCC ✻ timebin4 |  | SCC ✻ 5 - 1 |  | -6.0991e−5 |  | 3.9658e-5 |  | -1.3872e−4 |  | 1.6737e-5 |  | 2187.000 |  | -1.5379365 |  | 0.1242088 |  |
| SCC ✻ timebin5 |  | SCC ✻ 6 - 1 |  | -6.0086e−5 |  | 3.9658e-5 |  | -1.3781e−4 |  | 1.7642e-5 |  | 2187.000 |  | -1.5151050 |  | 0.1298903 |  |
| SCC ✻ timebin6 |  | SCC ✻ 7 - 1 |  | -5.5865e−5 |  | 3.9658e-5 |  | -1.3359e−4 |  | 2.1863e-5 |  | 2187.000 |  | -1.4086660 |  | 0.1590761 |  |
| SCC ✻ timebin7 |  | SCC ✻ 8 - 1 |  | -7.2356e−5 |  | 3.9658e-5 |  | -1.5008e−4 |  | 5.3719e-6 |  | 2187.000 |  | -1.8245075 |  | 0.0682118 |  |
| SCC ✻ timebin8 |  | SCC ✻ 9 - 1 |  | -8.8793e−5 |  | 3.9658e-5 |  | -1.6652e−4 |  | -1.1065e−5 |  | 2187.000 |  | -2.2389624 |  | 0.0252586 |  |
| SCC ✻ timebin9 |  | SCC ✻ 10 - 1 |  | -9.1275e−5 |  | 3.9658e-5 |  | -1.6900e−4 |  | -1.3547e−5 |  | 2187.000 |  | -2.3015517 |  | 0.0214541 |  |
| SCC ✻ timebin10 |  | SCC ✻ 11 - 1 |  | -9.6863e−5 |  | 3.9658e-5 |  | -1.7459e−4 |  | -1.9135e−5 |  | 2187.000 |  | -2.4424663 |  | 0.0146660 |  |
| SCC ✻ timebin11 |  | SCC ✻ 12 - 1 |  | -4.7210e−5 |  | 3.9658e-5 |  | -1.2494e−4 |  | 3.0518e-5 |  | 2187.000 |  | -1.1904280 |  | 0.2340074 |  |
| SCC ✻ timebin12 |  | SCC ✻ 13 - 1 |  | -3.6540e−5 |  | 3.9658e-5 |  | -1.1427e−4 |  | 4.1189e-5 |  | 2187.000 |  | -0.9213662 |  | 0.3569609 |  |
| SCC ✻ timebin13 |  | SCC ✻ 14 - 1 |  | -4.8704e−5 |  | 3.9658e-5 |  | -1.2643e−4 |  | 2.9024e-5 |  | 2187.000 |  | -1.2281102 |  | 0.2195378 |  |
| AQ ✻ directionToS1W21 ✻ timebin1 |  | AQ ✻ 2 - 1 ✻ 2 - 1 |  | 5.0070e-5 |  | 1.2542e-4 |  | -1.9574e−4 |  | 2.9588e-4 |  | 2187.000 |  | 0.3992294 |  | 0.6897632 |  |
| AQ ✻ directionToS1W21 ✻ timebin2 |  | AQ ✻ 2 - 1 ✻ 3 - 1 |  | 7.2177e-5 |  | 1.2542e-4 |  | -1.7363e−4 |  | 3.1799e-4 |  | 2187.000 |  | 0.5755035 |  | 0.5650101 |  |
| AQ ✻ directionToS1W21 ✻ timebin3 |  | AQ ✻ 2 - 1 ✻ 4 - 1 |  | -2.7998e−6 |  | 1.2542e-4 |  | -2.4861e−4 |  | 2.4301e-4 |  | 2187.000 |  | -0.0223244 |  | 0.9821912 |  |
| AQ ✻ directionToS1W21 ✻ timebin4 |  | AQ ✻ 2 - 1 ✻ 5 - 1 |  | -5.9000e−6 |  | 1.2542e-4 |  | -2.5171e−4 |  | 2.3991e-4 |  | 2187.000 |  | -0.0470436 |  | 0.9624828 |  |
| AQ ✻ directionToS1W21 ✻ timebin5 |  | AQ ✻ 2 - 1 ✻ 6 - 1 |  | 1.0850e-5 |  | 1.2542e-4 |  | -2.3496e−4 |  | 2.5666e-4 |  | 2187.000 |  | 0.0865117 |  | 0.9310676 |  |
| AQ ✻ directionToS1W21 ✻ timebin6 |  | AQ ✻ 2 - 1 ✻ 7 - 1 |  | 2.7125e-5 |  | 1.2542e-4 |  | -2.1869e−4 |  | 2.7293e-4 |  | 2187.000 |  | 0.2162792 |  | 0.8287903 |  |
| AQ ✻ directionToS1W21 ✻ timebin7 |  | AQ ✻ 2 - 1 ✻ 8 - 1 |  | -9.1788e−6 |  | 1.2542e-4 |  | -2.5499e−4 |  | 2.3663e-4 |  | 2187.000 |  | -0.0731869 |  | 0.9416641 |  |
| AQ ✻ directionToS1W21 ✻ timebin8 |  | AQ ✻ 2 - 1 ✻ 9 - 1 |  | 1.0498e-5 |  | 1.2542e-4 |  | -2.3531e−4 |  | 2.5631e-4 |  | 2187.000 |  | 0.0837062 |  | 0.9332977 |  |
| AQ ✻ directionToS1W21 ✻ timebin9 |  | AQ ✻ 2 - 1 ✻ 10 - 1 |  | 1.7076e-4 |  | 1.2542e-4 |  | -7.5051e−5 |  | 4.1657e-4 |  | 2187.000 |  | 1.3615453 |  | 0.1734817 |  |
| AQ ✻ directionToS1W21 ✻ timebin10 |  | AQ ✻ 2 - 1 ✻ 11 - 1 |  | 1.0033e-4 |  | 1.2542e-4 |  | -1.4548e−4 |  | 3.4614e-4 |  | 2187.000 |  | 0.7999419 |  | 0.4238313 |  |
| AQ ✻ directionToS1W21 ✻ timebin11 |  | AQ ✻ 2 - 1 ✻ 12 - 1 |  | 4.8320e-5 |  | 1.2542e-4 |  | -1.9749e−4 |  | 2.9413e-4 |  | 2187.000 |  | 0.3852760 |  | 0.7000704 |  |
| AQ ✻ directionToS1W21 ✻ timebin12 |  | AQ ✻ 2 - 1 ✻ 13 - 1 |  | 6.7218e-5 |  | 1.2542e-4 |  | -1.7859e−4 |  | 3.1303e-4 |  | 2187.000 |  | 0.5359615 |  | 0.5920396 |  |
| AQ ✻ directionToS1W21 ✻ timebin13 |  | AQ ✻ 2 - 1 ✻ 14 - 1 |  | -1.9860e−5 |  | 1.2542e-4 |  | -2.6567e−4 |  | 2.2595e-4 |  | 2187.000 |  | -0.1583562 |  | 0.8741908 |  |
| SCC ✻ directionToS1W21 ✻ timebin1 |  | SCC ✻ 2 - 1 ✻ 2 - 1 |  | -7.2060e−6 |  | 7.9316e-5 |  | -1.6266e−4 |  | 1.4825e-4 |  | 2187.000 |  | -0.0908522 |  | 0.9276183 |  |
| SCC ✻ directionToS1W21 ✻ timebin2 |  | SCC ✻ 2 - 1 ✻ 3 - 1 |  | 1.0229e-5 |  | 7.9316e-5 |  | -1.4523e−4 |  | 1.6569e-4 |  | 2187.000 |  | 0.1289659 |  | 0.8973965 |  |
| SCC ✻ directionToS1W21 ✻ timebin3 |  | SCC ✻ 2 - 1 ✻ 4 - 1 |  | 5.8145e-7 |  | 7.9316e-5 |  | -1.5487e−4 |  | 1.5604e-4 |  | 2187.000 |  | 0.0073308 |  | 0.9941516 |  |
| SCC ✻ directionToS1W21 ✻ timebin4 |  | SCC ✻ 2 - 1 ✻ 5 - 1 |  | 2.6286e-5 |  | 7.9316e-5 |  | -1.2917e−4 |  | 1.8174e-4 |  | 2187.000 |  | 0.3314042 |  | 0.7403709 |  |
| SCC ✻ directionToS1W21 ✻ timebin5 |  | SCC ✻ 2 - 1 ✻ 6 - 1 |  | 1.5210e-5 |  | 7.9316e-5 |  | -1.4025e−4 |  | 1.7067e-4 |  | 2187.000 |  | 0.1917660 |  | 0.8479433 |  |
| SCC ✻ directionToS1W21 ✻ timebin6 |  | SCC ✻ 2 - 1 ✻ 7 - 1 |  | 2.6051e-5 |  | 7.9316e-5 |  | -1.2941e−4 |  | 1.8151e-4 |  | 2187.000 |  | 0.3284408 |  | 0.7426098 |  |
| SCC ✻ directionToS1W21 ✻ timebin7 |  | SCC ✻ 2 - 1 ✻ 8 - 1 |  | 3.5913e-5 |  | 7.9316e-5 |  | -1.1954e−4 |  | 1.9137e-4 |  | 2187.000 |  | 0.4527792 |  | 0.6507527 |  |
| SCC ✻ directionToS1W21 ✻ timebin8 |  | SCC ✻ 2 - 1 ✻ 9 - 1 |  | 1.5815e-5 |  | 7.9316e-5 |  | -1.3964e−4 |  | 1.7127e-4 |  | 2187.000 |  | 0.1993871 |  | 0.8419785 |  |
| SCC ✻ directionToS1W21 ✻ timebin9 |  | SCC ✻ 2 - 1 ✻ 10 - 1 |  | 3.9885e-5 |  | 7.9316e-5 |  | -1.1557e−4 |  | 1.9534e-4 |  | 2187.000 |  | 0.5028619 |  | 0.6151120 |  |
| SCC ✻ directionToS1W21 ✻ timebin10 |  | SCC ✻ 2 - 1 ✻ 11 - 1 |  | 1.0696e-4 |  | 7.9316e-5 |  | -4.8500e−5 |  | 2.6241e-4 |  | 2187.000 |  | 1.3484815 |  | 0.1776432 |  |
| SCC ✻ directionToS1W21 ✻ timebin11 |  | SCC ✻ 2 - 1 ✻ 12 - 1 |  | -1.7942e−5 |  | 7.9316e-5 |  | -1.7340e−4 |  | 1.3751e-4 |  | 2187.000 |  | -0.2262048 |  | 0.8210633 |  |
| SCC ✻ directionToS1W21 ✻ timebin12 |  | SCC ✻ 2 - 1 ✻ 13 - 1 |  | 9.0466e-5 |  | 7.9316e-5 |  | -6.4991e−5 |  | 2.4592e-4 |  | 2187.000 |  | 1.1405742 |  | 0.2541721 |  |
| SCC ✻ directionToS1W21 ✻ timebin13 |  | SCC ✻ 2 - 1 ✻ 14 - 1 |  | -6.1109e−5 |  | 7.9316e-5 |  | -2.1657e−4 |  | 9.4347e-5 |  | 2187.000 |  | -0.7704548 |  | 0.4411133 |  |
|  | | | | | | | | | | | | | | | | | |

| Random Components | | | | | | | | | |
| --- | --- | --- | --- | --- | --- | --- | --- | --- | --- |
|  |  |  |  |  |  |  |  |  |  |
| **Groups** | | **Name** | | **SD** | | **Variance** | | **ICC** | |
| anonymousId.qual |  | (Intercept) |  | 0.0084753 |  | 7.1830e-5 |  | 0.82667 |  |
| Residual |  |  |  | 0.0038809 |  | 1.5061e-5 |  |  |  |
| Note. Number of Obs: 2352 , groups: anonymousId.qual 84 | | | | | | | | | |
|  | | | | | | | | | |

Post Hoc Tests

| Post Hoc Comparisons - timebin | | | | | | | | | | | | | | | |
| --- | --- | --- | --- | --- | --- | --- | --- | --- | --- | --- | --- | --- | --- | --- | --- |
| **Comparison** | | | | | |  | | | | | | | | | |
| **timebin** | |  | | **timebin** | | **Difference** | | **SE** | | **t** | | **df** | | **p_bonferroni_** | |
| 2 |  | - |  | 10 |  | -0.0017071 |  | 4.2344e-4 |  | -4.031517 |  | 2187.0 |  | 0.0052158 |  |
| 2 |  | - |  | 11 |  | -8.9062e−4 |  | 4.2344e-4 |  | -2.103328 |  | 2187.0 |  | 1.0000000 |  |
| 2 |  | - |  | 12 |  | -2.6880e−4 |  | 4.2344e-4 |  | -0.634803 |  | 2187.0 |  | 1.0000000 |  |
| 2 |  | - |  | 13 |  | -3.0081e−4 |  | 4.2344e-4 |  | -0.710407 |  | 2187.0 |  | 1.0000000 |  |
| 2 |  | - |  | 14 |  | -3.0040e−4 |  | 4.2344e-4 |  | -0.709430 |  | 2187.0 |  | 1.0000000 |  |
| 2 |  | - |  | 3 |  | -5.8897e−5 |  | 4.2344e-4 |  | -0.139092 |  | 2187.0 |  | 1.0000000 |  |
| 2 |  | - |  | 4 |  | -9.0598e−4 |  | 4.2344e-4 |  | -2.139598 |  | 2187.0 |  | 1.0000000 |  |
| 2 |  | - |  | 6 |  | -0.0050808 |  | 4.2344e-4 |  | -11.998985 |  | 2187.0 |  | 3.2719e-30 |  |
| 2 |  | - |  | 5 |  | -0.0019847 |  | 4.2344e-4 |  | -4.687103 |  | 2187.0 |  | 2.6768e0-4 |  |
| 2 |  | - |  | 7 |  | -0.0055794 |  | 4.2344e-4 |  | -13.176466 |  | 2187.0 |  | 2.9925e-36 |  |
| 2 |  | - |  | 8 |  | -0.0013528 |  | 4.2344e-4 |  | -3.194722 |  | 2187.0 |  | 0.1291818 |  |
| 2 |  | - |  | 9 |  | -0.0026165 |  | 4.2344e-4 |  | -6.179150 |  | 2187.0 |  | 6.9745e0-8 |  |
| 1 |  | - |  | 2 |  | -4.3541e−4 |  | 4.2344e-4 |  | -1.028288 |  | 2187.0 |  | 1.0000000 |  |
| 1 |  | - |  | 10 |  | -0.0021425 |  | 4.2344e-4 |  | -5.059805 |  | 2187.0 |  | 4.1375e0-5 |  |
| 1 |  | - |  | 11 |  | -0.0013260 |  | 4.2344e-4 |  | -3.131616 |  | 2187.0 |  | 0.1602931 |  |
| 1 |  | - |  | 12 |  | -7.0421e−4 |  | 4.2344e-4 |  | -1.663091 |  | 2187.0 |  | 1.0000000 |  |
| 1 |  | - |  | 13 |  | -7.3623e−4 |  | 4.2344e-4 |  | -1.738695 |  | 2187.0 |  | 1.0000000 |  |
| 1 |  | - |  | 14 |  | -7.3581e−4 |  | 4.2344e-4 |  | -1.737718 |  | 2187.0 |  | 1.0000000 |  |
| 1 |  | - |  | 3 |  | -4.9431e−4 |  | 4.2344e-4 |  | -1.167380 |  | 2187.0 |  | 1.0000000 |  |
| 1 |  | - |  | 4 |  | -0.0013414 |  | 4.2344e-4 |  | -3.167887 |  | 2187.0 |  | 0.1416600 |  |
| 1 |  | - |  | 6 |  | -0.0055162 |  | 4.2344e-4 |  | -13.027273 |  | 2187.0 |  | 1.8522e-35 |  |
| 1 |  | - |  | 5 |  | -0.0024201 |  | 4.2344e-4 |  | -5.715391 |  | 2187.0 |  | 1.1321e0-6 |  |
| 1 |  | - |  | 7 |  | -0.0060148 |  | 4.2344e-4 |  | -14.204754 |  | 2187.0 |  | 6.5394e-42 |  |
| 1 |  | - |  | 8 |  | -0.0017882 |  | 4.2344e-4 |  | -4.223010 |  | 2187.0 |  | 0.0022835 |  |
| 1 |  | - |  | 9 |  | -0.0030519 |  | 4.2344e-4 |  | -7.207438 |  | 2187.0 |  | 7.1116e-11 |  |
| 10 |  | - |  | 11 |  | 8.1646e-4 |  | 4.2344e-4 |  | 1.928189 |  | 2187.0 |  | 1.0000000 |  |
| 10 |  | - |  | 12 |  | 0.0014383 |  | 4.2344e-4 |  | 3.396713 |  | 2187.0 |  | 0.0631724 |  |
| 10 |  | - |  | 13 |  | 0.0014063 |  | 4.2344e-4 |  | 3.321110 |  | 2187.0 |  | 0.0829325 |  |
| 10 |  | - |  | 14 |  | 0.0014067 |  | 4.2344e-4 |  | 3.322086 |  | 2187.0 |  | 0.0826443 |  |
| 11 |  | - |  | 12 |  | 6.2183e-4 |  | 4.2344e-4 |  | 1.468525 |  | 2187.0 |  | 1.0000000 |  |
| 11 |  | - |  | 13 |  | 5.8981e-4 |  | 4.2344e-4 |  | 1.392921 |  | 2187.0 |  | 1.0000000 |  |
| 11 |  | - |  | 14 |  | 5.9023e-4 |  | 4.2344e-4 |  | 1.393898 |  | 2187.0 |  | 1.0000000 |  |
| 12 |  | - |  | 13 |  | -3.2013e−5 |  | 4.2344e-4 |  | -0.075603 |  | 2187.0 |  | 1.0000000 |  |
| 12 |  | - |  | 14 |  | -3.1600e−5 |  | 4.2344e-4 |  | -0.074627 |  | 2187.0 |  | 1.0000000 |  |
| 13 |  | - |  | 14 |  | 4.1343e-7 |  | 4.2344e-4 |  | 9.7636e-4 |  | 2187.0 |  | 1.0000000 |  |
| 3 |  | - |  | 10 |  | -0.0016482 |  | 4.2344e-4 |  | -3.892425 |  | 2187.0 |  | 0.0093019 |  |
| 3 |  | - |  | 11 |  | -8.3173e−4 |  | 4.2344e-4 |  | -1.964236 |  | 2187.0 |  | 1.0000000 |  |
| 3 |  | - |  | 12 |  | -2.0990e−4 |  | 4.2344e-4 |  | -0.495711 |  | 2187.0 |  | 1.0000000 |  |
| 3 |  | - |  | 13 |  | -2.4192e−4 |  | 4.2344e-4 |  | -0.571315 |  | 2187.0 |  | 1.0000000 |  |
| 3 |  | - |  | 14 |  | -2.4150e−4 |  | 4.2344e-4 |  | -0.570338 |  | 2187.0 |  | 1.0000000 |  |
| 3 |  | - |  | 4 |  | -8.4709e−4 |  | 4.2344e-4 |  | -2.000507 |  | 2187.0 |  | 1.0000000 |  |
| 3 |  | - |  | 6 |  | -0.0050219 |  | 4.2344e-4 |  | -11.859893 |  | 2187.0 |  | 1.5706e-29 |  |
| 3 |  | - |  | 5 |  | -0.0019258 |  | 4.2344e-4 |  | -4.548011 |  | 2187.0 |  | 5.1975e0-4 |  |
| 3 |  | - |  | 7 |  | -0.0055205 |  | 4.2344e-4 |  | -13.037375 |  | 2187.0 |  | 1.6381e-35 |  |
| 3 |  | - |  | 8 |  | -0.0012939 |  | 4.2344e-4 |  | -3.055631 |  | 2187.0 |  | 0.2068439 |  |
| 3 |  | - |  | 9 |  | -0.0025576 |  | 4.2344e-4 |  | -6.040058 |  | 2187.0 |  | 1.6430e0-7 |  |
| 4 |  | - |  | 10 |  | -8.0111e−4 |  | 4.2344e-4 |  | -1.891918 |  | 2187.0 |  | 1.0000000 |  |
| 4 |  | - |  | 11 |  | 1.5358e-5 |  | 4.2344e-4 |  | 0.036271 |  | 2187.0 |  | 1.0000000 |  |
| 4 |  | - |  | 12 |  | 6.3718e-4 |  | 4.2344e-4 |  | 1.504795 |  | 2187.0 |  | 1.0000000 |  |
| 4 |  | - |  | 13 |  | 6.0517e-4 |  | 4.2344e-4 |  | 1.429192 |  | 2187.0 |  | 1.0000000 |  |
| 4 |  | - |  | 14 |  | 6.0558e-4 |  | 4.2344e-4 |  | 1.430168 |  | 2187.0 |  | 1.0000000 |  |
| 4 |  | - |  | 6 |  | -0.0041748 |  | 4.2344e-4 |  | -9.859386 |  | 2187.0 |  | 1.6576e-20 |  |
| 4 |  | - |  | 5 |  | -0.0010787 |  | 4.2344e-4 |  | -2.547505 |  | 2187.0 |  | 0.9934968 |  |
| 4 |  | - |  | 7 |  | -0.0046734 |  | 4.2344e-4 |  | -11.036868 |  | 2187.0 |  | 1.2176e-25 |  |
| 4 |  | - |  | 8 |  | -4.4678e−4 |  | 4.2344e-4 |  | -1.055124 |  | 2187.0 |  | 1.0000000 |  |
| 4 |  | - |  | 9 |  | -0.0017105 |  | 4.2344e-4 |  | -4.039551 |  | 2187.0 |  | 0.0050416 |  |
| 6 |  | - |  | 10 |  | 0.0033737 |  | 4.2344e-4 |  | 7.967468 |  | 2187.0 |  | 2.3490e-13 |  |
| 6 |  | - |  | 11 |  | 0.0041902 |  | 4.2344e-4 |  | 9.895657 |  | 2187.0 |  | 1.1723e-20 |  |
| 6 |  | - |  | 12 |  | 0.0048120 |  | 4.2344e-4 |  | 11.364181 |  | 2187.0 |  | 3.6977e-27 |  |
| 6 |  | - |  | 13 |  | 0.0047800 |  | 4.2344e-4 |  | 11.288578 |  | 2187.0 |  | 8.3544e-27 |  |
| 6 |  | - |  | 14 |  | 0.0047804 |  | 4.2344e-4 |  | 11.289554 |  | 2187.0 |  | 8.2672e-27 |  |
| 6 |  | - |  | 7 |  | -4.9859e−4 |  | 4.2344e-4 |  | -1.177482 |  | 2187.0 |  | 1.0000000 |  |
| 6 |  | - |  | 8 |  | 0.0037280 |  | 4.2344e-4 |  | 8.804262 |  | 2187.0 |  | 2.3858e-16 |  |
| 6 |  | - |  | 9 |  | 0.0024643 |  | 4.2344e-4 |  | 5.819835 |  | 2187.0 |  | 6.1499e0-7 |  |
| 5 |  | - |  | 10 |  | 2.7760e-4 |  | 4.2344e-4 |  | 0.655586 |  | 2187.0 |  | 1.0000000 |  |
| 5 |  | - |  | 11 |  | 0.0010941 |  | 4.2344e-4 |  | 2.583775 |  | 2187.0 |  | 0.8951546 |  |
| 5 |  | - |  | 12 |  | 0.0017159 |  | 4.2344e-4 |  | 4.052300 |  | 2187.0 |  | 0.0047764 |  |
| 5 |  | - |  | 13 |  | 0.0016839 |  | 4.2344e-4 |  | 3.976696 |  | 2187.0 |  | 0.0065656 |  |
| 5 |  | - |  | 14 |  | 0.0016843 |  | 4.2344e-4 |  | 3.977673 |  | 2187.0 |  | 0.0065389 |  |
| 5 |  | - |  | 6 |  | -0.0030961 |  | 4.2344e-4 |  | -7.311881 |  | 2187.0 |  | 3.3461e-11 |  |
| 5 |  | - |  | 7 |  | -0.0035947 |  | 4.2344e-4 |  | -8.489363 |  | 2187.0 |  | 3.4352e-15 |  |
| 5 |  | - |  | 8 |  | 6.3193e-4 |  | 4.2344e-4 |  | 1.492381 |  | 2187.0 |  | 1.0000000 |  |
| 5 |  | - |  | 9 |  | -6.3179e−4 |  | 4.2344e-4 |  | -1.492047 |  | 2187.0 |  | 1.0000000 |  |
| 7 |  | - |  | 10 |  | 0.0038723 |  | 4.2344e-4 |  | 9.144950 |  | 2187.0 |  | 1.2062e-17 |  |
| 7 |  | - |  | 11 |  | 0.0046888 |  | 4.2344e-4 |  | 11.073139 |  | 2187.0 |  | 8.3035e-26 |  |
| 7 |  | - |  | 12 |  | 0.0053106 |  | 4.2344e-4 |  | 12.541663 |  | 2187.0 |  | 6.1876e-33 |  |
| 7 |  | - |  | 13 |  | 0.0052786 |  | 4.2344e-4 |  | 12.466060 |  | 2187.0 |  | 1.5035e-32 |  |
| 7 |  | - |  | 14 |  | 0.0052790 |  | 4.2344e-4 |  | 12.467036 |  | 2187.0 |  | 1.4864e-32 |  |
| 7 |  | - |  | 8 |  | 0.0042266 |  | 4.2344e-4 |  | 9.981744 |  | 2187.0 |  | 5.1286e-21 |  |
| 7 |  | - |  | 9 |  | 0.0029629 |  | 4.2344e-4 |  | 6.997317 |  | 2187.0 |  | 3.1446e-10 |  |
| 8 |  | - |  | 10 |  | -3.5433e−4 |  | 4.2344e-4 |  | -0.836794 |  | 2187.0 |  | 1.0000000 |  |
| 8 |  | - |  | 11 |  | 4.6214e-4 |  | 4.2344e-4 |  | 1.091395 |  | 2187.0 |  | 1.0000000 |  |
| 8 |  | - |  | 12 |  | 0.0010840 |  | 4.2344e-4 |  | 2.559919 |  | 2187.0 |  | 0.9588055 |  |
| 8 |  | - |  | 13 |  | 0.0010519 |  | 4.2344e-4 |  | 2.484316 |  | 2187.0 |  | 1.0000000 |  |
| 8 |  | - |  | 14 |  | 0.0010524 |  | 4.2344e-4 |  | 2.485292 |  | 2187.0 |  | 1.0000000 |  |
| 8 |  | - |  | 9 |  | -0.0012637 |  | 4.2344e-4 |  | -2.984427 |  | 2187.0 |  | 0.2614043 |  |
| 9 |  | - |  | 10 |  | 9.0939e-4 |  | 4.2344e-4 |  | 2.147633 |  | 2187.0 |  | 1.0000000 |  |
| 9 |  | - |  | 11 |  | 0.0017258 |  | 4.2344e-4 |  | 4.075822 |  | 2187.0 |  | 0.0043216 |  |
| 9 |  | - |  | 12 |  | 0.0023477 |  | 4.2344e-4 |  | 5.544346 |  | 2187.0 |  | 3.0089e0-6 |  |
| 9 |  | - |  | 13 |  | 0.0023157 |  | 4.2344e-4 |  | 5.468743 |  | 2187.0 |  | 4.5950e0-6 |  |
| 9 |  | - |  | 14 |  | 0.0023161 |  | 4.2344e-4 |  | 5.469719 |  | 2187.0 |  | 4.5701e0-6 |  |
|  | | | | | | | | | | | | | | | |

Simple Effects

| Simple effects of AQ : Omnibus Tests | | | | | | | | | |
| --- | --- | --- | --- | --- | --- | --- | --- | --- | --- |
| **Moderator levels** | |  | | | | | | | |
| **timebin** | | **F** | | **Num df** | | **Den df** | | **p** | |
| 1 |  | 1.81700 |  | 1.0000 |  | 94.090 |  | 0.180935 |  |
| 2 |  | 2.01600 |  | 1.0000 |  | 94.090 |  | 0.158933 |  |
| 3 |  | 1.43700 |  | 1.0000 |  | 94.090 |  | 0.233644 |  |
| 4 |  | 0.84500 |  | 1.0000 |  | 94.090 |  | 0.360350 |  |
| 5 |  | 0.26500 |  | 1.0000 |  | 94.090 |  | 0.607853 |  |
| 6 |  | 0.32100 |  | 1.0000 |  | 94.090 |  | 0.572296 |  |
| 7 |  | 0.50700 |  | 1.0000 |  | 94.090 |  | 0.478066 |  |
| 8 |  | 0.23500 |  | 1.0000 |  | 94.090 |  | 0.628893 |  |
| 9 |  | 1.95000 |  | 1.0000 |  | 94.090 |  | 0.165863 |  |
| 10 |  | 3.98000 |  | 1.0000 |  | 94.090 |  | 0.048934 |  |
| 11 |  | 2.38200 |  | 1.0000 |  | 94.090 |  | 0.126091 |  |
| 12 |  | 3.21200 |  | 1.0000 |  | 94.090 |  | 0.076300 |  |
| 13 |  | 3.08300 |  | 1.0000 |  | 94.090 |  | 0.082364 |  |
| 14 |  | 2.80200 |  | 1.0000 |  | 94.090 |  | 0.097451 |  |
|  | | | | | | | | | |

| Simple effects of AQ : Parameter estimates | | | | | | | | | | | | | | | |
| --- | --- | --- | --- | --- | --- | --- | --- | --- | --- | --- | --- | --- | --- | --- | --- |
| **Moderator levels** | |  | | | | **95% Confidence Interval** | | | |  | | | | | |
| **timebin** | | **Estimate** | | **SE** | | **Lower** | | **Upper** | | **df** | | **t** | | **p** | |
| 1 |  | 1.9891e-4 |  | 1.4757e-4 |  | -9.4097e−5 |  | 4.9192e-4 |  | 94.090 |  | 1.34788 |  | 0.180935 |  |
| 2 |  | 2.0955e-4 |  | 1.4757e-4 |  | -8.3463e−5 |  | 5.0255e-4 |  | 94.090 |  | 1.41993 |  | 0.158933 |  |
| 3 |  | 1.7690e-4 |  | 1.4757e-4 |  | -1.1611e−4 |  | 4.6991e-4 |  | 94.090 |  | 1.19873 |  | 0.233644 |  |
| 4 |  | 1.3565e-4 |  | 1.4757e-4 |  | -1.5736e−4 |  | 4.2866e-4 |  | 94.090 |  | 0.91918 |  | 0.360350 |  |
| 5 |  | 7.5981e-5 |  | 1.4757e-4 |  | -2.1703e−4 |  | 3.6899e-4 |  | 94.090 |  | 0.51487 |  | 0.607853 |  |
| 6 |  | 8.3624e-5 |  | 1.4757e-4 |  | -2.0938e−4 |  | 3.7663e-4 |  | 94.090 |  | 0.56666 |  | 0.572296 |  |
| 7 |  | 1.0511e-4 |  | 1.4757e-4 |  | -1.8790e−4 |  | 3.9812e-4 |  | 94.090 |  | 0.71226 |  | 0.478066 |  |
| 8 |  | 7.1555e-5 |  | 1.4757e-4 |  | -2.2145e−4 |  | 3.6456e-4 |  | 94.090 |  | 0.48487 |  | 0.628893 |  |
| 9 |  | 2.0608e-4 |  | 1.4757e-4 |  | -8.6928e−5 |  | 4.9909e-4 |  | 94.090 |  | 1.39646 |  | 0.165863 |  |
| 10 |  | 2.9441e-4 |  | 1.4757e-4 |  | 1.4041e-6 |  | 5.8742e-4 |  | 94.090 |  | 1.99501 |  | 0.048934 |  |
| 11 |  | 2.2777e-4 |  | 1.4757e-4 |  | -6.5243e−5 |  | 5.2077e-4 |  | 94.090 |  | 1.54340 |  | 0.126091 |  |
| 12 |  | 2.6450e-4 |  | 1.4757e-4 |  | -2.8512e−5 |  | 5.5750e-4 |  | 94.090 |  | 1.79230 |  | 0.076300 |  |
| 13 |  | 2.5912e-4 |  | 1.4757e-4 |  | -3.3887e−5 |  | 5.5213e-4 |  | 94.090 |  | 1.75587 |  | 0.082364 |  |
| 14 |  | 2.4704e-4 |  | 1.4757e-4 |  | -4.5969e−5 |  | 5.4005e-4 |  | 94.090 |  | 1.67400 |  | 0.097451 |  |
| Note. Simple effects are estimated keeping constant other independent variable(s) in the model | | | | | | | | | | | | | | | |
|  | | | | | | | | | | | | | | | |

Effects Plots


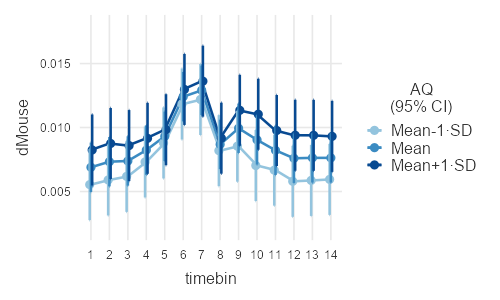


## Environment Switch ERPE Controlling for Speed – Full Statistical Results

Mixed Model

| Model Info | | | |
| --- | --- | --- | --- |
|  |  |  |  |
| **Info** | |  | |
| Estimate |  | Linear mixed model fit by REML |  |
| Call |  | avPE ~ 1 + AQ + SCC + directionToS1W2 + timebin + dMouse + nFrames + sdWaveTime + directionToS1W2:timebin + directionToS1W2:AQ + timebin:AQ + directionToS1W2:SCC + timebin:SCC + dMouse:timebin + timebin:directionToS1W2:AQ + timebin:directionToS1W2:SCC+( 1 \| anonymousId.qual ) |  |
| AIC |  | -10708.98490 |  |
| BIC |  | -8994.39970 |  |
| LogLikel. |  | 4893.11392 |  |
| R-squared Marginal |  | 0.67865 |  |
| R-squared Conditional |  | 0.83123 |  |
| Converged |  | yes |  |
| Optimizer |  | bobyqa |  |
|  | | | |

Model Results

| Fixed Effect Omnibus tests | | | | | | | | | |
| --- | --- | --- | --- | --- | --- | --- | --- | --- | --- |
|  |  |  |  |  |  |  |  |  |  |
|  | | **F** | | **Num df** | | **Den df** | | **p** | |
| AQ |  | 2.80297 |  | 1 |  | 74.036 |  | 0.0983098 |  |
| SCC |  | 2.39585 |  | 1 |  | 73.600 |  | 0.1259467 |  |
| directionToS1W2 |  | 0.40036 |  | 1 |  | 2167.608 |  | 0.5269682 |  |
| timebin |  | 51.15793 |  | 13 |  | 2175.060 |  | 6.0521e-116 |  |
| dMouse |  | 1047.07267 |  | 1 |  | 1378.771 |  | 2.2865e-171 |  |
| nFrames |  | 0.19509 |  | 1 |  | 73.762 |  | 0.6600021 |  |
| sdWaveTime |  | 7.82323 |  | 1 |  | 74.439 |  | 0.0065607 |  |
| directionToS1W2 ✻ timebin |  | 0.86296 |  | 13 |  | 2167.792 |  | 0.5925631 |  |
| AQ ✻ directionToS1W2 |  | 0.60281 |  | 1 |  | 2167.732 |  | 0.4375943 |  |
| AQ ✻ timebin |  | 2.32474 |  | 13 |  | 2168.360 |  | 0.0045727 |  |
| SCC ✻ directionToS1W2 |  | 1.30659 |  | 1 |  | 2168.197 |  | 0.2531387 |  |
| SCC ✻ timebin |  | 1.30932 |  | 13 |  | 2167.914 |  | 0.1994106 |  |
| timebin ✻ dMouse |  | 52.66481 |  | 13 |  | 2175.755 |  | 3.8292e-119 |  |
| AQ ✻ directionToS1W2 ✻ timebin |  | 1.52931 |  | 13 |  | 2167.694 |  | 0.0992565 |  |
| SCC ✻ directionToS1W2 ✻ timebin |  | 0.71188 |  | 13 |  | 2167.774 |  | 0.7531753 |  |
| Note. Satterthwaite method for degrees of freedom | | | | | | | | | |
|  | | | | | | | | | |

| Fixed Effects Parameter Estimates | | | | | | | | | | | | | | | | | |
| --- | --- | --- | --- | --- | --- | --- | --- | --- | --- | --- | --- | --- | --- | --- | --- | --- | --- |
|  | | | | | | | | **95% Confidence Interval** | | | |  | | | | | |
| **Names** | | **Effect** | | **Estimate** | | **SE** | | **Lower** | | **Upper** | | **df** | | **t** | | **p** | |
| (Intercept) |  | (Intercept) |  | 0.0708411 |  | 0.0024266 |  | 0.0660851 |  | 0.0755972 |  | 73.814 |  | 29.1934473 |  | 2.7630e0-42 |  |
| AQ |  | AQ |  | -6.1892e−4 |  | 3.6968e-4 |  | -0.0013435 |  | 1.0564e-4 |  | 74.036 |  | -1.6742067 |  | 0.0983098 |  |
| SCC |  | SCC |  | -3.5718e−4 |  | 2.3076e-4 |  | -8.0945e−4 |  | 9.5096e-5 |  | 73.600 |  | -1.5478547 |  | 0.1259467 |  |
| directionToS1W21 |  | 2 - 1 |  | -5.9838e−4 |  | 9.4568e-4 |  | -0.0024519 |  | 0.0012551 |  | 2167.608 |  | -0.6327431 |  | 0.5269682 |  |
| timebin1 |  | 2 - 1 |  | 5.7852e-4 |  | 0.0025496 |  | -0.0044187 |  | 0.0055757 |  | 2168.140 |  | 0.2269027 |  | 0.8205208 |  |
| timebin2 |  | 3 - 1 |  | 0.0019046 |  | 0.0025506 |  | -0.0030945 |  | 0.0069038 |  | 2167.963 |  | 0.7467287 |  | 0.4553083 |  |
| timebin3 |  | 4 - 1 |  | 0.0101657 |  | 0.0025404 |  | 0.0051866 |  | 0.0151448 |  | 2170.995 |  | 4.0015985 |  | 6.5026e00-5 |  |
| timebin4 |  | 5 - 1 |  | 0.0165000 |  | 0.0025524 |  | 0.0114974 |  | 0.0215027 |  | 2180.266 |  | 6.4644599 |  | 1.2506e0-10 |  |
| timebin5 |  | 6 - 1 |  | 0.0330951 |  | 0.0027133 |  | 0.0277771 |  | 0.0384131 |  | 2214.748 |  | 12.1973571 |  | 3.6265e0-33 |  |
| timebin6 |  | 7 - 1 |  | 0.0353592 |  | 0.0027409 |  | 0.0299871 |  | 0.0407313 |  | 2218.271 |  | 12.9005441 |  | 9.0642e0-37 |  |
| timebin7 |  | 8 - 1 |  | 0.0119994 |  | 0.0025417 |  | 0.0070178 |  | 0.0169810 |  | 2174.124 |  | 4.7210872 |  | 2.4953e00-6 |  |
| timebin8 |  | 9 - 1 |  | 0.0014642 |  | 0.0025583 |  | -0.0035500 |  | 0.0064785 |  | 2182.335 |  | 0.5723462 |  | 0.5671464 |  |
| timebin9 |  | 10 - 1 |  | -0.0060116 |  | 0.0025451 |  | -0.0110000 |  | -0.0010233 |  | 2176.645 |  | -2.3620251 |  | 0.0182630 |  |
| timebin10 |  | 11 - 1 |  | -0.0043351 |  | 0.0025406 |  | -0.0093146 |  | 6.4428e-4 |  | 2171.647 |  | -1.7063669 |  | 0.0880828 |  |
| timebin11 |  | 12 - 1 |  | -0.0027815 |  | 0.0025452 |  | -0.0077700 |  | 0.0022071 |  | 2168.857 |  | -1.0928213 |  | 0.2745936 |  |
| timebin12 |  | 13 - 1 |  | -0.0079402 |  | 0.0025449 |  | -0.0129281 |  | -0.0029523 |  | 2168.985 |  | -3.1200613 |  | 0.0018319 |  |
| timebin13 |  | 14 - 1 |  | -0.0077495 |  | 0.0025438 |  | -0.0127354 |  | -0.0027637 |  | 2169.448 |  | -3.0463945 |  | 0.0023439 |  |
| dMouse |  | dMouse |  | 4.0601025 |  | 0.1254725 |  | 3.8141809 |  | 4.3060241 |  | 1378.771 |  | 32.3585023 |  | 2.2865e-171 |  |
| nFrames |  | nFrames |  | -2.5914e−5 |  | 5.8670e-5 |  | -1.4091e−4 |  | 8.9077e-5 |  | 73.762 |  | -0.4416950 |  | 0.6600021 |  |
| sdWaveTime |  | sdWaveTime |  | 0.0468827 |  | 0.0167618 |  | 0.0140303 |  | 0.0797352 |  | 74.439 |  | 2.7970031 |  | 0.0065607 |  |
| directionToS1W21 ✻ timebin1 |  | 2 - 1 ✻ 2 - 1 |  | -0.0033359 |  | 0.0050040 |  | -0.0131435 |  | 0.0064716 |  | 2167.616 |  | -0.6666587 |  | 0.5050611 |  |
| directionToS1W21 ✻ timebin2 |  | 2 - 1 ✻ 3 - 1 |  | 0.0028194 |  | 0.0050031 |  | -0.0069866 |  | 0.0126254 |  | 2167.669 |  | 0.5635242 |  | 0.5731363 |  |
| directionToS1W21 ✻ timebin3 |  | 2 - 1 ✻ 4 - 1 |  | 0.0018677 |  | 0.0050033 |  | -0.0079385 |  | 0.0116740 |  | 2167.591 |  | 0.3733034 |  | 0.7089591 |  |
| directionToS1W21 ✻ timebin4 |  | 2 - 1 ✻ 5 - 1 |  | -3.0782e−5 |  | 0.0050030 |  | -0.0098366 |  | 0.0097750 |  | 2167.643 |  | -0.0061527 |  | 0.9950915 |  |
| directionToS1W21 ✻ timebin5 |  | 2 - 1 ✻ 6 - 1 |  | -0.0066368 |  | 0.0050030 |  | -0.0164426 |  | 0.0031690 |  | 2167.645 |  | -1.3265542 |  | 0.1847959 |  |
| directionToS1W21 ✻ timebin6 |  | 2 - 1 ✻ 7 - 1 |  | -0.0054951 |  | 0.0050039 |  | -0.0153025 |  | 0.0043124 |  | 2167.793 |  | -1.0981596 |  | 0.2722568 |  |
| directionToS1W21 ✻ timebin7 |  | 2 - 1 ✻ 8 - 1 |  | 1.4383e-4 |  | 0.0050035 |  | -0.0096628 |  | 0.0099505 |  | 2167.734 |  | 0.0287450 |  | 0.9770706 |  |
| directionToS1W21 ✻ timebin8 |  | 2 - 1 ✻ 9 - 1 |  | 0.0021402 |  | 0.0050037 |  | -0.0076669 |  | 0.0119472 |  | 2167.601 |  | 0.4277162 |  | 0.6689002 |  |
| directionToS1W21 ✻ timebin9 |  | 2 - 1 ✻ 10 - 1 |  | -0.0052863 |  | 0.0050096 |  | -0.0151049 |  | 0.0045322 |  | 2168.493 |  | -1.0552496 |  | 0.2914287 |  |
| directionToS1W21 ✻ timebin10 |  | 2 - 1 ✻ 11 - 1 |  | -0.0017651 |  | 0.0050061 |  | -0.0115769 |  | 0.0080467 |  | 2168.088 |  | -0.3525873 |  | 0.7244321 |  |
| directionToS1W21 ✻ timebin11 |  | 2 - 1 ✻ 12 - 1 |  | -0.0057384 |  | 0.0050035 |  | -0.0155452 |  | 0.0040683 |  | 2167.598 |  | -1.1468763 |  | 0.2515593 |  |
| directionToS1W21 ✻ timebin12 |  | 2 - 1 ✻ 13 - 1 |  | -5.0566e−4 |  | 0.0050030 |  | -0.0103114 |  | 0.0093001 |  | 2167.637 |  | -0.1010713 |  | 0.9195032 |  |
| directionToS1W21 ✻ timebin13 |  | 2 - 1 ✻ 14 - 1 |  | -0.0051895 |  | 0.0050030 |  | -0.0149952 |  | 0.0046163 |  | 2167.600 |  | -1.0372673 |  | 0.2997270 |  |
| AQ ✻ directionToS1W21 |  | AQ ✻ 2 - 1 |  | 1.0873e-4 |  | 1.4004e-4 |  | -1.6574e−4 |  | 3.8320e-4 |  | 2167.732 |  | 0.7764055 |  | 0.4375943 |  |
| AQ ✻ timebin1 |  | AQ ✻ 2 - 1 |  | 2.4043e-4 |  | 3.7400e-4 |  | -4.9259e−4 |  | 9.7346e-4 |  | 2167.593 |  | 0.6428704 |  | 0.5203762 |  |
| AQ ✻ timebin2 |  | AQ ✻ 3 - 1 |  | 1.6115e-5 |  | 3.7364e-4 |  | -7.1621e−4 |  | 7.4844e-4 |  | 2167.613 |  | 0.0431287 |  | 0.9656029 |  |
| AQ ✻ timebin3 |  | AQ ✻ 4 - 1 |  | 4.4276e-5 |  | 3.7311e-4 |  | -6.8701e−4 |  | 7.7556e-4 |  | 2167.831 |  | 0.1186668 |  | 0.9055503 |  |
| AQ ✻ timebin4 |  | AQ ✻ 5 - 1 |  | -2.5805e−4 |  | 3.7270e-4 |  | -9.8852e−4 |  | 4.7242e-4 |  | 2168.929 |  | -0.6923896 |  | 0.4887668 |  |
| AQ ✻ timebin5 |  | AQ ✻ 6 - 1 |  | -0.0011265 |  | 3.7271e-4 |  | -0.0018570 |  | -3.9599e−4 |  | 2168.949 |  | -3.0224132 |  | 0.0025370 |  |
| AQ ✻ timebin6 |  | AQ ✻ 7 - 1 |  | -9.6206e−4 |  | 3.7278e-4 |  | -0.0016927 |  | -2.3142e−4 |  | 2168.580 |  | -2.5807591 |  | 0.0099235 |  |
| AQ ✻ timebin7 |  | AQ ✻ 8 - 1 |  | -2.6816e−4 |  | 3.7269e-4 |  | -9.9863e−4 |  | 4.6230e-4 |  | 2168.955 |  | -0.7195261 |  | 0.4718943 |  |
| AQ ✻ timebin8 |  | AQ ✻ 9 - 1 |  | -8.9977e−5 |  | 3.7329e-4 |  | -8.2162e−4 |  | 6.4166e-4 |  | 2167.859 |  | -0.2410361 |  | 0.8095499 |  |
| AQ ✻ timebin9 |  | AQ ✻ 10 - 1 |  | -1.9687e−4 |  | 3.7474e-4 |  | -9.3135e−4 |  | 5.3761e-4 |  | 2167.725 |  | -0.5253392 |  | 0.5994011 |  |
| AQ ✻ timebin10 |  | AQ ✻ 11 - 1 |  | 1.2874e-4 |  | 3.7422e-4 |  | -6.0472e−4 |  | 8.6221e-4 |  | 2167.629 |  | 0.3440309 |  | 0.7308564 |  |
| AQ ✻ timebin11 |  | AQ ✻ 12 - 1 |  | 7.4563e-5 |  | 3.7520e-4 |  | -6.6081e−4 |  | 8.0994e-4 |  | 2167.926 |  | 0.1987282 |  | 0.8424940 |  |
| AQ ✻ timebin12 |  | AQ ✻ 13 - 1 |  | 4.0863e-5 |  | 3.7508e-4 |  | -6.9428e−4 |  | 7.7601e-4 |  | 2167.849 |  | 0.1089448 |  | 0.9132564 |  |
| AQ ✻ timebin13 |  | AQ ✻ 14 - 1 |  | 7.7538e-5 |  | 3.7436e-4 |  | -6.5619e−4 |  | 8.1126e-4 |  | 2167.638 |  | 0.2071250 |  | 0.8359317 |  |
| SCC ✻ directionToS1W21 |  | SCC ✻ 2 - 1 |  | 1.0128e-4 |  | 8.8600e-5 |  | -7.2377e−5 |  | 2.7493e-4 |  | 2168.197 |  | 1.1430627 |  | 0.2531387 |  |
| SCC ✻ timebin1 |  | SCC ✻ 2 - 1 |  | 5.4871e-5 |  | 2.3469e-4 |  | -4.0510e−4 |  | 5.1485e-4 |  | 2167.642 |  | 0.2338068 |  | 0.8151570 |  |
| SCC ✻ timebin2 |  | SCC ✻ 3 - 1 |  | 1.3243e-4 |  | 2.3460e-4 |  | -3.2739e−4 |  | 5.9224e-4 |  | 2168.017 |  | 0.5644823 |  | 0.5724843 |  |
| SCC ✻ timebin3 |  | SCC ✻ 4 - 1 |  | 8.9313e-5 |  | 2.3463e-4 |  | -3.7054e−4 |  | 5.4917e-4 |  | 2168.316 |  | 0.3806608 |  | 0.7034923 |  |
| SCC ✻ timebin4 |  | SCC ✻ 5 - 1 |  | 8.5354e-5 |  | 2.3466e-4 |  | -3.7457e−4 |  | 5.4527e-4 |  | 2168.549 |  | 0.3637400 |  | 0.7160876 |  |
| SCC ✻ timebin5 |  | SCC ✻ 6 - 1 |  | -1.9170e−4 |  | 2.3465e-4 |  | -6.5161e−4 |  | 2.6820e-4 |  | 2168.490 |  | -0.8169834 |  | 0.4140277 |  |
| SCC ✻ timebin6 |  | SCC ✻ 7 - 1 |  | -2.9344e−4 |  | 2.3463e-4 |  | -7.5331e−4 |  | 1.6643e-4 |  | 2168.369 |  | -1.2506243 |  | 0.2112065 |  |
| SCC ✻ timebin7 |  | SCC ✻ 8 - 1 |  | 2.3847e-4 |  | 2.3475e-4 |  | -2.2164e−4 |  | 6.9857e-4 |  | 2169.041 |  | 1.0158315 |  | 0.3098229 |  |
| SCC ✻ timebin8 |  | SCC ✻ 9 - 1 |  | 1.2026e-4 |  | 2.3477e-4 |  | -3.3989e−4 |  | 5.8041e-4 |  | 2169.114 |  | 0.5122261 |  | 0.6085449 |  |
| SCC ✻ timebin9 |  | SCC ✻ 10 - 1 |  | 1.3990e-4 |  | 2.3481e-4 |  | -3.2033e−4 |  | 6.0012e-4 |  | 2169.283 |  | 0.5957832 |  | 0.5513822 |  |
| SCC ✻ timebin10 |  | SCC ✻ 11 - 1 |  | 3.4419e-4 |  | 2.3493e-4 |  | -1.1627e−4 |  | 8.0464e-4 |  | 2169.733 |  | 1.4650691 |  | 0.1430469 |  |
| SCC ✻ timebin11 |  | SCC ✻ 12 - 1 |  | 3.9748e-4 |  | 2.3461e-4 |  | -6.2343e−5 |  | 8.5731e-4 |  | 2168.157 |  | 1.6942333 |  | 0.0903646 |  |
| SCC ✻ timebin12 |  | SCC ✻ 13 - 1 |  | 2.3717e-4 |  | 2.3461e-4 |  | -2.2265e−4 |  | 6.9699e-4 |  | 2167.944 |  | 1.0109239 |  | 0.3121657 |  |
| SCC ✻ timebin13 |  | SCC ✻ 14 - 1 |  | 2.7210e-4 |  | 2.3461e-4 |  | -1.8774e−4 |  | 7.3193e-4 |  | 2168.207 |  | 1.1597640 |  | 0.2462726 |  |
| timebin1 ✻ dMouse |  | 2 - 1 ✻ dMouse |  | -0.2413889 |  | 0.2854015 |  | -0.8007655 |  | 0.3179877 |  | 2168.123 |  | -0.8457872 |  | 0.3977648 |  |
| timebin2 ✻ dMouse |  | 3 - 1 ✻ dMouse |  | -0.4309268 |  | 0.2962072 |  | -1.0114823 |  | 0.1496287 |  | 2168.531 |  | -1.4548154 |  | 0.1458651 |  |
| timebin3 ✻ dMouse |  | 4 - 1 ✻ dMouse |  | 0.6891793 |  | 0.3040480 |  | 0.0932562 |  | 1.2851025 |  | 2171.430 |  | 2.2666792 |  | 0.0235078 |  |
| timebin4 ✻ dMouse |  | 5 - 1 ✻ dMouse |  | 1.3300778 |  | 0.3092888 |  | 0.7238829 |  | 1.9362727 |  | 2172.536 |  | 4.3004395 |  | 1.7800e00-5 |  |
| timebin5 ✻ dMouse |  | 6 - 1 ✻ dMouse |  | 2.3871658 |  | 0.2930109 |  | 1.8128750 |  | 2.9614567 |  | 2170.443 |  | 8.1470202 |  | 6.2258e0-16 |  |
| timebin6 ✻ dMouse |  | 7 - 1 ✻ dMouse |  | 2.6289115 |  | 0.2869627 |  | 2.0664749 |  | 3.1913480 |  | 2170.824 |  | 9.1611609 |  | 1.1538e0-19 |  |
| timebin7 ✻ dMouse |  | 8 - 1 ✻ dMouse |  | 0.7071192 |  | 0.3214363 |  | 0.0771157 |  | 1.3371227 |  | 2176.032 |  | 2.1998737 |  | 0.0279206 |  |
| timebin8 ✻ dMouse |  | 9 - 1 ✻ dMouse |  | -1.9006583 |  | 0.2597126 |  | -2.4096857 |  | -1.3916308 |  | 2187.666 |  | -7.3183126 |  | 3.5087e0-13 |  |
| timebin9 ✻ dMouse |  | 10 - 1 ✻ dMouse |  | -1.6356471 |  | 0.2643171 |  | -2.1536991 |  | -1.1175951 |  | 2180.013 |  | -6.1882003 |  | 7.2481e0-10 |  |
| timebin10 ✻ dMouse |  | 11 - 1 ✻ dMouse |  | -1.0462917 |  | 0.2813329 |  | -1.5976940 |  | -0.4948894 |  | 2170.586 |  | -3.7190527 |  | 2.0504e00-4 |  |
| timebin11 ✻ dMouse |  | 12 - 1 ✻ dMouse |  | -0.6815192 |  | 0.2847600 |  | -1.2396385 |  | -0.1233998 |  | 2169.569 |  | -2.3933107 |  | 0.0167816 |  |
| timebin12 ✻ dMouse |  | 13 - 1 ✻ dMouse |  | -1.0036097 |  | 0.2852778 |  | -1.5627439 |  | -0.4444756 |  | 2169.519 |  | -3.5180091 |  | 4.4374e00-4 |  |
| timebin13 ✻ dMouse |  | 14 - 1 ✻ dMouse |  | -0.8215216 |  | 0.2749449 |  | -1.3604036 |  | -0.2826395 |  | 2173.836 |  | -2.9879502 |  | 0.0028400 |  |
| AQ ✻ directionToS1W21 ✻ timebin1 |  | AQ ✻ 2 - 1 ✻ 2 - 1 |  | 2.2838e-4 |  | 7.4090e-4 |  | -0.0012238 |  | 0.0016805 |  | 2167.649 |  | 0.3082447 |  | 0.7579258 |  |
| AQ ✻ directionToS1W21 ✻ timebin2 |  | AQ ✻ 2 - 1 ✻ 3 - 1 |  | 5.5502e-5 |  | 7.4099e-4 |  | -0.0013968 |  | 0.0015078 |  | 2167.732 |  | 0.0749029 |  | 0.9402989 |  |
| AQ ✻ directionToS1W21 ✻ timebin3 |  | AQ ✻ 2 - 1 ✻ 4 - 1 |  | 8.9378e-5 |  | 7.4087e-4 |  | -0.0013627 |  | 0.0015415 |  | 2167.591 |  | 0.1206388 |  | 0.9039883 |  |
| AQ ✻ directionToS1W21 ✻ timebin4 |  | AQ ✻ 2 - 1 ✻ 5 - 1 |  | -1.3028e−4 |  | 7.4088e-4 |  | -0.0015824 |  | 0.0013218 |  | 2167.592 |  | -0.1758488 |  | 0.8604292 |  |
| AQ ✻ directionToS1W21 ✻ timebin5 |  | AQ ✻ 2 - 1 ✻ 6 - 1 |  | 0.0017376 |  | 7.4086e-4 |  | 2.8555e-4 |  | 0.0031897 |  | 2167.593 |  | 2.3453903 |  | 0.0190969 |  |
| AQ ✻ directionToS1W21 ✻ timebin6 |  | AQ ✻ 2 - 1 ✻ 7 - 1 |  | 0.0016060 |  | 7.4086e-4 |  | 1.5393e-4 |  | 0.0030581 |  | 2167.608 |  | 2.1677396 |  | 0.0302870 |  |
| AQ ✻ directionToS1W21 ✻ timebin7 |  | AQ ✻ 2 - 1 ✻ 8 - 1 |  | -5.1361e−4 |  | 7.4089e-4 |  | -0.0019657 |  | 9.3851e-4 |  | 2167.596 |  | -0.6932328 |  | 0.4882378 |  |
| AQ ✻ directionToS1W21 ✻ timebin8 |  | AQ ✻ 2 - 1 ✻ 9 - 1 |  | 1.5687e-4 |  | 7.4086e-4 |  | -0.0012952 |  | 0.0016089 |  | 2167.594 |  | 0.2117359 |  | 0.8323330 |  |
| AQ ✻ directionToS1W21 ✻ timebin9 |  | AQ ✻ 2 - 1 ✻ 10 - 1 |  | -3.1632e−4 |  | 7.4141e-4 |  | -0.0017695 |  | 0.0011368 |  | 2168.045 |  | -0.4266484 |  | 0.6696778 |  |
| AQ ✻ directionToS1W21 ✻ timebin10 |  | AQ ✻ 2 - 1 ✻ 11 - 1 |  | -1.5206e−4 |  | 7.4109e-4 |  | -0.0016046 |  | 0.0013005 |  | 2167.806 |  | -0.2051819 |  | 0.8374492 |  |
| AQ ✻ directionToS1W21 ✻ timebin11 |  | AQ ✻ 2 - 1 ✻ 12 - 1 |  | 1.3783e-4 |  | 7.4090e-4 |  | -0.0013143 |  | 0.0015900 |  | 2167.645 |  | 0.1860355 |  | 0.8524343 |  |
| AQ ✻ directionToS1W21 ✻ timebin12 |  | AQ ✻ 2 - 1 ✻ 13 - 1 |  | 2.7573e-4 |  | 7.4095e-4 |  | -0.0011765 |  | 0.0017280 |  | 2167.695 |  | 0.3721353 |  | 0.7098285 |  |
| AQ ✻ directionToS1W21 ✻ timebin13 |  | AQ ✻ 2 - 1 ✻ 14 - 1 |  | 1.9117e-5 |  | 7.4089e-4 |  | -0.0014330 |  | 0.0014712 |  | 2167.595 |  | 0.0258024 |  | 0.9794173 |  |
| SCC ✻ directionToS1W21 ✻ timebin1 |  | SCC ✻ 2 - 1 ✻ 2 - 1 |  | -3.6709e−4 |  | 4.6853e-4 |  | -0.0012854 |  | 5.5121e-4 |  | 2167.593 |  | -0.7834963 |  | 0.4334213 |  |
| SCC ✻ directionToS1W21 ✻ timebin2 |  | SCC ✻ 2 - 1 ✻ 3 - 1 |  | -7.3863e−4 |  | 4.6853e-4 |  | -0.0016569 |  | 1.7968e-4 |  | 2167.597 |  | -1.5764693 |  | 0.1150636 |  |
| SCC ✻ directionToS1W21 ✻ timebin3 |  | SCC ✻ 2 - 1 ✻ 4 - 1 |  | -6.2090e−4 |  | 4.6853e-4 |  | -0.0015392 |  | 2.9740e-4 |  | 2167.590 |  | -1.3252189 |  | 0.1852382 |  |
| SCC ✻ directionToS1W21 ✻ timebin4 |  | SCC ✻ 2 - 1 ✻ 5 - 1 |  | -3.1277e−4 |  | 4.6858e-4 |  | -0.0012312 |  | 6.0562e-4 |  | 2167.646 |  | -0.6674971 |  | 0.5045257 |  |
| SCC ✻ directionToS1W21 ✻ timebin5 |  | SCC ✻ 2 - 1 ✻ 6 - 1 |  | 3.0779e-4 |  | 4.6854e-4 |  | -6.1053e−4 |  | 0.0012261 |  | 2167.604 |  | 0.6569161 |  | 0.5113046 |  |
| SCC ✻ directionToS1W21 ✻ timebin6 |  | SCC ✻ 2 - 1 ✻ 7 - 1 |  | 3.3552e-5 |  | 4.6856e-4 |  | -8.8481e−4 |  | 9.5192e-4 |  | 2167.628 |  | 0.0716062 |  | 0.9429219 |  |
| SCC ✻ directionToS1W21 ✻ timebin7 |  | SCC ✻ 2 - 1 ✻ 8 - 1 |  | -4.2113e−4 |  | 4.6863e-4 |  | -0.0013396 |  | 4.9737e-4 |  | 2167.709 |  | -0.8986419 |  | 0.3689432 |  |
| SCC ✻ directionToS1W21 ✻ timebin8 |  | SCC ✻ 2 - 1 ✻ 9 - 1 |  | -4.3156e−4 |  | 4.6854e-4 |  | -0.0013499 |  | 4.8675e-4 |  | 2167.598 |  | -0.9210850 |  | 0.3571086 |  |
| SCC ✻ directionToS1W21 ✻ timebin9 |  | SCC ✻ 2 - 1 ✻ 10 - 1 |  | -5.1820e−4 |  | 4.6858e-4 |  | -0.0014366 |  | 4.0021e-4 |  | 2167.649 |  | -1.1058820 |  | 0.2689003 |  |
| SCC ✻ directionToS1W21 ✻ timebin10 |  | SCC ✻ 2 - 1 ✻ 11 - 1 |  | -4.9102e−4 |  | 4.6909e-4 |  | -0.0014104 |  | 4.2837e-4 |  | 2168.180 |  | -1.0467582 |  | 0.2953278 |  |
| SCC ✻ directionToS1W21 ✻ timebin11 |  | SCC ✻ 2 - 1 ✻ 12 - 1 |  | -1.5804e−4 |  | 4.6855e-4 |  | -0.0010764 |  | 7.6030e-4 |  | 2167.608 |  | -0.3372900 |  | 0.7359309 |  |
| SCC ✻ directionToS1W21 ✻ timebin12 |  | SCC ✻ 2 - 1 ✻ 13 - 1 |  | -4.0279e−4 |  | 4.6896e-4 |  | -0.0013219 |  | 5.1634e-4 |  | 2168.051 |  | -0.8589134 |  | 0.3904832 |  |
| SCC ✻ directionToS1W21 ✻ timebin13 |  | SCC ✻ 2 - 1 ✻ 14 - 1 |  | -3.6655e−4 |  | 4.6870e-4 |  | -0.0012852 |  | 5.5207e-4 |  | 2167.754 |  | -0.7820737 |  | 0.4342567 |  |
|  | | | | | | | | | | | | | | | | | |

| Random Components | | | | | | | | | |
| --- | --- | --- | --- | --- | --- | --- | --- | --- | --- |
|  |  |  |  |  |  |  |  |  |  |
| **Groups** | | **Name** | | **SD** | | **Variance** | | **ICC** | |
| anonymousId.qual |  | (Intercept) |  | 0.021797 |  | 4.7510e-4 |  | 0.47480 |  |
| Residual |  |  |  | 0.022925 |  | 5.2554e-4 |  |  |  |
| Note. Number of Obs: 2352 , groups: anonymousId.qual 84 | | | | | | | | | |
|  | | | | | | | | | |

Post Hoc Tests

| Post Hoc Comparisons - timebin | | | | | | | | | | | | | | | |
| --- | --- | --- | --- | --- | --- | --- | --- | --- | --- | --- | --- | --- | --- | --- | --- |
| **Comparison** | | | | | |  | | | | | | | | | |
| **timebin** | |  | | **timebin** | | **Difference** | | **SE** | | **t** | | **df** | | **p_bonferroni_** | |
| 2 |  | - |  | 10 |  | 0.0065902 |  | 0.0025249 |  | 2.610033 |  | 2178.0 |  | 0.8294998 |  |
| 2 |  | - |  | 11 |  | 0.0049137 |  | 0.0025222 |  | 1.948133 |  | 2174.6 |  | 1.0000000 |  |
| 2 |  | - |  | 12 |  | 0.0033600 |  | 0.0025286 |  | 1.328788 |  | 2173.2 |  | 1.0000000 |  |
| 2 |  | - |  | 13 |  | 0.0085187 |  | 0.0025282 |  | 3.369523 |  | 2173.2 |  | 0.0697184 |  |
| 2 |  | - |  | 14 |  | 0.0083281 |  | 0.0025268 |  | 3.295944 |  | 2173.4 |  | 0.0906992 |  |
| 2 |  | - |  | 3 |  | -0.0013261 |  | 0.0025351 |  | -0.523091 |  | 2173.1 |  | 1.0000000 |  |
| 2 |  | - |  | 4 |  | -0.0095872 |  | 0.0025224 |  | -3.800798 |  | 2174.2 |  | 0.0134860 |  |
| 2 |  | - |  | 6 |  | -0.0325166 |  | 0.0026864 |  | -12.103975 |  | 2211.3 |  | 9.6635e-31 |  |
| 2 |  | - |  | 5 |  | -0.0159215 |  | 0.0025312 |  | -6.289992 |  | 2180.8 |  | 3.4804e0-8 |  |
| 2 |  | - |  | 7 |  | -0.0347807 |  | 0.0027135 |  | -12.817527 |  | 2214.7 |  | 2.2511e-34 |  |
| 2 |  | - |  | 8 |  | -0.0114209 |  | 0.0025223 |  | -4.527958 |  | 2176.2 |  | 5.7123e0-4 |  |
| 2 |  | - |  | 9 |  | -8.8573e−4 |  | 0.0025367 |  | -0.349169 |  | 2182.4 |  | 1.0000000 |  |
| 1 |  | - |  | 2 |  | -5.7852e−4 |  | 0.0025496 |  | -0.226902 |  | 2173.5 |  | 1.0000000 |  |
| 1 |  | - |  | 10 |  | 0.0060116 |  | 0.0025452 |  | 2.361923 |  | 2181.5 |  | 1.0000000 |  |
| 1 |  | - |  | 11 |  | 0.0043351 |  | 0.0025406 |  | 1.706334 |  | 2176.8 |  | 1.0000000 |  |
| 1 |  | - |  | 12 |  | 0.0027815 |  | 0.0025452 |  | 1.092815 |  | 2174.2 |  | 1.0000000 |  |
| 1 |  | - |  | 13 |  | 0.0079402 |  | 0.0025449 |  | 3.120041 |  | 2174.3 |  | 0.1667108 |  |
| 1 |  | - |  | 14 |  | 0.0077495 |  | 0.0025439 |  | 3.046368 |  | 2174.8 |  | 0.2133104 |  |
| 1 |  | - |  | 3 |  | -0.0019046 |  | 0.0025506 |  | -0.746727 |  | 2173.4 |  | 1.0000000 |  |
| 1 |  | - |  | 4 |  | -0.0101657 |  | 0.0025405 |  | -4.001534 |  | 2176.2 |  | 0.0059185 |  |
| 1 |  | - |  | 6 |  | -0.0330951 |  | 0.0027140 |  | -12.194182 |  | 2217.2 |  | 3.4142e-31 |  |
| 1 |  | - |  | 5 |  | -0.0165000 |  | 0.0025526 |  | -6.464063 |  | 2184.9 |  | 1.1405e0-8 |  |
| 1 |  | - |  | 7 |  | -0.0353592 |  | 0.0027417 |  | -12.896869 |  | 2220.5 |  | 8.5979e-35 |  |
| 1 |  | - |  | 8 |  | -0.0119994 |  | 0.0025417 |  | -4.720940 |  | 2179.2 |  | 2.2720e0-4 |  |
| 1 |  | - |  | 9 |  | -0.0014642 |  | 0.0025585 |  | -0.572305 |  | 2186.9 |  | 1.0000000 |  |
| 10 |  | - |  | 11 |  | -0.0016765 |  | 0.0025053 |  | -0.669177 |  | 2174.1 |  | 1.0000000 |  |
| 10 |  | - |  | 12 |  | -0.0032302 |  | 0.0025172 |  | -1.283273 |  | 2176.6 |  | 1.0000000 |  |
| 10 |  | - |  | 13 |  | 0.0019285 |  | 0.0025164 |  | 0.766385 |  | 2176.4 |  | 1.0000000 |  |
| 10 |  | - |  | 14 |  | 0.0017379 |  | 0.0025139 |  | 0.691323 |  | 2175.8 |  | 1.0000000 |  |
| 11 |  | - |  | 12 |  | -0.0015537 |  | 0.0025154 |  | -0.617675 |  | 2173.8 |  | 1.0000000 |  |
| 11 |  | - |  | 13 |  | 0.0036050 |  | 0.0025148 |  | 1.433529 |  | 2173.7 |  | 1.0000000 |  |
| 11 |  | - |  | 14 |  | 0.0034144 |  | 0.0025128 |  | 1.358823 |  | 2173.5 |  | 1.0000000 |  |
| 12 |  | - |  | 13 |  | 0.0051587 |  | 0.0025221 |  | 2.045400 |  | 2173.0 |  | 1.0000000 |  |
| 12 |  | - |  | 14 |  | 0.0049681 |  | 0.0025205 |  | 1.971044 |  | 2173.1 |  | 1.0000000 |  |
| 13 |  | - |  | 14 |  | -1.9064e−4 |  | 0.0025200 |  | -0.075651 |  | 2173.1 |  | 1.0000000 |  |
| 3 |  | - |  | 10 |  | 0.0079163 |  | 0.0025269 |  | 3.132775 |  | 2178.6 |  | 0.1596721 |  |
| 3 |  | - |  | 11 |  | 0.0062398 |  | 0.0025238 |  | 2.472333 |  | 2174.9 |  | 1.0000000 |  |
| 3 |  | - |  | 12 |  | 0.0046861 |  | 0.0025299 |  | 1.852311 |  | 2173.3 |  | 1.0000000 |  |
| 3 |  | - |  | 13 |  | 0.0098448 |  | 0.0025295 |  | 3.892039 |  | 2173.4 |  | 0.0093183 |  |
| 3 |  | - |  | 14 |  | 0.0096542 |  | 0.0025281 |  | 3.818708 |  | 2173.6 |  | 0.0125497 |  |
| 3 |  | - |  | 4 |  | -0.0082611 |  | 0.0025239 |  | -3.273079 |  | 2174.5 |  | 0.0983250 |  |
| 3 |  | - |  | 6 |  | -0.0311905 |  | 0.0026901 |  | -11.594452 |  | 2212.5 |  | 2.9337e-28 |  |
| 3 |  | - |  | 5 |  | -0.0145954 |  | 0.0025335 |  | -5.761066 |  | 2181.5 |  | 8.6832e0-7 |  |
| 3 |  | - |  | 7 |  | -0.0334546 |  | 0.0027174 |  | -12.311267 |  | 2215.8 |  | 8.8421e-32 |  |
| 3 |  | - |  | 8 |  | -0.0100948 |  | 0.0025241 |  | -3.999344 |  | 2176.7 |  | 0.0059731 |  |
| 3 |  | - |  | 9 |  | 4.4037e-4 |  | 0.0025390 |  | 0.173445 |  | 2183.3 |  | 1.0000000 |  |
| 4 |  | - |  | 10 |  | 0.0161774 |  | 0.0025065 |  | 6.454049 |  | 2174.4 |  | 1.2181e0-8 |  |
| 4 |  | - |  | 11 |  | 0.0145009 |  | 0.0025068 |  | 5.784667 |  | 2173.1 |  | 7.5678e0-7 |  |
| 4 |  | - |  | 12 |  | 0.0129472 |  | 0.0025158 |  | 5.146410 |  | 2173.6 |  | 2.6332e0-5 |  |
| 4 |  | - |  | 13 |  | 0.0181059 |  | 0.0025152 |  | 7.198571 |  | 2173.5 |  | 7.5927e-11 |  |
| 4 |  | - |  | 14 |  | 0.0179153 |  | 0.0025133 |  | 7.128312 |  | 2173.3 |  | 1.2530e-10 |  |
| 4 |  | - |  | 6 |  | -0.0229294 |  | 0.0026563 |  | -8.632186 |  | 2202.3 |  | 1.0315e-15 |  |
| 4 |  | - |  | 5 |  | -0.0063343 |  | 0.0025113 |  | -2.522369 |  | 2176.1 |  | 1.0000000 |  |
| 4 |  | - |  | 7 |  | -0.0251935 |  | 0.0026824 |  | -9.392007 |  | 2205.7 |  | 1.2897e-18 |  |
| 4 |  | - |  | 8 |  | -0.0018337 |  | 0.0025052 |  | -0.731948 |  | 2173.5 |  | 1.0000000 |  |
| 4 |  | - |  | 9 |  | 0.0087015 |  | 0.0025159 |  | 3.458655 |  | 2177.2 |  | 0.0503519 |  |
| 6 |  | - |  | 10 |  | 0.0391068 |  | 0.0026372 |  | 14.829162 |  | 2192.9 |  | 1.5936e-45 |  |
| 6 |  | - |  | 11 |  | 0.0374303 |  | 0.0026531 |  | 14.108278 |  | 2200.9 |  | 2.2408e-41 |  |
| 6 |  | - |  | 12 |  | 0.0358766 |  | 0.0026751 |  | 13.411164 |  | 2208.3 |  | 1.5893e-37 |  |
| 6 |  | - |  | 13 |  | 0.0410353 |  | 0.0026737 |  | 15.347853 |  | 2207.8 |  | 1.2403e-48 |  |
| 6 |  | - |  | 14 |  | 0.0408447 |  | 0.0026691 |  | 15.302578 |  | 2206.3 |  | 2.3370e-48 |  |
| 6 |  | - |  | 7 |  | -0.0022641 |  | 0.0027249 |  | -0.830887 |  | 2173.2 |  | 1.0000000 |  |
| 6 |  | - |  | 8 |  | 0.0210957 |  | 0.0026432 |  | 7.981152 |  | 2196.5 |  | 2.1051e-13 |  |
| 6 |  | - |  | 9 |  | 0.0316309 |  | 0.0026323 |  | 12.016657 |  | 2186.7 |  | 2.6784e-30 |  |
| 5 |  | - |  | 10 |  | 0.0225117 |  | 0.0025047 |  | 8.987815 |  | 2173.4 |  | 4.8617e-17 |  |
| 5 |  | - |  | 11 |  | 0.0208352 |  | 0.0025098 |  | 8.301637 |  | 2175.5 |  | 1.6197e-14 |  |
| 5 |  | - |  | 12 |  | 0.0192815 |  | 0.0025229 |  | 7.642440 |  | 2179.0 |  | 2.8868e-12 |  |
| 5 |  | - |  | 13 |  | 0.0244402 |  | 0.0025221 |  | 9.690340 |  | 2178.8 |  | 8.2361e-20 |  |
| 5 |  | - |  | 14 |  | 0.0242496 |  | 0.0025193 |  | 9.625497 |  | 2178.0 |  | 1.5115e-19 |  |
| 5 |  | - |  | 6 |  | -0.0165951 |  | 0.0026324 |  | -6.304107 |  | 2188.7 |  | 3.1806e0-8 |  |
| 5 |  | - |  | 7 |  | -0.0188592 |  | 0.0026568 |  | -7.098566 |  | 2191.6 |  | 1.5431e-10 |  |
| 5 |  | - |  | 8 |  | 0.0045006 |  | 0.0025056 |  | 1.796210 |  | 2174.1 |  | 1.0000000 |  |
| 5 |  | - |  | 9 |  | 0.0150358 |  | 0.0025098 |  | 5.990865 |  | 2173.1 |  | 2.2172e0-7 |  |
| 7 |  | - |  | 10 |  | 0.0413709 |  | 0.0026621 |  | 15.540490 |  | 2196.0 |  | 8.6310e-50 |  |
| 7 |  | - |  | 11 |  | 0.0396943 |  | 0.0026790 |  | 14.816783 |  | 2204.2 |  | 1.8376e-45 |  |
| 7 |  | - |  | 12 |  | 0.0381407 |  | 0.0027018 |  | 14.116788 |  | 2211.7 |  | 1.9666e-41 |  |
| 7 |  | - |  | 13 |  | 0.0432994 |  | 0.0027003 |  | 16.035098 |  | 2211.2 |  | 7.2262e-53 |  |
| 7 |  | - |  | 14 |  | 0.0431088 |  | 0.0026956 |  | 15.992415 |  | 2209.7 |  | 1.3423e-52 |  |
| 7 |  | - |  | 8 |  | 0.0233598 |  | 0.0026686 |  | 8.753494 |  | 2199.8 |  | 3.6754e-16 |  |
| 7 |  | - |  | 9 |  | 0.0338950 |  | 0.0026563 |  | 12.760076 |  | 2189.5 |  | 4.6247e-34 |  |
| 8 |  | - |  | 10 |  | 0.0180111 |  | 0.0025020 |  | 7.198601 |  | 2173.2 |  | 7.5913e-11 |  |
| 8 |  | - |  | 11 |  | 0.0163346 |  | 0.0025042 |  | 6.522811 |  | 2173.3 |  | 7.7865e0-9 |  |
| 8 |  | - |  | 12 |  | 0.0147809 |  | 0.0025149 |  | 5.877231 |  | 2175.1 |  | 4.3821e0-7 |  |
| 8 |  | - |  | 13 |  | 0.0199396 |  | 0.0025143 |  | 7.930589 |  | 2174.9 |  | 3.1449e-13 |  |
| 8 |  | - |  | 14 |  | 0.0197490 |  | 0.0025120 |  | 7.861991 |  | 2174.5 |  | 5.3690e-13 |  |
| 8 |  | - |  | 9 |  | 0.0105352 |  | 0.0025096 |  | 4.197872 |  | 2174.8 |  | 0.0025505 |  |
| 9 |  | - |  | 10 |  | 0.0074759 |  | 0.0025083 |  | 2.980430 |  | 2173.8 |  | 0.2648431 |  |
| 9 |  | - |  | 11 |  | 0.0057994 |  | 0.0025142 |  | 2.306622 |  | 2176.6 |  | 1.0000000 |  |
| 9 |  | - |  | 12 |  | 0.0042457 |  | 0.0025281 |  | 1.679386 |  | 2180.5 |  | 1.0000000 |  |
| 9 |  | - |  | 13 |  | 0.0094044 |  | 0.0025273 |  | 3.721145 |  | 2180.2 |  | 0.0185039 |  |
| 9 |  | - |  | 14 |  | 0.0092138 |  | 0.0025244 |  | 3.649965 |  | 2179.3 |  | 0.0244286 |  |
|  | | | | | | | | | | | | | | | |

## Simple Effects

| Simple effects of AQ : Omnibus Tests | | | | | | | | | |
| --- | --- | --- | --- | --- | --- | --- | --- | --- | --- |
| **Moderator levels** | |  | | | | | | | |
| **timebin** | | **F** | | **Num df** | | **Den df** | | **p** | |
| 1 |  | 1.03100 |  | 1.0000 |  | 171.12 |  | 0.3113966 |  |
| 2 |  | 0.23100 |  | 1.0000 |  | 171.07 |  | 0.6317331 |  |
| 3 |  | 0.96100 |  | 1.0000 |  | 170.53 |  | 0.3282957 |  |
| 4 |  | 0.84500 |  | 1.0000 |  | 169.59 |  | 0.3593815 |  |
| 5 |  | 2.54900 |  | 1.0000 |  | 168.54 |  | 0.1122509 |  |
| 6 |  | 12.51600 |  | 1.0000 |  | 168.55 |  | 5.2142e-4 |  |
| 7 |  | 10.04200 |  | 1.0000 |  | 168.78 |  | 0.0018165 |  |
| 8 |  | 2.62200 |  | 1.0000 |  | 168.52 |  | 0.1072881 |  |
| 9 |  | 1.48400 |  | 1.0000 |  | 169.75 |  | 0.2247890 |  |
| 10 |  | 2.10600 |  | 1.0000 |  | 172.07 |  | 0.1485362 |  |
| 11 |  | 0.53100 |  | 1.0000 |  | 171.31 |  | 0.4673078 |  |
| 12 |  | 0.71700 |  | 1.0000 |  | 172.79 |  | 0.3981747 |  |
| 13 |  | 0.85000 |  | 1.0000 |  | 172.60 |  | 0.3577746 |  |
| 14 |  | 0.70900 |  | 1.0000 |  | 171.49 |  | 0.4008545 |  |
|  | | | | | | | | | |

| Simple effects of AQ : Parameter estimates | | | | | | | | | | | | | | | |
| --- | --- | --- | --- | --- | --- | --- | --- | --- | --- | --- | --- | --- | --- | --- | --- |
| **Moderator levels** | |  | | | | **95% Confidence Interval** | | | |  | | | | | |
| **timebin** | | **Estimate** | | **SE** | | **Lower** | | **Upper** | | **df** | | **t** | | **p** | |
| 1 |  | -4.5613e−4 |  | 4.4926e-4 |  | -0.0013429 |  | 4.3067e-4 |  | 171.12 |  | -1.01530 |  | 0.3113966 |  |
| 2 |  | -2.1570e−4 |  | 4.4923e-4 |  | -0.0011024 |  | 6.7104e-4 |  | 171.07 |  | -0.48015 |  | 0.6317331 |  |
| 3 |  | -4.4002e−4 |  | 4.4883e-4 |  | -0.0013260 |  | 4.4596e-4 |  | 170.53 |  | -0.98036 |  | 0.3282957 |  |
| 4 |  | -4.1185e−4 |  | 4.4814e-4 |  | -0.0012965 |  | 4.7279e-4 |  | 169.59 |  | -0.91904 |  | 0.3593815 |  |
| 5 |  | -7.1418e−4 |  | 4.4734e-4 |  | -0.0015973 |  | 1.6893e-4 |  | 168.54 |  | -1.59650 |  | 0.1122509 |  |
| 6 |  | -0.0015826 |  | 4.4735e-4 |  | -0.0024657 |  | -6.9949e−4 |  | 168.55 |  | -3.53779 |  | 5.2142e-4 |  |
| 7 |  | -0.0014182 |  | 4.4752e-4 |  | -0.0023017 |  | -5.3473e−4 |  | 168.78 |  | -3.16898 |  | 0.0018165 |  |
| 8 |  | -7.2429e−4 |  | 4.4733e-4 |  | -0.0016074 |  | 1.5881e-4 |  | 168.52 |  | -1.61914 |  | 0.1072881 |  |
| 9 |  | -5.4611e−4 |  | 4.4824e-4 |  | -0.0014310 |  | 3.3874e-4 |  | 169.75 |  | -1.21833 |  | 0.2247890 |  |
| 10 |  | -6.5300e−4 |  | 4.4996e-4 |  | -0.0015412 |  | 2.3516e-4 |  | 172.07 |  | -1.45123 |  | 0.1485362 |  |
| 11 |  | -3.2739e−4 |  | 4.4940e-4 |  | -0.0012145 |  | 5.5969e-4 |  | 171.31 |  | -0.72849 |  | 0.4673078 |  |
| 12 |  | -3.8157e−4 |  | 4.5050e-4 |  | -0.0012708 |  | 5.0762e-4 |  | 172.79 |  | -0.84699 |  | 0.3981747 |  |
| 13 |  | -4.1527e−4 |  | 4.5036e-4 |  | -0.0013042 |  | 4.7365e-4 |  | 172.60 |  | -0.92208 |  | 0.3577746 |  |
| 14 |  | -3.7859e−4 |  | 4.4953e-4 |  | -0.0012659 |  | 5.0874e-4 |  | 171.49 |  | -0.84219 |  | 0.4008545 |  |
| Note. Simple effects are estimated keeping constant other independent variable(s) in the model | | | | | | | | | | | | | | | |
|  | | | | | | | | | | | | | | | |

## Effects Plots


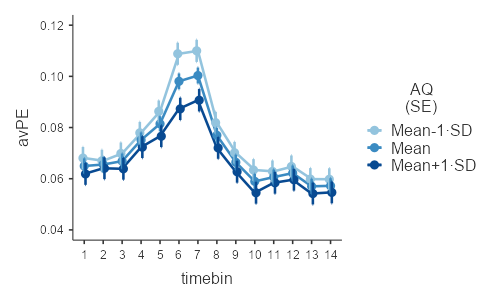

Supplement: Supplementary file 1 — (DOCX 898 kb) [file 13423_2022_2187_MOESM1_ESM.docx]
